# Cost-effectiveness of PD-1 inhibitors combined with chemotherapy for first-line treatment of oesophageal squamous cell carcinoma in China: a comprehensive analysis

**DOI:** 10.1080/07853890.2025.2482019

**Published:** 2025-03-25

**Authors:** Kai Xu, Man Yu, Qingli Sun, Lingli Zhang, Xiaodan Qian, Dan Su, Jinhong Gong, Jingjing Shang, Yingtao Lin, Xin Li

**Affiliations:** a Department of Pharmacy, The Second People’s Hospital of Changzhou, The Third Affiliated Hospital of Nanjing Medical University, Changzhou, Jiangsu Province, China; b Department of Pharmaceutical Regulatory Science and Pharmacoeconomics, School of Pharmacy, Nanjing Medical University, Nanjing, Jiangsu Province, China; c Department of Pharmacy, Qingdao Central Hospital, University of Health and Rehabilitation Sciences, Qingdao, Shandong Province, China; d School of International Pharmaceutical Business, China Pharmaceutical University, Nanjing, Jiangsu Province, China; e Center for Global Health, School of Public Health, Nanjing Medical University, Nanjing, Jiangsu Province, China; f Clinical Medical Research Center, Clinical Oncology School of Fujian Medical University, Fujian Cancer Hospital, Fuzhou, Fujian Province, China; g Department of Health Policy, School of Health Policy and Management, Nanjing Medical University, Nanjing, Jiangsu Province, China

**Keywords:** Cost-effectiveness, fractional polynomials, immune checkpoint inhibitors, oesophageal squamous cell carcinoma

## Abstract

**Background:**

Programmed death-1 (PD-1) inhibitors combined with chemotherapy have become a standard first-line treatment for advanced oesophageal squamous cell carcinoma (ESCC). Given the high costs associated with immunotherapy, evaluating the cost-effectiveness of different PD-1 inhibitors in the Chinese healthcare setting is essential for guiding treatment decisions and policy development.

**Methods:**

A cost-effectiveness analysis was conducted comparing six PD-1 inhibitors—sintilimab, toripalimab, tislelizumab, camrelizumab, serplulimab, and pembrolizumab—combined with chemotherapy for first-line treatment of advanced ESCC. A partitioned survival model was used to calculate incremental cost-effectiveness ratios (ICERs) from healthcare system perspective, with a willingness-to-pay (WTP) threshold set at $36,598.19 per quality-adjusted life year (QALY). Sensitivity analyses were performed to evaluate the robustness of the results.

**Results:**

The ICERs for toripalimab, camrelizumab, pembrolizumab, serplulimab, sintilimab, and tislelizumab were $32,356.79/QALY, $48,410.64/QALY, $312,743.54/QALY, $121,200.84/QALY, $29,663.42/QALY, and $35,304.33/QALY, respectively. Sintilimab, toripalimab, and tislelizumab were below the WTP threshold. Among all regimens, the top three in life years (LYs) gained were toripalimab, serplulimab, and tislelizumab. Sensitivity analysis showed that utility values and drug prices were key factors influencing ICERs. Probabilistic analysis indicated that toripalimab, sintilimab, and tislelizumab had the highest probabilities of being cost-effective, at 83.1%, 81.4%, and 70.0%, respectively.

**Conclusion:**

Sintilimab, toripalimab, and tislelizumab are the most cost-effective PD-1 inhibitors when combined with chemotherapy for the first-line treatment of advanced ESCC in China, with ICERs below the WTP threshold. While all six PD-1 inhibitors demonstrated clinical benefits, pembrolizumab and serplulimab were less favourable from a cost-effectiveness standpoint. Sensitivity analysis confirmed that drug prices and utility values are significant determinants of cost-effectiveness.

## Introduction

In 2020, approximately 604,100 new cases of oesophageal cancer (EC) were diagnosed globally, leading to 544,100 deaths. The age-standardized incidences and mortality rates for EC in 2020 were 6.3 and 5.6 per 1,000, respectively. Among EC cases, oesophageal squamous cell carcinoma (ESCC) accounted for 85% (512,500 cases), while oesophageal adenocarcinoma (EAC) represented 14% (85,700 cases) [1]. In China, 253,000 new EC cases EC and 154,000 deaths were reported in 2016, ranking it as the sixth most common cancer and the fifth leading cause of cancer-related mortality [2]. According to the Global Burden of Disease (GBD) statistics in 2019, the disability-adjusted life years (DALYs) due to cancer in China amounted to 67,340,309, with EC accounting for 5,759,997 DALYs, representing 8.6% of the total [3]. It is estimated that direct medical expenditures for EC in China will increase by 128.7% from 2013 to 2030, rising from 33.4 billion USD to 76.4 billion USD [4].

ESCC accounts for a substantial portion of EC in China and is associated with a poor prognosis. The five-year survival rate in non-metastatic patients is estimated at 20–35% [5,6]. Unfortunately, the majority of patients are diagnosed at an advanced or metastatic stage, precluding surgical intervention [7]. Conventional radiotherapy and chemotherapy offer limited efficacy and are often associated with severe adverse reactions [8]. Targeted therapy requires a high degree of molecular profiling specificity and is not suitable for all patients [9]. In recent years, growing attention has been directed towards immune evasion mechanisms within the tumour microenvironment [10]. This has led to the development of immune checkpoint inhibitors (ICIs), which have significantly prolonged survival in patients with advanced-stage malignancies, including EC [11–13].

The substantial efficacy and safety of ICIs such as toripalimab, camrelizumab, pembrolizumab, serplulimab, sintilimab, and tislelizumab have been validated as a first-line treatment for advanced or metastatic ESCC in clinical trials [14–19]. Previous economic evaluations have typically analysed the cost-effectiveness of individual ICIs compared to chemotherapy based on the clinical trial setting [20–25]. However, identifying the optimal among all available ICIs for patients with advanced or metastatic ESCC is also important. Liu et al. conducted an economic analysis of PD-1/PD-L1 drugs for first-line treatment of advanced ESCC [26]. However, this study employed a proportional hazards (PH) model in the meta-analysis without testing the PH assumption. Notably, many drugs exhibit delayed effects or a reduction in efficacy after a defined period of treatment in the field of cancer immunotherapy, thereby refuting the PH assumption [27–29]. Additionally, the newly marketed tislelizumab was not included in this study. To the best of our knowledge, no prior economic evaluation has assessed the cost-effectiveness of these six ICIs under the non-proportional hazards (n-PH) assumption. Therefore, we conducted a cost-effectiveness analysis of toripalimab, camrelizumab, pembrolizumab, serplulimab, sintilimab, and tislelizumab in patients with advanced or metastatic ESCC. This analysis utilized a network meta-analysis based on a fractional polynomial (FP) model, allowing for a more comprehensive and methodologically robust evaluation.

## Method

### Retrieval

**Figure 1.**
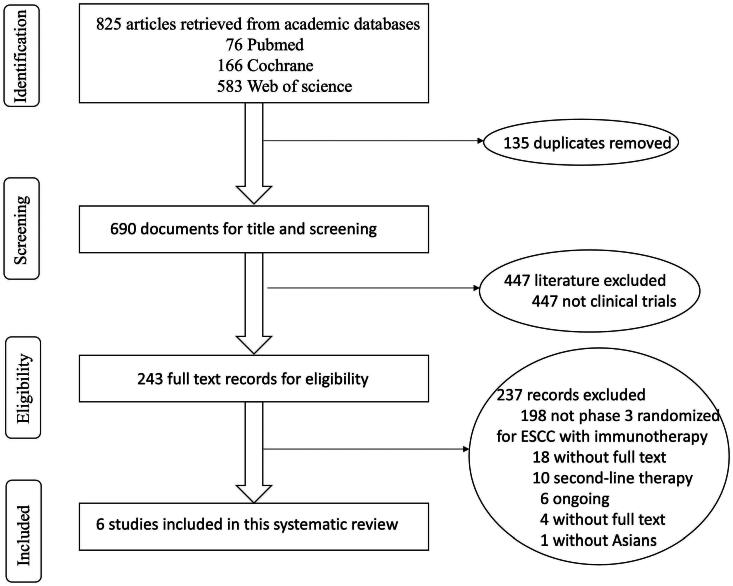
Literature screening process.

We conducted a comprehensive search for clinical trials of immunotherapy for ESCC in PubMed, Web of Science, and the Cochrane Library, spanning the period from January 2, 2015, to November 30, 2023. This study was conducted in accordance with the Preferred Reporting Items for Systematic Reviews and Meta-Analyses (PRISMA) guidelines to ensure transparency and completeness in reporting. Detailed information on the search strategy and methodology can be found in Supplementary Material and Figure 1.

### Patients and treatment

The inclusion criteria for clinical trials were as follows: adult patients with previously untreated, newly diagnosed tumours histologically or cytologically confirmed as locally advanced, unresectable, or metastatic ESCC; treatment intervention involving ICIs; inclusion of many Asians in the study population; large-scale phase-III clinical trials; mature data on overall survival (OS) and progression-free survival (PFS) using Kaplan–Meier curves. Duplicate publications, conference abstracts, and narrative reviews were excluded. Finally, six clinical trials were included in our analysis: JUPITER-06, ESCORT-1st, KEYNOTE-590, ASTRUM-007, ORIENT-15, and RATIONALE-306 [14–19]. Detailed information regarding treatment methods, sample sizes, and efficacy for each trial can be found in Table S1. We selected the control group of KEYNOTE-590, who received 5-Fluorouracil and Cisplatin, as the comparator group for our meta-analysis.

### Data processing and evaluation

We employed GetData Graph Digitize, R 4.3.2, Excel 2021, and Origin 2021 for data retrieval, meta-analysis, model development, and graphical representation.

**Figure 2.**
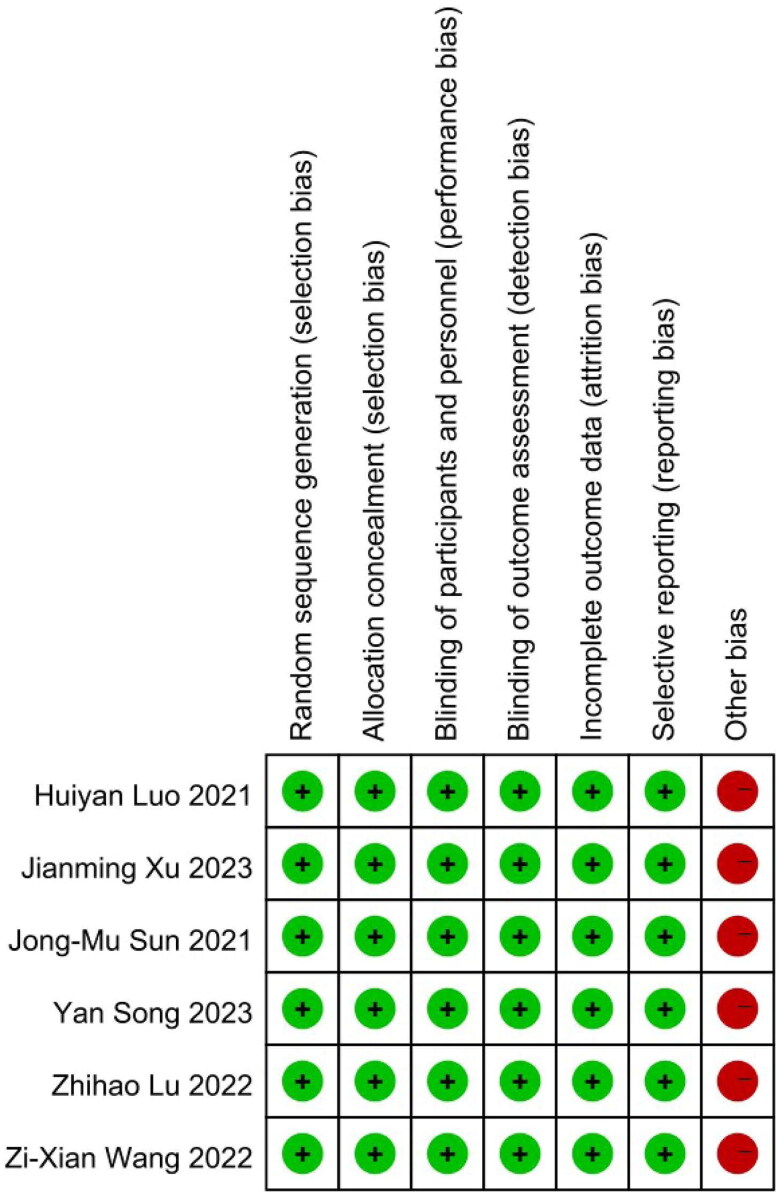
Documents quality evaluation.

Initially, we employed the Cochrane Collaboration’s tool for assessing risk of bias to appraise the included studies, with the outcomes displayed in Figure 2. Subsequently, a one-sample t-test was conducted on the baseline characteristics of the patients participating in the trials, as detailed in Table S2. Key indicators including disease status, PD-L1 status, and age exhibited no significant differences statistically, thus enabling direct comparison without adjusting for baseline levels [30,31]. Drawing from the method outlined by Guyot et al. [32]. We have retrieved data points from the PFS and OS curves of the six studies and subsequently reconstructed the data. The reconstructed confidence interval for the hazard ratio (HR) overlaps with the point estimate reported in the original literature, indicating satisfactory data reconstruction quality (Table S3).

We conducted PH tests and smoothed hazard function tests on the OS and PFS curves for all intervention measures across the six clinical trials. The results showed that most intervention strategies exhibited crossing in the log-cumulative plots, indicating local deviations from the PH assumption. The risk functions were shown in Figure S1–S24. To address the violation of the proportional hazard assumption, we utilized the FP method [33,34]. In the context of generalized linear models, the I^2^ statistic derived from the frequency method is 17%, indicating a limited degree of heterogeneity among studies, thereby supporting the use of a fixed-effects model. Furthermore, we employed survival extrapolation using the Royston-Parmar spline model (RPSM) within a non-linear modelling framework. When dealing with complex risk functions, this approach offers increased flexibility compared to standard parametric models (SPM) [35–37]. Similarly, we employed a Restricted cubic spline model (RCS) to reconstruct the data, for which we configured three distinct node models with 3, 4 and 5 knots, respectively [38,39].

We applied the SPM, RCS and RPSM to the control group data. The SPM encompasses three mathematical methods: Maximum Likelihood Estimation (MLE), Hamiltonian Monte Carlo (HMC), and Integrated Nested Laplace Approximation (INLA) [40,41]. INLA can only fit four distributions, while MLE and HMC can simulate the seven distributions recommended by the National Institute for Health and Care Excellence (NICE) and Canadian Agency for Drugs and Technologies in Health (CADTH)including Exponential, Gamma, Gompertz, Weibull, Loglogistic, Lognormal and Generalized gamma [42,43]. The RPSM included Normal, Hazard and Odds, each with 1, 2 or 3 knots. Ultimately, the Akaike information criterion (AIC) and Bayesian information criterion (BIC) were minimized for the normal 3 knots model under the RPSM [44]. Given the intricate nature of the risk functions across various intervention strategies and the prerequisite of assuming a uniform distribution across all strategies in the FP, we utilized the RPSM normal 3-knots model to extrapolate survival data for all other intervention strategies [33,34]. The supplementary Tables 4 and 5 present the detailed parameters of the extrapolation method used for the survival data of the control group. We utilized the extrapolated survival rates and formulas provided by Mario J. N. M. Ouwens et al. to compute the censoring, event counts, and at-risk populations for each intervention at a specific time point. These computations formed the initial data for the subsequent FP model [45]. We conducted 48 simulations, and the Figures S25–40 and Table S6 present their corresponding AIC, BIC values, hazard ratios (HR), and survival curves. We ultimately chose a first-order FP with P=-2 for the PFS curve and a first-order FP with P=-1 for the OS curve. Among models with smaller AIC values, some exhibited severe tailing issues, while others failed to align with clinical realities. Fitting results are presented in Table 1 and Figures S41 and S42. The HR for each intervention were calculated under the first-order fractional polynomial chosen for this study, as outlined in Equations (1) and (2):

(1) $$\;\text{Ln}\;(h(t))=\beta_{0}+\beta_{1}t^{p},\text{ with }t^{0}=\;\text{log}\;(t),$$

(2) $$\begin{array}{c} \text{Ln}{\left(h(t)\right)}_{1}-\text{Ln}{\left(h(t)\right)}_{2}=(\beta_{10}-\beta_{20})+(\beta_{11}-\beta_{21})*t^{p}\\ =d_{0}+d_{1}t^{p} \end{array}$$

**Table 1.** Parameter of fractional polynomials in frequency.

Progression-free survival curve (first-order polynomial, p=-2)				
	d_0_	d_1_	Distribution	Source
Toripalimab	−0.91 (-1.30, −0.52)	2.76 (-0.08, 5.60)	Uniform	FP
Camrelizumab	−0.69 (-0.95, −0.43)	1.19 (-1.08, 3.46)	Uniform	FP
Pembrolizumab	−0.26 (-0.47, −0.05)	1.14 (-0.62, 2.90)	Uniform	FP
Serplulimab	−0.62 (-0.95, −0.30)	0.85 (-1.50, 3.19)	Uniform	FP
Sintilimab	−0.62 (-0.88, −0.36)	−0.10 (-2.34, 2.16)	Uniform	FP
Tislelizumab	−0.38 (-0.63, −0.14)	−1.34 (-3.36, 0.69)	Uniform	FP
Overall survival curve (first-order polynomial, p=-1)				
	d_0_	d_1_	Distribution	Source
Toripalimab	−1.00 (-1.59, −0.41)	1.71 (-0.86, 4.28)	Uniform	FP
Camrelizumab	−0.41 (-0.82, 0.01)	−0.01 (-2.18, 2.17)	Uniform	FP
Pembrolizumab	−0.34 (-0.60, −0.08)	0.30 (-1.54, 0.94)	Uniform	FP
Serplulimab	−0.73 (-1.13, −0.33)	0.67 (-1.28, 2.62)	Uniform	FP
Sintilimab	−0.45 (-0.82, −0.08)	−0.56 (-2.40, 1.29)	Uniform	FP
Tislelizumab	−0.52 (-0.82, −0.23)	−0.14 (-1.63, 1.36)	Uniform	FP

FP: fractional polynomial.

In the R program, the point estimates and confidence intervals for the parameters d_0_ and d_1_ can be directly obtained.

### Model structure

**Figure 3.**
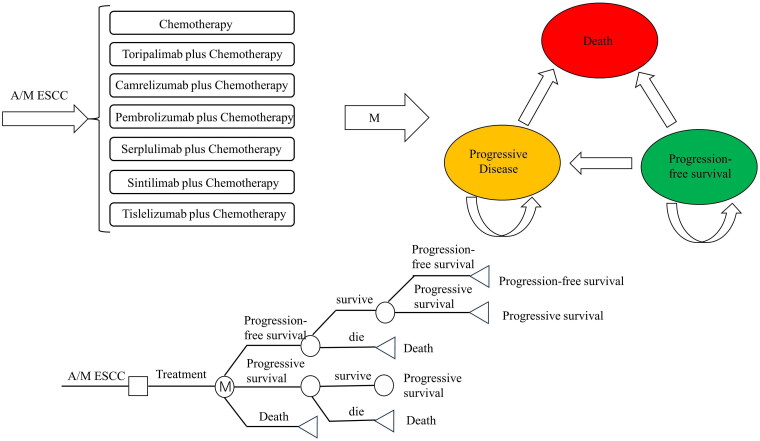
Model structure.

**Figure 4.**
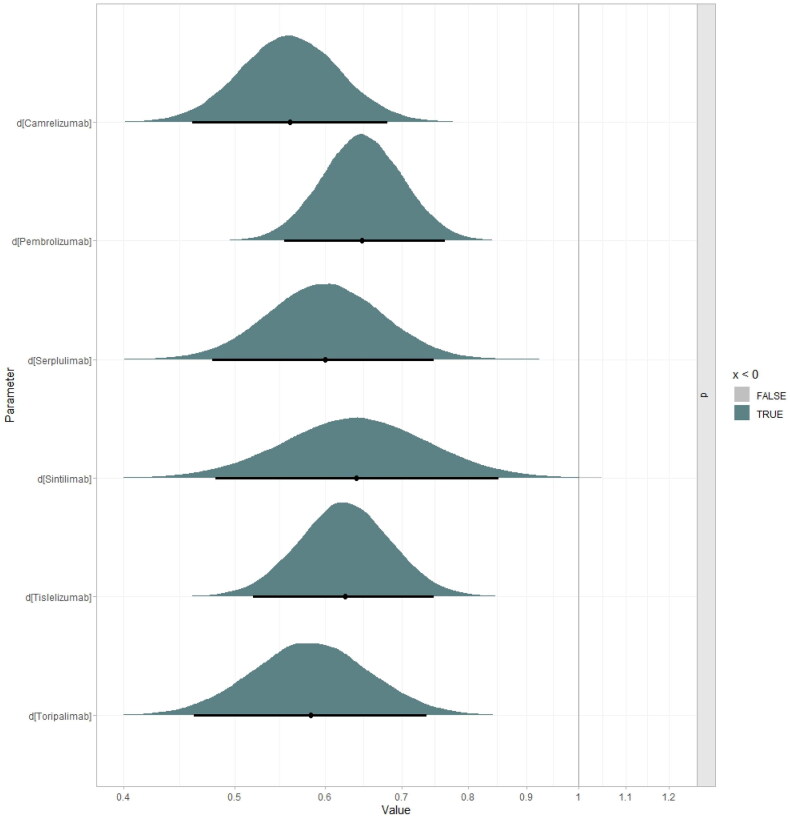
Forest Of PFS.

**Figure 5.**
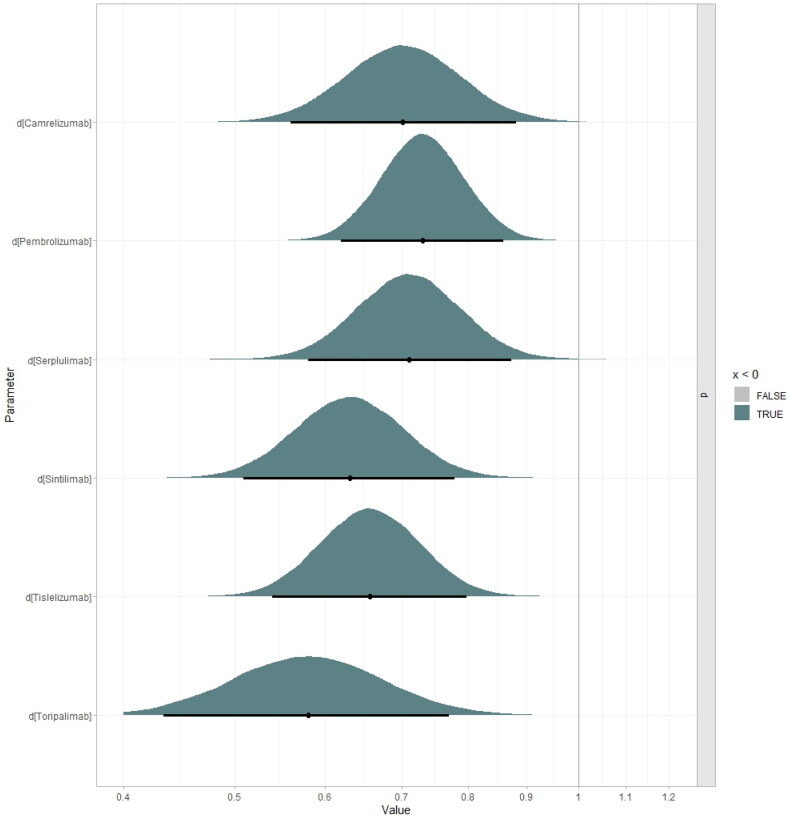
Forest Of OS.

We have followed the Consolidated Health Economic Evaluation Reporting Standards (CHEERS) and developed a partitioned survival model with a 5-year time frame using Excel. This model includes three distinct states: PFS, progressive disease (PD), and death (Figure 3). The corresponding forest plots for PFS and OS are shown in Figures 4 and 5. The dosing schedule for serplulimab is every two weeks, while for the remaining five, it is every three weeks (Table S10) [14–19,46,47]. We have made a half-cycle correction to the model. The currency conversion was based on the average exchange rate of the Chinese Yuan to the US Dollar from January to October 2023, which was 1CNY = 0.14235USD. The willingness-to-pay threshold is set at three times the per capita GDP of China in 2022, amounting to $36,598.19 [48]. In line with the Chinese Pharmacoeconomic Evaluation Guidelines, we have utilized a 5% discount rate [48]. We have adjusted all costs to 2022 prices using the local consumer price index (CPI) and converted them to US dollars. The primary endpoints include total costs, quality-adjusted life years (QALYs), life-years gained (LYs), and the incremental cost-effectiveness ratio (ICER). The study was based on modelling techniques and published literature. Since it did not involve human participants or animals, it did not require ethics approval.

### Costs and utilities

From a healthcare system perspective, we obtained direct medical costs related to the treatment of ESCC through a combination of questionnaire surveys and expert consultations. The costs considered include medication expenses, diagnostic fees, hospitalization costs, adverse event management expenses, and post-line treatment costs. Medication expenses are determined based on actual prices provided by local hospitals, while diagnostic fees are established through the integration of clinical practice and guideline recommendations. When calculating the costs of adverse events, we considered grade 3 or higher AEs with a frequency exceeding 5% across all trials. This encompasses events like anaemia, leukopenia, neutropenia, nausea, vomiting, hypokalaemia and pneumonia (Figure S44 and Table S9) [14–19]. Following the recommendations of clinical trial experts, the model’s adverse event incidence rate is calculated based on the maximum value observed on the PFS curve over time. We assume that all patients in the PD will continue receiving treatment. With regards to post-line treatment, we have referred to the proportion of patients who received post-line treatment in the JUPITER-06 trial [14]. Due to the fact that this proportion is less than 100%, we have incorporated a significant proportion of traditional Chinese medicine (TCM) based on expert consultation and the 2023 Esophageal Cancer Guidelines of the Chinese Society of Clinical Oncology (CSCO). Additionally, we assume that paclitaxel liposome and afatinib are used for chemotherapy and targeted therapy, respectively. All costs are calculated only once within one cycle. Utilities are sourced from published literature, with adverse event utility values assigned negative values [49–52]. Additionally, we assume that patients have a weight of 65 kg and an average body surface area (BSA) of 1.72 m^2^ [26]. Detailed parameter and sources can be found in Table 2.

**Table 2.** Model parameters.

Parameters	Baseline	Range		Distribution	Reference
**Cost**					
**unit-price**		Minimum	Maximum		
Toripalimab (240 mg)	299.07	239.26	299.07	Gamma	Local charge
Camrelizumab (200 mg)	366.78	293.43	366.78	Gamma	Local charge
Pembrolizumab (100 mg)	2550.63	2040.50	2550.63	Gamma	Local charge
Serplulimab (100 mg)	795.45	636.36	795.45	Gamma	Local charge
Sintilimab (200 mg)	153.74	122.99	153.74	Gamma	Local charge
Tislelizumab (200 mg)	392.17	313.74	392.17	Gamma	Local charge
5-fluorouracil (10 ml 250 mg)	4.59	3.67	4.59	Gamma	Local charge
Cisplatin (50 mg)	3.99	3.19	3.99	Gamma	Local charge
Anlotinib (10 mg)	5.21	4.16	5.21	Gamma	Local charge
Paclitaxel liposome (30 mg)	173.52	138.82	173.52	Gamma	Local charge
**Per cycle (21 days)**					
Routine follow-up	78.23	62.58	93.87	Gamma	Local charge
Administration	1.95	1.56	2.34	Gamma	Local charge
Laboratory tests and radiological examinations	379.15	303.32	454.98	Gamma	Local charge
Supportive care	177.87	142.29	213.44	Gamma	Local charge
Hospitalization and daily care	159.72	127.78	191.67	Gamma	Local charge
Traditional oriental herbal	213.53	170.82	256.23	Gamma	Local charge
Anaemia	203.13	162.51	243.76	Gamma	Local charge
Leukopenia	472.07	377.65	566.48	Gamma	Local charge
Neutropenia	472.07	377.65	566.48	Gamma	Local charge
Nausea	20.50	16.40	24.60	Gamma	Local charge
Vomiting	20.50	16.40	24.60	Gamma	Local charge
Hypokalaemia	17.04	13.63	20.45	Gamma	Local charge
Pneumonia	706.82	565.45	848.18	Gamma	Local charge
**Utility**					
Progression-free survival	0.741	0.593	0.889	Beta	[49]
Progressive disease	0.581	0.465	0.697	Beta	[49]
Anaemia	−0.074	0.037	−0.037	Beta	[50]
Leukopenia	−0.090	−0.11	−0.0597	Beta	[51]
Neutropenia	−0.090	−0.1197	−0.059	Beta	[52]
Nausea	−0.048	−0.12	−0.016	Beta	[53]
Vomiting	−0.048	−0.08	−0.016	Beta	[50]
Hypokalaemia	−0.040	−0.08	−0.03	Beta	[54]
Pneumonia	−0.008	−0.05	−0.0064	Beta	[51]
Body surface area(m^2^)	1.720	−0.0096	2.064	Beta	[26]
Body weight (kg)	65.000	52	78	Gamma	[26]
Discount rate	0.050	0	0.08	Beta	[48]

### Sensitivity analysis

We conducted one-way sensitivity analysis to assess the uncertainty of model input parameters. The numerical variations were set at ±20% around the baseline values. Specifically, the upper limit of drug prices was defined as the baseline value, while the lower limit was set at 80% of the baseline value. The results were presented in tornado diagram format. In the probabilistic sensitivity analysis, we inputted the probability distributions of each parameter into the model and subsequently performed 1000 Monte Carlo simulations. he cost and weight were distributed in gamma, while utility values, BSA, and discount rate followed the beta. As for the proportion of treatment during PD, to ensure the correlation between the proportion parameters, we utilized the Dirichlet distribution recommended in the guidelines, with the distribution graph available in the Figure S43 and Table S8 [53,54]. The results were presented in cost-effectiveness scatter plots and cost-effectiveness acceptability curves.

## Results

### Meta-analysis

The findings from the PFS meta-analysis (Figure 4) demonstrate that, when chemotherapy serves as the reference, all six drug combinations with chemotherapy outperform chemotherapy alone. Specifically, the HR for toripalimab, camrelizumab, pembrolizumab, serplulimab, sintilimab and tislelizumab combined with chemotherapy are 0.58 [95% CI: (0.46–0.74)], 0.56 [95% CI: (0.46–0.68)], 0.65 [95% CI: (0.55–0.76)], 0.60 [95% CI: (0.48–0.75)], 0.64 [95% CI: (0.48–0.85)] and 0.63 [95% CI: (0.52–0.75)], respectively. Similarly, the OS meta-analysis (Figure 5) reveals HR of 0.58 [95% CI: (0.43–0.78)], 0.70 [95% CI: (0.56–0.88)], 0.73 [95% CI: (0.62–0.86)], 0.71 [95% CI: (0.58–0.87)], 0.63 [95% CI: (0.51–0.78)] and 0.66 [95% CI: (0.54–0.80)] for the respective combinations. Among the six regimens, camrelizumab plus chemotherapy demonstrated the most significant PFS benefit, while toripalimab plus chemotherapy provided the greatest OS benefit for patients with advanced ESCC.

### Base case analysis

**Figure 6.**
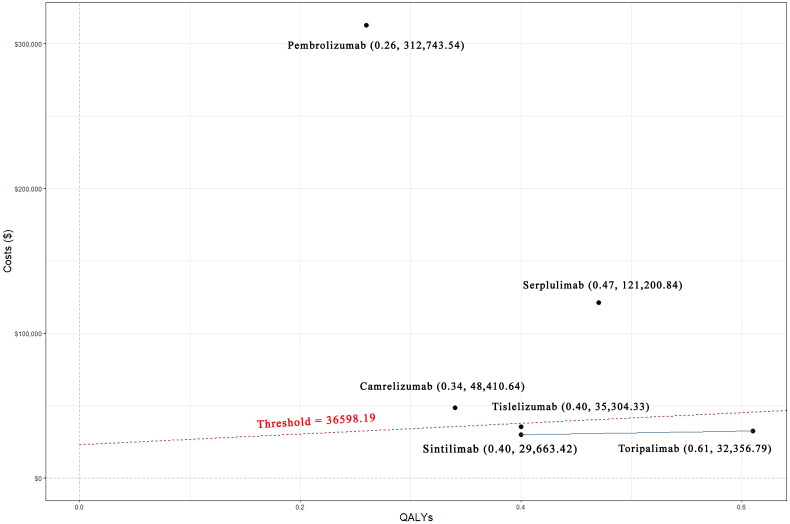
Cost-effective plot.

The final baseline analysis results were presented in and Table 3 and Figure 6. In comparison to chemotherapy alone, ICER for toripalimab plus chemotherapy, camrelizumab plus chemotherapy, pembrolizumab plus chemotherapy, serplulimab plus chemotherapy, sintilimab plus chemotherapy, and tislelizumab plus chemotherapy were $32,356.79/QALY, $48,410.64/QALY, $312,743.54/QALY, $121,200.84/QALY, $29,663.42/QALY and $35,304.33/QALY, respectively. When ranked from lowest to highest ICER, the top three were sintilimab, toripalimab, and tislelizumab, which were also the only three drugs below the WTP threshold. The corresponding LYs were 1.84, 1.41, 1.37, 1.62, 1.52 and 1.56 years, respectively. In terms of LYs ranking, the top three were toripalimab, serplulimab and tislelizumab.

**Table 3.** Results of base-case analysis.

	Cost ($)	QALY	Incremental cost ($)	Incremental QALY	ICER($/QALY)	LYs (years)	Rank (ICER)
Chemotherapy	14,025.37	0.66	–	–	–	0.96	–
Toripalimab plus Chemotherapy	33,743.55	1.27	19,718.18	0.61	32,356.79	1.84	2
Camrelizumab plus Chemotherapy	30,594.67	1.00	16,568.80	0.34	48,410.64	1.41	4
Pembrolizumab plus Chemotherapy	96,591.87	0.92	82,566.50	0.26	312,743.54	1.37	6
Serplulimab plus Chemotherapy	71,012.44	1.13	56,987.07	0.47	121,200.84	1.62	5
Sintilimab Plus Chemotherapy	26,033.52	1.06	12,008.15	0.40	29,633.42	1.52	1
Tislelizumab plus Chemotherapy	28,047.10	1.06	14,021.73	0.40	35,304.33	1.56	3

QALY: quality-adjusted life year; ICER: incremental cost-effectiveness ratio; LYs: life years.

### Sensitivity analysis

**Figure 7.**
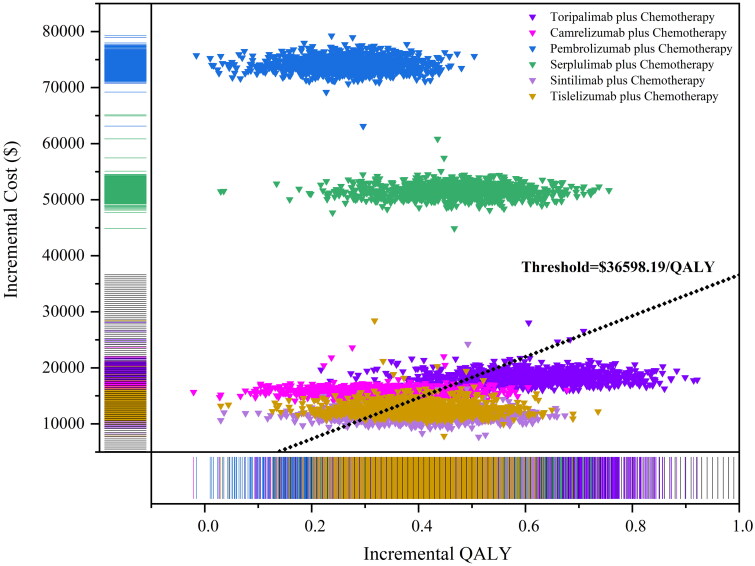
Probabilistic sensitivity analysis in scatter plot (1,000 iterations).

**Figure 8.**
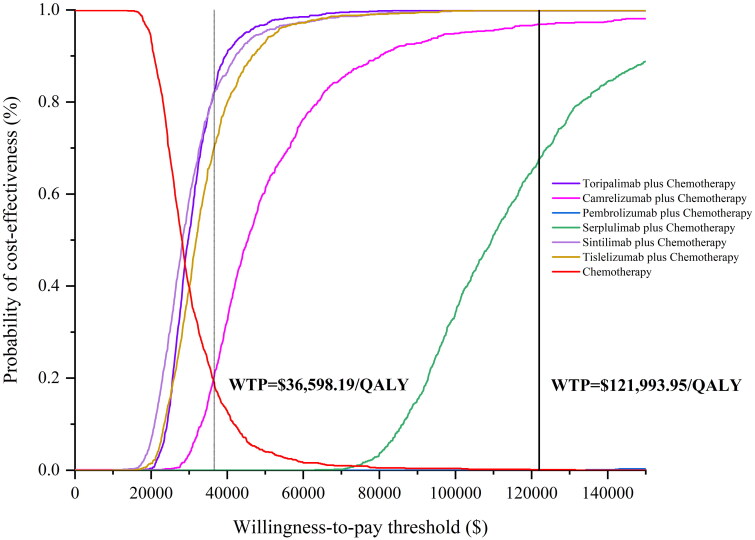
Probabilistic sensitivity analysis: cost-effectiveness acceptability curve (1,000 iterations).

The results of the univariate sensitivity analysis comparing the six interventions with chemotherapy were presented in the supplementary materials Figures S45–S50. The factors that significantly influenced ICER were the utility values of PFS, the parameter of FP model (d_0_, d_1_ of PFS and OS curve), and drug prices. The results of the probabilistic sensitivity analysis were depicted in the Figures 7 and 8, which shows that below the WTP threshold, the probabilities of cost-effectiveness for toripalimab plus chemotherapy, camrelizumab plus chemotherapy, pembrolizumab plus chemotherapy, serplulimab plus chemotherapy, sintilimab plus chemotherapy and tislelizumab plus chemotherapy were 76.8%, 23.1%, 0.0%, 0.0%, 83.5% and 72.7%, respectively. In addition, at a WTP threshold of 10 times GDP per capita, all interventions demonstrated cost-effectiveness.

## Discussion

Our findings indicate that among the six PD-1 inhibitors combined with chemotherapy for first-line treatment of ESCC, sintilimab demonstrated the highest cost-effectiveness, followed by toripalimab, tislelizumab, camrelizumab and serplulimab, while pembrolizumab had the lowest cost-effectiveness. These results were further supported by sensitivity analysis. The tornado diagram highlighted that fluctuations in PD-1 inhibitor prices were among the most influential factors affecting the model’s outcomes. In recent years, rising healthcare costs have intensified global concerns about the economic burden of drug therapies. Beyond clinical efficacy, cost-effectiveness has become a pivotal factor in medical decision-making, especially when choosing treatment options and shaping health insurance policies. Given this, does it suggest that in first-line ESCC treatment, we should predominantly select sintilimab or toripalimab based on cost-effectiveness alone? The answer is more nuanced. Medical decision-making in real-world settings is often far more complex. As providers of healthcare, clinicians possess an inherent information advantage over patients, which can sometimes lead to demand-driven treatments. Furthermore, while cost-effectiveness analysis provides valuable guidance, drug pricing is only one of many factors that influence treatment choices. In practice, patients’ final decisions are rarely based solely on price but are shaped by a broader range of considerations.

According to the China Statistical Yearbook 2024 [55], the per capita GDP for the five regions of China—North, East, South, Central, and West—are estimated at $13,175.06, $16,970.82, $11,043.92, $9,961.74/QALY and $9,792.23, respectively [55]. The corresponding three-fold per capita GDP thresholds are $39,525.19/QALY, $50,912.47/QALY, $33,131.75/QALY, $29,885.22/QALY and $29,376.68/QALY. Based on the findings of this study, the combination therapies involving Toripalimab, Sintilimab and Tislelizumab with chemotherapy may demonstrate cost-effectiveness in the North, East, and South regions. In contrast, for the Central and West regions, the economically viable options are likely limited to Sintilimab combined with chemotherapy and Tislelizumab combined with chemotherapy. Conversely, the combination therapies involving Camrelizumab, Pembrolizumab, and Serplulimab with chemotherapy may not be cost-effective for advanced ESCC patients across all five regions.

Factors such as insurance coverage, patient out-of-pocket expenses, and drug supply chains also play a crucial role in influencing medication choices. From a national healthcare and social security perspective, the high costs associated with PD-1 inhibitor combination therapies place a substantial financial burden on both patients and society. To mitigate this issue, the Chinese government has actively implemented health insurance policies aimed at reducing the prices of PD-1 inhibitors and increasing their accessibility. One of the most prominent strategies in this regard is the drug price negotiation mechanism. The ‘volume-for-price’ strategy is a key component of China’s drug price negotiation process, whereby the National Healthcare Security Administration (NHSA) negotiates with pharmaceutical companies to significantly lower drug prices in exchange for inclusion in the National Reimbursement Drug List (NRDL). This approach enhances drug accessibility while alleviating financial strain on patients. By offering substantial price reductions, pharmaceutical companies gain access to the reimbursement list, which, in turn, expands the market share and usage of their products. After being incorporated into the national health insurance system, the sales of these drugs increase significantly, partially compensating for the revenue loss incurred from price cuts. Since the 2017 reform of China’s National Healthcare Insurance (NHIC), drug price negotiations have become a prerequisite for inclusion in the NRDL. Once drugs are included in the NRDL following national price negotiations, provincial health systems are required to update their Provincial Reimbursement Drug Lists (PRDL) to ensure these drugs are made available at the provincial level. With the continued progress of price negotiations, more cost-effective immunotherapy agents are expected to be added to the NRDL at reduced prices [56]. Public hospitals procure these medications at the negotiated national prices, thereby ensuring that drug procurement remains within controlled price limits. This policy has effectively reduced per-unit procurement costs and improved both the affordability and accessibility of expensive treatments, such as PD-1 inhibitors. In 2019, the first round of price negotiations resulted in an average price reduction of 60.7% for 71 newly negotiated drugs, while 27 drugs that underwent re-negotiation saw an average reduction of 26.4% [57]. These drugs covered 11 therapeutic categories and were successfully incorporated into the NRDL [57]. Notably, the price reductions for new cancer drugs ranged between 34% and 65% [58]. The average accessibility of cancer therapies in China increased from 27.44% to 47.33% [58]. It is crucial to highlight that China’s drug price negotiation policy differs from earlier efforts, which primarily aimed to enhance drug accessibility through health insurance coverage alone. Previously, a patient’s insurance status significantly impacted their ability to access innovative cancer therapies. Under the Urban Employee Basic Medical Insurance and Urban Resident Basic Medical Insurance programs, insured cancer patients benefited from higher reimbursement rates, which eased the financial burden of their treatments. However, uninsured patients faced the full cost of expensive cancer medications, making these innovative therapies unattainable for many. The drug price negotiation policy not only benefits insured patients but also extends its advantages to uninsured individuals by directly lowering drug prices, allowing them access to these innovative cancer treatments at more affordable rates. This approach has substantially improved drug accessibility, enhancing survival rates and quality of life for insured patients while offering cost-effective therapeutic options for uninsured patients. Ultimately, this policy has had a positive impact on the overall health and survival outcomes of the broader patient population.

Currently, while drug price negotiation policies have significantly reduced the prices of PD-1 inhibitors and facilitated the broader use of these high-cost medications, the potential for clinical overuse has emerged as a concern. This may result in an overall increase in healthcare expenditures and place additional pressure on drug supply chains and existing healthcare infrastructure. Particularly for lower-priced domestic PD-1 inhibitors, increased accessibility and higher reimbursement rates have led to reports of off-label use and overtreatment in certain regions. This not only increases the financial burden on health insurance funds but may also expose patients to unnecessary treatments, heightening the risk of adverse drug reactions. Furthermore, large-scale use of low-cost PD-1 inhibitors may strain the drug supply chain, potentially compromising quality control and the stability of long-term supply. As a result, preventing misuse and ensuring the appropriate use of these therapies has become a critical challenge for healthcare regulation. In the first-line treatment of ESCC, toripalimab, camrelizumab, pembrolizumab, serplulimab, sintilimab and tislelizumab, when combined with chemotherapy, have all demonstrated significant clinical benefits, notably improving OS and PFS. The differences in efficacy between domestic and imported PD-1 inhibitors are relatively minor. Regarding safety, these PD-1 inhibitors have comparable profiles, with most adverse events being manageable through appropriate supportive care, although specific side effects may vary between drugs. It is important to recognize that the efficacy of PD-1 inhibitors varies across specific patient subgroups. Pembrolizumab and sintilimab have shown more pronounced survival benefits in patients with high PD-L1 expression (CPS ≥ 10), with pembrolizumab also demonstrating efficacy in patients with oesophageal adenocarcinoma. In contrast, camrelizumab, toripalimab, serplulimab and tislelizumab have shown efficacy that is independent of PD-L1 expression, with positive outcomes observed in ESCC patients across all PD-L1 expression levels. Notably, tislelizumab has demonstrated greater survival benefits in patients with low or negative PD-L1 expression. These variations among the drugs allow clinicians to tailor individualized treatment plans based on the specific conditions of the patient. Although clinical trials have shown similar efficacy across these agents, in real-world clinical practice, treatment decisions must be personalized according to the patient’s overall health status, response to therapy, comorbidities, and other relevant factors. In some cases, a particular drug may offer slight advantages for specific patients, and clinicians must make treatment choices accordingly. Ensuring the rational allocation and sustainable use of healthcare resources is critical. It is also essential to acknowledge that no health insurance policy can indefinitely continue to reduce the costs of PD-1 inhibitors. In China’s diverse healthcare landscape, the effectiveness of policy implementation may vary by region and income level. Despite the growing efforts in drug price negotiations, significant price reductions in certain areas may paradoxically result in some populations not fully benefiting from these policies.

Our study has several limitations. Firstly, we did not consider the statistical differences (*p* = 0.008) in the ECOG PS baseline among patients in six trials. The ECOG PS presents significant limitations as a unidimensional measure of functional capacity. Primarily assessed by physicians, its subjective nature introduces potential bias, and it inadequately addresses critical factors such as multi-morbidity, frailty, and cognitive function. Furthermore, the PS is often documented only once, disregarding the dynamic nature of a patient’s physical condition over time [59,60]. Secondly, in addition to the frequentist-based FP model, we also fitted an FP model within a Bayesian framework (Table S7). The parameters of the hazard function derived from the Bayesian approach were nearly identical to those obtained from the frequentist method. This finding aligns with the results reported by Jansen JP et al. and Wiksten A et al. Consequently, we did not incorporate the FP parameters derived from the Bayesian framework into CUA model [33,34]. Third, we referred to clinical trials to determine the therapy proportion for PD patients. We also incorporated TCM treatment and provided details on the drugs and treatment costs. However, it is important to note that actual treatment practices may vary significantly. Nevertheless, the robustness of the baseline results has been confirmed through one-way sensitivity analysis, which accounts for changes in the proportion and cost of PD stage treatment. Fourth, from a healthcare perspective, we solely incorporated direct medical expenses and examined the cost-effectiveness of the six medications. If we take into account direct non-medical costs and indirect costs, the outcomes might vary [61]. Fifth, because there is a lack of direct comparison clinical trials and it is challenging to obtain data from individual clinical trials, our research findings need to be further validated through matched adjusted indirect comparisons [62,63]. Finally, should we acquire extensive real-world data on the efficacy of six therapeutic agents in the treatment of advanced ESCC in the future, we intend to perform data cleansing, propensity score matching, and subsequent survival data modelling. This will enable a comparative analysis with the extrapolated findings of the current study, thereby facilitating an assessment of the applicability of our results.

## Conclusion

Our study demonstrates that sintilimab combined with chemotherapy is the most cost-effective first-line treatment for advanced ESCC in China, followed by toripalimab and tislelizumab, all of which fall below the WTP threshold. Although all PD-1 inhibitors showed clinical benefits in improving overall survival and progression-free survival, pembrolizumab and serplulimab were less cost-effective due to their higher prices. These findings highlight the importance of balancing clinical efficacy and cost-effectiveness in treatment selection and healthcare policy development for ESCC in China.

## Supplementary Material

Supplemental Material

## Data Availability

The data generated during this study are available from the corresponding author on reasonable request.
