# Supplementary material for: Cost-effectiveness of PD-1 inhibitors combined with chemotherapy for first-line treatment of oesophageal squamous cell carcinoma in China: a comprehensive analysis
Source: Ann Med. 2025 Mar 25;57(1):2482019. doi: 10.1080/07853890.2025.2482019 (PMC11938309; doi:10.1080/07853890.2025.2482019)
Supplement: Supplemental Material [file IANN_A_2482019_SM1981.zip › suppl_data/Suppl_Figures caption.docx]

Supplementary.

Figure Legends

Figure S1. Log-cumulative plot of PFS in JUPITER-06.

Figure S2. Smoothed hazard functions of PFS in JUPITER-06.

Figure S3. Log-cumulative plot of OS in JUPITER-06

Figure S4. Smoothed hazard functions of OS in JUPITER-06

Figure S5. Log-cumulative plot of PFS in ESCORT-1s

Figure S6 Smoothed hazard functions of PFS in ESCORT-1^st^

Figure S7. Log-cumulative plot of OS in ESCORT-1st.

Figure S8. Smoothed hazard functions of OS in ESCORT-1st.

Figure S9. Log-cumulative plot of PFS in KEYNOTE-590.

Figure S10. Smoothed hazard functions of PFS in KEYNOTE-590.

Figure S11. Log-cumulative plot of OS in KEYNOTE-590.

Figure S12. Smoothed hazard functions of OS in KEYNOTE-590.

Figure S13. Log-cumulative plot of PFS in ASTRUM-007.

Figure S14. Smoothed hazard functions of PFS in ASTRUM-007.

Figure S15. Log-cumulative plot of OS in ASTRUM-007.

Figure S16. Smoothed hazard functions of OS in ASTRUM-007.

Figure S17. Log-cumulative plot of PFS in ORIENT-15.

Figure S18. Smoothed hazard functions plot of PFS in ORIENT-15.

Figure S19. Log-cumulative plot of OS in ORIENT-15.

Figure S20. Smoothed hazard functions of OS in ORIENT-15.

Figure S21. Log-cumulative plot of PFS in RATIONALE-306.

Figure S22. Smoothed hazard functions of PFS in RATIONALE-306.

Figure S23. Log-cumulative plot of OS in RATIONALE-306.

Figure S24. Smoothed hazard functions of OS in RATIONALE-306.

Figure S25. Hazard ratio of PFS (1-12).

Figure S26. Hazard ratio of PFS (13-24).

Figure S27. Hazard ratio of PFS (25-36).

Figure S28. Hazard ratio of PFS (37-48).

Figure S29. Survival curve of PFS (1-12).

Figure S30. Survival curve of PFS (13-24).

Figure S31. Survival curve of PFS (25-36).

Figure S32. Survival curve of PFS (37-48).

Figure S33. Hazard ratio of OS (1-12).

Figure S34. Hazard ratio of OS (13-24).

FigureS35. Hazard ratio of OS (25-36).

FigureS36. Hazard ratio of OS (37-48).

FigureS37. Survival curve of OS (1-12).

FigureS38. Survival curve of OS (13-24).

FigureS39. Survival curve of OS (25-36).

FigureS40. Survival curve of OS (37-48).

FigureS41. Progression-free survival curve with first order fractional polynomials (P=-2).

FigureS42. Overall survival curve with first order fractional polynomials (P=-1).

FigureS43. Proportion of subsequent anti-cancer therapy with Dirichlet distribution.

FigureS44. Common adverse events in 6 trials.

FigureS45. Tornado diagram of toripalimab plus chemotherapy vs. chemotherapy.

FigureS46. Tornado diagram camrelizumab plus chemotherapy vs. chemotherapy.

FigureS47. Tornado diagram pembrolizumab plus chemotherapy vs. chemotherapy.

FigureS48. Tornado diagram of serplulimab plus chemotherapy vs. chemotherapy.

FigureS49. Tornado diagram of sintilimab plus chemotherapy vs. chemotherapy.

FigureS50. Tornado diagram of tislelizumab plus chemotherapy vs. chemotherapy.
