# Supplementary material for: Cost-effectiveness of PD-1 inhibitors combined with chemotherapy for first-line treatment of oesophageal squamous cell carcinoma in China: a comprehensive analysis
Source: Ann Med. 2025 Mar 25;57(1):2482019. doi: 10.1080/07853890.2025.2482019 (PMC11938309; doi:10.1080/07853890.2025.2482019)
Supplement: Supplemental Material [file IANN_A_2482019_SM1981.zip › suppl_data/Table S1. Effect information of 6 trials.docx]

**Table S1. Effect information of 6 trials**

| Trial | Group | Medications | Sample size | HR(PFS) | HR(OS) | Median PFS (month) | Median OS (month) | Objective response rate | Patients with grade 3 or higher AEs, (%) |
| --- | --- | --- | --- | --- | --- | --- | --- | --- | --- |
| JUPITER-06 | Intervention | Toripalimab plus Paclitaxel and Cisplatin | 257 | 0.58 (0.46-0.74) | 0.58 (0.43-0.78) | 5.7 | 17.0 | 69.3 | 73.2 |
|  | Control | Paclitaxel and Cisplatin | 257 |  |  | 5.5 | 11.0 | 52.1 | 70.0 |
| ESCORT-1st | Intervention | Camrelizumab plus Paclitaxel and Cisplatin | 298 | 0.56 (0.46-0.68) | 0.7 (0.56-0.88) | 6.9 | 15.3 | 72.1 | 63.4 |
|  | Control | Paclitaxel and Cisplatin | 298 |  |  | 5.6 | 12.0 | 62.1 | 67.7 |
| KEYNOTE-590 | Intervention | Pembrolizumab plus 5-Fluorouracil and Cisplatin | 370 | 0.65(0.55-0.76) | 0.73(0.62-0.86) | 6.3 | 12.4 | 45.0 | 86.0 |
|  | Control | 5-Fluorouracil and Cisplatin | 370 |  |  | 5.8 | 9.8 | 29.3 | 83.0 |
| ASTRUM-007 | Intervention | Serplulimab plus 5-Fluorouracil and Cisplatin | 368 | 0.6(0.45-0.75) | 0.68(0.53-0.87) | 5.8 | 15.3 | 57.6 | 64.0 |
|  | Control | 5-Fluorouracil and Cisplatin | 183 |  |  | 5.3 | 11.8 | 42.1 | 59.0 |
| ORIENT-15 | Intervention | Sintilimab plus 5-Fluorouracil and Cisplatin | 327 | 0.56(0.46-0.68) | 0.63(0.51-0.78) | 7.2 | 16.7 | 66.0 | 60.0 |
|  | Control | 5-Fluorouracil and Cisplatin | 332 |  |  | 5.7 | 12.5 | 49.0 | 55.0 |
| RATIONALE-306 | Intervention | Tislelizumab plus 5-Fluorouracil and Platinum | 326 | 0.62(0.52-0.75) | 0.66(0.54-0.80) | 7.3 | 17.2 | 63.0 | 66.0 |
|  | Control | 5-Fluorouracil and Platinum | 323 |  |  | 5.6 | 10.6 | 42.0 | 65.0 |

HR: hazard ratio; PFS: progression-free survival; OS: overall survival; AEs: adverse events.
