# Supplementary material for: Cost-effectiveness of PD-1 inhibitors combined with chemotherapy for first-line treatment of oesophageal squamous cell carcinoma in China: a comprehensive analysis
Source: Ann Med. 2025 Mar 25;57(1):2482019. doi: 10.1080/07853890.2025.2482019 (PMC11938309; doi:10.1080/07853890.2025.2482019)
Supplement: Supplemental Material [file IANN_A_2482019_SM1981.zip › suppl_data/Table S10. Specific medication modalities in the model.docx]

**Table S10. Specific medication modalities in the model**

| Treatment | Specifications | Administration | Phase | Reference |
| --- | --- | --- | --- | --- |
| Toripalimab | 240mg/bottle | Day 1, 240mg, q3w, iv. | PFS, PD | Wang ZX et al,^14^ 2022 |
| Camrelizumab | 200mg/bottle | Day 1, 200mg, q3w, iv. | PFS, PD | Luo H et al,^15^ 2021 |
| Pembrolizumab | 100mg/bottle | Day 1, 200mg, q3w, iv. | PFS, PD | Sun JM et al,^16^ 2021 |
| Serplulimab | 100mg/bottle | Day 1, 3 mg/kg, q2w, iv. | PFS | Song Y et al,^17^ 2023 |
| Sintilimab | 200mg/bottle | Day 1, 200mg, q3w, iv. | PFS, PD | Lu Z et al,^18^ 2022 |
| Tislelizumab | 200mg/bottle | Day 1, 200mg, q3w, iv. | PFS, PD | Xu J et al,^19^ 2023 |
| 5-fluorouracil | 250mg/10ml | Day 1-5, 800mg/m^2^, q3w, iv. | PFS, PD | Sun JM et al,^16^ 2021 |
| Cisplatin | 50mg/50ml | Day 1, 80 mg/m², q3w, iv. | PFS, PD | Sun JM et al,^16^ 2021 |
| Anlotinib | 10mg*7 pills/box | Day 1-14, qd, po | PD | Li N et al,^44^ 2022 |
| Paclitaxel liposome | 30mg/bottle | Day 1, 135 mg/m^2^, q3w, iv. | PD | Yi Q et al,^45^ 2022 |
| Radiotherapy | NA | NA | PD | Expert consultation |
| TCM | NA | NA | PD | Expert consultation |

PFS: progression-free survival; OS: overall survival; NA: Not applicable, Due to the patient's fluctuating medical condition, we sought advice from experts and have arrived at a rough estimate of the costs; TCM: traditional Chinese medicine.
