# Supplementary material for: Cost-effectiveness of PD-1 inhibitors combined with chemotherapy for first-line treatment of oesophageal squamous cell carcinoma in China: a comprehensive analysis
Source: Ann Med. 2025 Mar 25;57(1):2482019. doi: 10.1080/07853890.2025.2482019 (PMC11938309; doi:10.1080/07853890.2025.2482019)
Supplement: Supplemental Material [file IANN_A_2482019_SM1981.zip › suppl_data/Table S2. Statistical test of common baseline characteristics in 6 trials.docx]

**Table S2. Statistical test of common baseline characteristics in 6 trials**

|  | Gender, (%) | | Age, (%) | | Disease status, (%) | | ECOG, (%) | | PD-L1 status, (%) | |
| --- | --- | --- | --- | --- | --- | --- | --- | --- | --- | --- |
|  | Male | Female | < 65 | ≥ 65 | Metastatic | Locally advanced | 0 | 1 | Score <10 | Score ≥10 |
| Toripalimab | 84.4 | 15.6 | NA | NA | 80.2 | 19.5 | 25.7 | 74.3 | 50.2 | 44.7 |
| Camrelizumab | 87.2 | 12.8 | 67.4 | 32.6 | NA | NA | 23.8 | 76.2 | 63.1 | 34.9 |
| Pembrolizumab | 82.0 | 18.0 | 54.0 | 46.0 | 92.0 | 8.0 | 40.0 | 60.0 | 47.0 | 50.0 |
| Serplulimab | 86.0 | 14.0 | 54.0 | 46.0 | 88.0 | 13.0 | 25.0 | 75.0 | 56.0 | 44.0 |
| Sintilimab | 85.0 | 15.0 | 58.0 | 42.0 | 87.0 | 13.0 | 24.0 | 76.0 | 43.0 | 57.0 |
| Tislelizumab | 87.0 | 13.0 | 54.0 | 46.0 | 86.0 | 14.0 | 33.0 | 67.0 | 36.0 | 46.0 |
| Chemotherapy* | 85.0 | 15.0 | 60.0 | 40.0 | 90.0 | 10.0 | 40.0 | 60.0 | 46.0 | 52.0 |
| *P* value | | | | | | | | | | |
| One-sample t-test | 0.750 | 0.750 | 0.387 | 0.387 | 0.153 | 0.128 | 0.008 | 0.008 | 0.448 | 0.104 |

ECOG: Eastern Cooperative oncology Group; * As a control measure, the baseline characteristics of the patients are used as the reference values for the one-sample t-test. NA: Not applicable, since this item was not provided in the experiment, it is not included in the statistical test.
