# Supplementary material for: Cost-effectiveness of PD-1 inhibitors combined with chemotherapy for first-line treatment of oesophageal squamous cell carcinoma in China: a comprehensive analysis
Source: Ann Med. 2025 Mar 25;57(1):2482019. doi: 10.1080/07853890.2025.2482019 (PMC11938309; doi:10.1080/07853890.2025.2482019)
Supplement: Supplemental Material [file IANN_A_2482019_SM1981.zip › suppl_data/Table S3. Comparison of HR between original and re-construction.docx]

**Table S3. Comparison of HR between original and re-construction**

|  |  | Original | Re-construction |
| --- | --- | --- | --- |
| Toripalimab | PFS | 0.58 (0.46-0.74) | 0.56 (0.45-0.72) |
|  | OS | 0.58 (0.43-0.78) | 0.57 (0.42-0.78) |
| Camrelizumab | PFS | 0.56 (0.46-0.68) | 0.56 (0.46-0.69) |
|  | OS | 0.70 (0.56-0.88) | 0.71 (0.56-0.89) |
| Pembrolizumab | PFS | 0.65 (0.55-0.76) | 0.65 (0.56-0.76) |
|  | OS | 0.73 (0.62-0.86) | 0.71 (0.61-0.84) |
| Serplulimab | PFS | 0.60 (0.45-0.75) | 0.63 (0.51-0.79) |
|  | OS | 0.68 (0.53-0.87) | 0.67 (0.54-0.88) |
| Sintilimab | PFS | 0.56 (0.46-0.68) | 0.55 (0.46-0.67) |
|  | OS | 0.63 (0.51-0.78) | 0.63 (0.51-0.78) |
| Tislelizumab | PFS | 0.62 (0.52-0.75) | 0.61 (0.51-0.74) |
|  | OS | 0.66 (0.54-0.80) | 0.62 (0.53-0.73) |

HR: hazard ratio; PFS: progression-free survival; OS: overall survival.
