# Supplementary material for: Cost-effectiveness of PD-1 inhibitors combined with chemotherapy for first-line treatment of oesophageal squamous cell carcinoma in China: a comprehensive analysis
Source: Ann Med. 2025 Mar 25;57(1):2482019. doi: 10.1080/07853890.2025.2482019 (PMC11938309; doi:10.1080/07853890.2025.2482019)
Supplement: Supplemental Material [file IANN_A_2482019_SM1981.zip › suppl_data/Table S4.The AIC and BIC of different method for control treatment.docx]

**Table S4.The AIC and BIC of different method for control treatment**

|  |  |  | Progression-free survival curve | | Overall survival curve | |
| --- | --- | --- | --- | --- | --- | --- |
|  |  |  | AIC | BIC | AIC | BIC |
| Standard Parameter Model | Maximum likelihood estimation | Exponential | 1968.93 | 1972.86 | 2260.71 | 2264.64 |
|  |  | Gamma | 1915.42 | 1923.28 | 2232.29 | 2240.15 |
|  |  | Gompertz | 1958.89 | 1966.75 | 2255.96 | 2263.82 |
|  |  | Weibull | 1925.89 | 1933.75 | 2237.91 | 2245.77 |
|  |  | Loglogistic | 1911.78 | 1919.64 | 2224.64 | 2232.50 |
|  |  | Lognormal | 1927.94 | 1935.80 | 2238.23 | 2246.09 |
|  |  | Generalized gamma | 1913.38 | 1925.17 | 2230.17 | 2241.96 |
|  | Hamiltonian Monte Carlo | Exponential | 1970.93 | 1978.79 | 2262.71 | 2270.57 |
|  |  | Gamma | 1917.43 | 1929.22 | 2234.29 | 2246.08 |
|  |  | Gompertz | 1961.05 | 1972.84 | 2264.99 | 2276.77 |
|  |  | Weibull | 1927.89 | 1939.68 | 2239.92 | 2251.71 |
|  |  | Loglogistic | 1913.78 | 1925.57 | 2226.64 | 2238.43 |
|  |  | Lognormal | 1929.96 | 1941.75 | 2240.24 | 2252.03 |
|  |  | Generalized gamma | 1915.39 | 1931.11 | 2232.17 | 2247.89 |
|  | Integrated Nested Laplace Approximation | Exponential | 1970.93 | 1978.79 | 2262.71 | 2270.57 |
|  |  | Weibull | 1927.96 | 1939.75 | 2240.06 | 2251.85 |
|  |  | Loglogistic | 1929.96 | 1941.75 | 2240.24 | 2252.03 |
|  |  | Lognormal | 1915.15 | 1926.94 | 2227.44 | 2239.23 |
| Restricted Cubic Spline | - | 3 knots | 1908.10 | 1912.73 | 2226.93 | 2243.17 |
|  |  | 4 knots | 1913.62 | 1915.82 | 2231.87 | 2245.59 |
|  |  | 5 knots | 1919.43 | 1923.76 | 2234.41 | 2247.33 |
| Royston Parmar Spline Model | Hazard | 1 knot | 1914.23 | 1926.90 | 2223.59 | 2242.38 |
|  |  | 2 knots | 1904.39 | 1919.71 | 2224.33 | 2240.04 |
|  |  | 3 knots | 1891.52 | 1911.32 | 2226.20 | 2245.85 |
|  | Odds | 1 knot | 1907.12 | 1923.63 | 2225.60 | 2241.32 |
|  |  | 2 knots | 1907.74 | 1923.54 | 2225.60 | 2241.32 |
|  |  | 3 knots | 1890.25 | 1910.83 | 2226.89 | 2246.54 |
|  | Normal | 1 knot | 1909.73 | 1921.60 | 2227.79 | 2239.57 |
|  |  | 2 knots | 1909.88 | 1924.39 | 2225.89 | 2241.61 |
|  |  | 3 knots | 1890.12 | 1910.50 | 2223.43 | 2240.08 |

AIC: Akaike Information Criterion; BIC: Bayesian Information Criterion
