# Supplementary material for: Cost-effectiveness of PD-1 inhibitors combined with chemotherapy for first-line treatment of oesophageal squamous cell carcinoma in China: a comprehensive analysis
Source: Ann Med. 2025 Mar 25;57(1):2482019. doi: 10.1080/07853890.2025.2482019 (PMC11938309; doi:10.1080/07853890.2025.2482019)
Supplement: Supplemental Material [file IANN_A_2482019_SM1981.zip › suppl_data/Table S5. The parameter of RP spline normal 3 knots model for control treatment.docx]

**Table S5. The parameter of RP spline normal 3 knots model for control treatment**

|  | Estimate | Lower | Upper | Standard error |
| --- | --- | --- | --- | --- |
| Progression-free survival curve | | | | |
| gamma0 | -1.629 | -1.893 | -1.365 | 0.135 |
| gamma1 | 0.78 | 0.411 | 1.15 | 0.189 |
| gamma2 | 0.784 | 0.384 | 1.184 | 0.204 |
| gamma3 | -5.005 | -7.141 | -2.87 | 1.09 |
| gamma4 | 4.45 | 2.577 | 6.323 | 0.956 |
| Overall survival curve | | | | |
| gamma0 | -2.065 | -2.291 | -1.839 | 0.1153 |
| gamma1 | 0.5475 | 0.2686 | 0.8265 | 0.1423 |
| gamma2 | 0.1516 | -0.251 | 0.5542 | 0.2054 |
| gamma3 | -0.7612 | -1.7971 | 0.2747 | 0.5285 |
| gamma4 | 0.7387 | -0.0808 | 1.5582 | 0.4181 |
