# Supplementary material for: Cost-effectiveness of PD-1 inhibitors combined with chemotherapy for first-line treatment of oesophageal squamous cell carcinoma in China: a comprehensive analysis
Source: Ann Med. 2025 Mar 25;57(1):2482019. doi: 10.1080/07853890.2025.2482019 (PMC11938309; doi:10.1080/07853890.2025.2482019)
Supplement: Supplemental Material [file IANN_A_2482019_SM1981.zip › suppl_data/Table S6. The selection of the best model in 48 models.docx]

**Table S6. The selection of the best model in 48 models**

| Model Category | PFS | |  | OS | |  |
| --- | --- | --- | --- | --- | --- | --- |
|  | AIC | BIC |  | AIC | BIC |  |
| ^1^Weibull,p1=0 | 687.04 | 750.74 | a,c | 674.2 | 743.57 | a,c |
| PWE, cutpoint 2 | 678.58 | 742.27 | a,c | 714.99 | 784.36 | c |
| PWE, cutpoints 2 and 10 | 630.13 | 725.68 | a | 702.6 | 806.65 | a,c |
| PWE, cutpoints 2 and 12 | 641.38 | 734.27 | a | 707.22 | 811.27 | a,c |
| First order FP, p1=-2 | 669.51 | 733.21 | best | 679.89 | 749.25 | c |
| First order FP, p1=-1 | 673.64 | 737.33 | c | 665.71 | 735.08 | best |
| First order FP, p1=-0.5 | 679.39 | 743.09 | c | 665.89 | 735.26 | c |
| First order FP, p1=0 | 762.61 | 794.45 | c | 808.97 | 843.66 | c |
| First order FP, p1=0.5 | 695.05 | 758.74 | a | 689.38 | 758.74 | a,c |
| First order FP, p1=1 | 701.95 | 765.65 | a,c | 707.69 | 777.06 | a,c |
| First order FP, p1=2 | 710.43 | 774.13 | a,c | 741.33 | 810.7 | a,b,c |
| ^12^First order FP, p1=3 | 714.37 | 778.07 | a,c | 764.85 | 834.22 | a,b,c |
| Second order FP,p1=-2, p2=-2 | 669.51 | 733.21 | * | 679.89 | 749.25 | c |
| Second order FP,p1=-2, p2=-1 | 622.35 | 717.89 | a | 665.39 | 769.45 | a,b,c |
| Second order FP,p1=-2, p2=-0.5 | 579.84 | 678.04 | a,b | 660.72 | 767.66 | a,b,c |
| Second order FP,p1=-2, p2=0 | 669.51 | 733.21 | * | 679.89 | 749.25 | c |
| Second order FP,p1=-2, p2=0.5 | 604.57 | 700.11 | a | 663.77 | 767.82 | a,b |
| Second order FP,p1=-2, p2=1 | 599.33 | 694.87 | a | 664.17 | 768.22 | a,b |
| Second order FP,p1=-2, p2=2 | 593.69 | 689.23 | a | 666.78 | 770.83 | a,b,c |
| Second order FP,p1=-2, p2=3 | 595.12 | 690.66 | a | 670.93 | 774.98 | a,b,c |
| Second order FP,p1=-1, p2=-1 | 673.64 | 737.33 | c | 665.71 | 735.08 | * |
| Second order FP,p1=-1, p2=-0.5 | 605.06 | 700.6 | a | 660.52 | 764.57 | a,b |
| Second order FP,p1=-1, p2=0 | 673.64 | 737.33 | a | 665.71 | 735.08 | * |
| ^24^Second order FP,p1=-1, p2=0.5 | 592.22 | 687.76 | a | 657.56 | 761.61 | a,b |
| Second order FP,p1=-1, p2=1 | 587.54 | 683.08 | a | 656.9 | 760.95 | a,b |
| Second order FP,p1=-1, p2=2 | 584.45 | 680 | a | 657.68 | 761.73 | a,b |
| Second order FP,p1=-1, p2=3 | 589.24 | 684.78 | a | 660.55 | 764.6 | a,b |
| Second order FP,p1=-0.5, p2=-0.5 | 679.39 | 743.09 | c | 665.89 | 735.26 | a |
| Second order FP,p1=-0.5, p2=0 | 679.39 | 743.09 | c | 665.89 | 735.26 | a |
| Second order FP,p1=-0.5, p2=0.5 | 585.95 | 681.5 | a | 654.77 | 758.82 | a,b |
| Second order FP,p1=-0.5, p2=1 | 582.19 | 677.73 | a | 654.09 | 758.14 | a,b |
| Second order FP,p1=-0.5, p2=2 | 581.84 | 677.38 | a | 655.23 | 759.28 | a,b |
| Second order FP,p1=-0.5, p2=3 | 589.64 | 685.18 | a | 658.66 | 762.71 | a,b |
| Second order FP,p1=0, p2=0 | 762.61 | 794.45 | c | 808.97 | 843.66 | c |
| Second order FP,p1=0, p2=0.5 | 695.05 | 758.74 | c | 689.38 | 758.74 | a,c |
| ^36^Second order FP,p1=0, p2=1 | 701.95 | 765.65 | a,c | 707.69 | 777.06 | a,c |
| Second order FP,p1=0, p2=2 | 710.43 | 774.13 | a,c | 741.33 | 810.7 | a,b,c |
| Second order FP,p1=0, p2=3 | 714.37 | 778.07 | a,c | 764.85 | 834.22 | a,b,c |
| Second order FP,p1=0.5, p2=0.5 | 695.05 | 758.74 | a,c | 689.38 | 758.74 | a,c |
| Second order FP,p1=0.5, p2=1 | 577.83 | 673.38 | a | 653.66 | 757.71 | a,b |
| Second order FP,p1=0.5, p2=2 | 586.61 | 682.15 | a | 659.47 | 763.52 | a,b |
| Second order FP,p1=0.5, p2=3 | 602.92 | 698.47 | a | 667.34 | 771.39 | a,b,c |
| Second order FP,p1=1, p2=1 | 701.95 | 765.65 | a,c | 707.69 | 777.06 | a,c |
| Second order FP,p1=1, p2=2 | 594.95 | 690.5 | a | 665.92 | 769.97 | a,c |
| Second order FP,p1=1, p2=3 | 615.7 | 711.24 | a,b | 676.75 | 780.8 | a,b,c |
| Second order FP,p1=2, p2=2 | 646.32 | 741.86 | a,b | 698.04 | 802.1 | a,b,c |
| Second order FP,p1=2, p2=3 | 646.32 | 741.86 | a,b | 698.04 | 802.1 | a,b,c |
| ^48^Second order FP,p1=3, p2=3 | 714.37 | 778.07 | a,c | 764.85 | 834.22 | a,b,c |

FP: fractional polynomial; PFS: progression-free survival; OS: overall survival; AIC: Akaike Information Criterion; BIC: Bayesian Information Criterion; a: The fitting curve has a fat tail; b: The results is deviated from actuality; c: AIC value is too large; *: Indicates models where AIC and BIC are consistent but do not meet the abc criteria; best: The model exhibits no significant long tail, aligns well with clinical observations, and demonstrates a relatively smaller AIC value. The superscripts 1, 12, 24, 36, and 48 serve as positioning indicators, corresponding to Models 1-12, Models 13-24, Models 25-36, and Models 37-48, respectively, as detailed in Section 6.2.
