# Supplementary material for: Cost-effectiveness of PD-1 inhibitors combined with chemotherapy for first-line treatment of oesophageal squamous cell carcinoma in China: a comprehensive analysis
Source: Ann Med. 2025 Mar 25;57(1):2482019. doi: 10.1080/07853890.2025.2482019 (PMC11938309; doi:10.1080/07853890.2025.2482019)
Supplement: Supplemental Material [file IANN_A_2482019_SM1981.zip › suppl_data/Table S7. Parameter of fractional polynomials in Bayesian framework.docx]

**Table S7. Parameter of fractional polynomials in Bayesian framework**

| Progression-free survival curve (first-order polynomial, p=-2) | | |
| --- | --- | --- |
|  | d_0_ | d_1_ |
| Toripalimab | -0.91 (-1.31- -0.51) | 2.82 (-0.03- 5.67) |
| Camrelizumab | -0.67 (-0.94- -0.40) | 1.18 (-1.05- 3.41) |
| Pembrolizumab | -0.23 (-0.44- -0.02) | 1.16 (-0.61- 2.93) |
| Serplulimab | -0.60 (-0.93- -0.27) | 0.86(-1.48- 3.20) |
| Sintilimab | -0.63 (-0.89- -0.37) | -0.09 (-2.34- 2.16) |
| Tislelizumab | -0.38 (-0.62- -0.14) | -1.35 (-3.38- 0.68) |
| Overall survival curve (first-order polynomial, p=-1) | | |
|  | d_0_ | d_1_ |
| Toripalimab | -0.98 (-1.56- -0.40) | 1.72 (-0.83- 4.27) |
| Camrelizumab | -0.41 (-0.82- 0.03) | -0.02 (-2.19- 2.15） |
| Pembrolizumab | -0.33 (-0.64- -0.02) | 0.31 (-1.52- 2.14) |
| Serplulimab | -0.73 (-1.16- -0.30) | 0.66 (-1.29- 2.61) |
| Sintilimab | -0.44 (-0.83- -0.05) | -0.56 (-2.37- 1.25) |
| Tislelizumab | -0.50 (-0.81- -0.19) | -0.15 (-1.60- 1.30) |
