# Supplementary material for: Cost-effectiveness of PD-1 inhibitors combined with chemotherapy for first-line treatment of oesophageal squamous cell carcinoma in China: a comprehensive analysis
Source: Ann Med. 2025 Mar 25;57(1):2482019. doi: 10.1080/07853890.2025.2482019 (PMC11938309; doi:10.1080/07853890.2025.2482019)
Supplement: Supplemental Material [file IANN_A_2482019_SM1981.zip › suppl_data/Table S8. Detail of subsequent anti-cancer therapy.docx]

**Table S8. Detail of subsequent anti-cancer therapy**

| Therapy | Proportion | Distribution | Reference |
| --- | --- | --- | --- |
| Chemothrapy |  |  |  |
| Paclitaxel liposome | 0.268 | Dirichlet | Wang ZX et al,^14^ 2022 |
| Radiation therapy | 0.144 | Dirichlet | Wang ZX et al,^14^ 2022 |
| Targeting therapy |  |  |  |
| Anlotinib | 0.134 | Dirichlet | Wang ZX et al,^14^ 2022 |
| Anti-PD-1/PD-L1 |  |  |  |
| Camrelizumab | 0.051 | Dirichlet | Wang ZX et al,^14^ 2022 |
| Toripalimab | 0.027 | Dirichlet | Wang ZX et al,^14^ 2022 |
| Sintilimab | 0.023 | Dirichlet | Wang ZX et al,^14^ 2022 |
| Pembrolizumab | 0.006 | Dirichlet | Wang ZX et al,^14^ 2022 |
| Tisleizumab | 0.002 | Dirichlet | Wang ZX et al,^14^ 2022 |
| Traditional oriental herbal | 0.345 | Dirichlet | Expert consultation |
