# Supplementary material for: Cost-effectiveness of PD-1 inhibitors combined with chemotherapy for first-line treatment of oesophageal squamous cell carcinoma in China: a comprehensive analysis
Source: Ann Med. 2025 Mar 25;57(1):2482019. doi: 10.1080/07853890.2025.2482019 (PMC11938309; doi:10.1080/07853890.2025.2482019)
Supplement: Supplemental Material [file IANN_A_2482019_SM1981.zip › suppl_data/Table S9. The incidence of adverse reactions in the model.docx]

**Table S9. The incidence of adverse reactions in the model**

|  | Anemia | Leukopenia | Neutropenia | Nausea | Vomiting | Hypokalaemia | Pneumonia | Reference |
| --- | --- | --- | --- | --- | --- | --- | --- | --- |
| Chemotherapy | 0.15 | 0.05 | 0.16 | 0.06 | 0.05 | 0.05 | 0 | Sun JM et al,^16^ 2021 |
| Toripalimab | 0.109 | 0.202 | 0.424 | 0 | 0.019 | 0.031 | 0.058 | Wang ZX et al,^14^ 2022 |
| Camrelizumab | 0.174 | 0.242 | 0.399 | 0.013 | 0.034 | 0 | 0.03 | Luo H et al,^15^ 2021 |
| Pembrolizumab | 0.12 | 0.09 | 0.14 | 0.07 | 0.06 | 0.05 | 0.02 | Sun JM et al,^16^ 2021 |
| Serplulimab | 0.18 | 0.11 | 0.19 | 0.03 | 0.03 | 0.04 | 0 | Song Y et al,^17^ 2023 |
| Sintilimab | 0.13 | 0.174 | 0.3 | 0.006 | 0.006 | 0.049 | 0.021 | Lu Z et al,^18^ 2022 |
| Tislelizumab | 0.14 | 0.108 | 0.305 | 0.02 | 0.01 | 0.05 | 0 | Xu J et al,^19^ 2023 |
