# Supplementary figures and images for: Cost-effectiveness of PD-1 inhibitors combined with chemotherapy for first-line treatment of oesophageal squamous cell carcinoma in China: a comprehensive analysis
Source: Ann Med. 2025 Mar 25;57(1):2482019. doi: 10.1080/07853890.2025.2482019 (PMC11938309; doi:10.1080/07853890.2025.2482019)

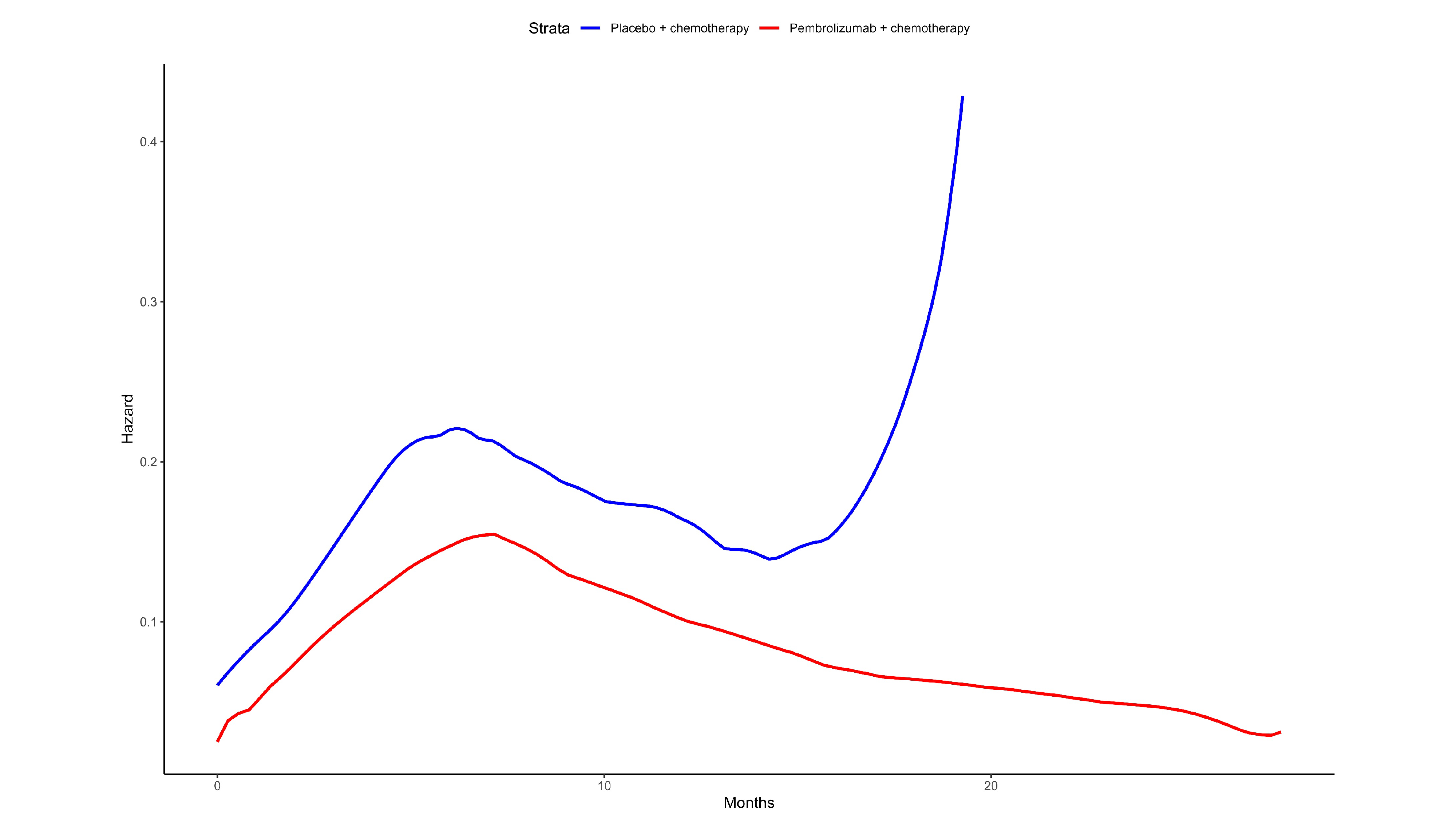

Supplement: Supplemental Material [file IANN_A_2482019_SM1981.zip › suppl_data/Figure S10. Smoothed hazard functions of PFS in KEYNOTE-590.tiff]

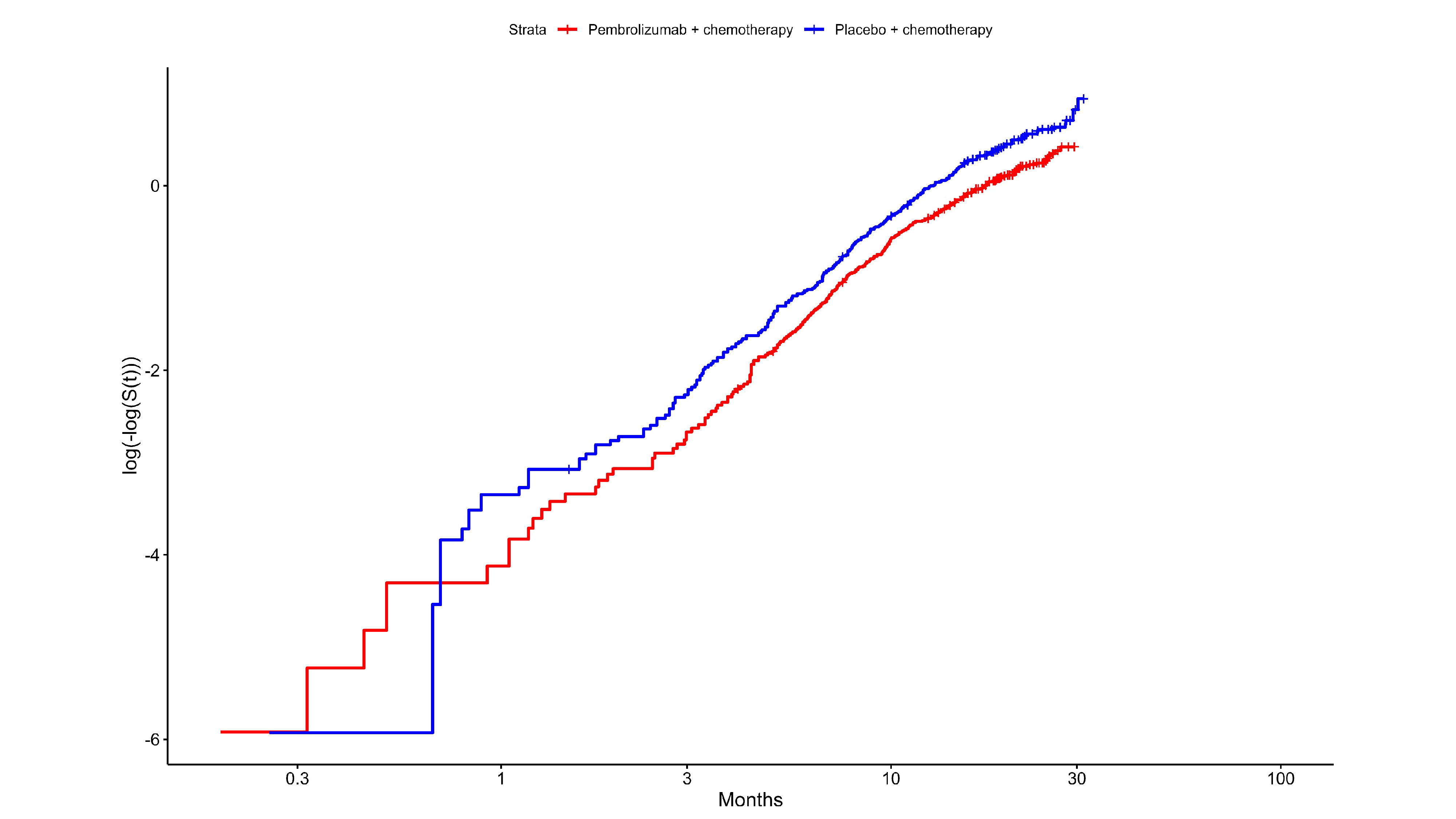

Supplement: Supplemental Material [file IANN_A_2482019_SM1981.zip › suppl_data/Figure S11. Log-cumulative plot of OS in KEYNOTE-590.tiff]

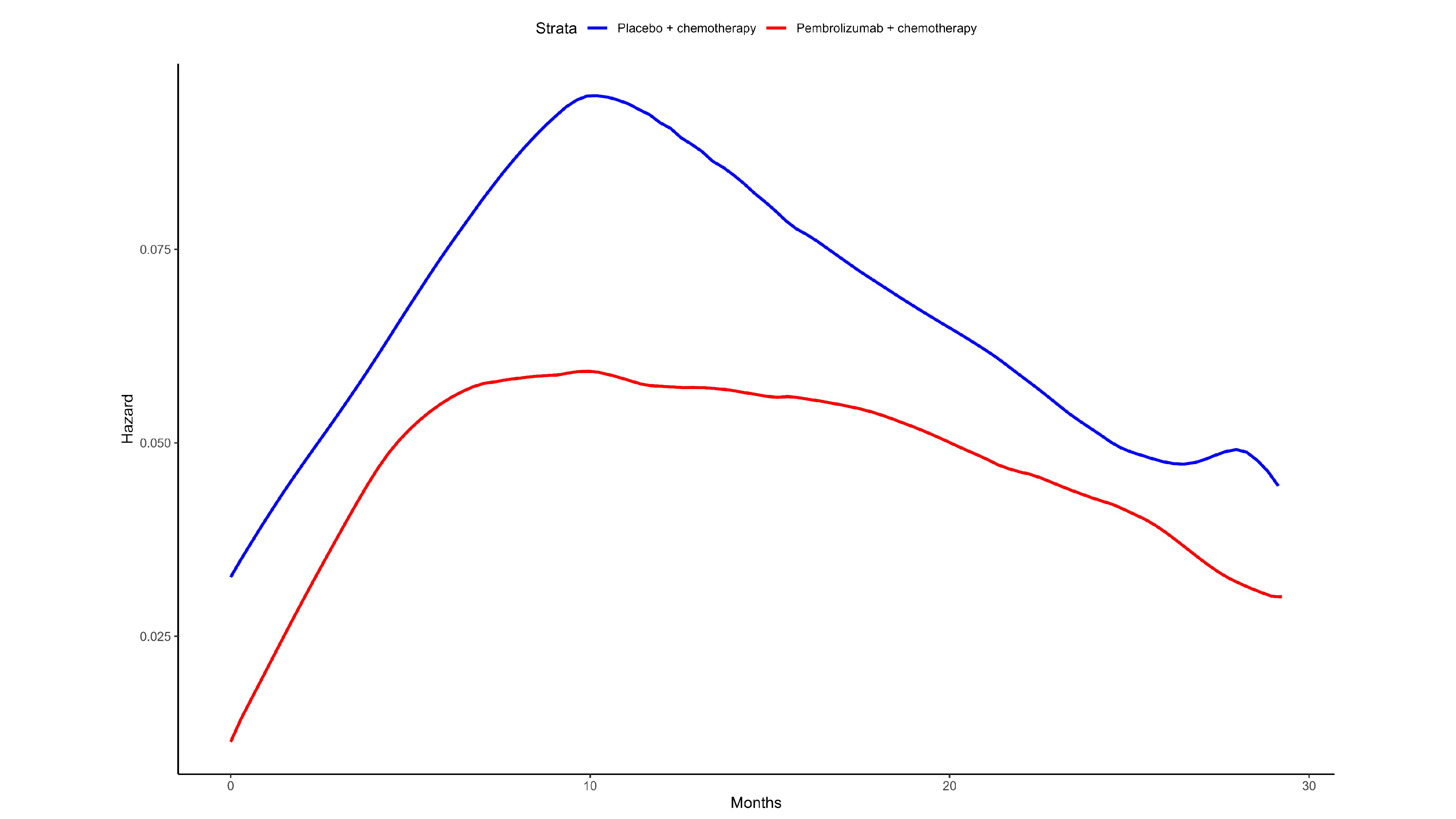

Supplement: Supplemental Material [file IANN_A_2482019_SM1981.zip › suppl_data/Figure S12. Smoothed hazard functions of OS in KEYNOTE-590.tiff]

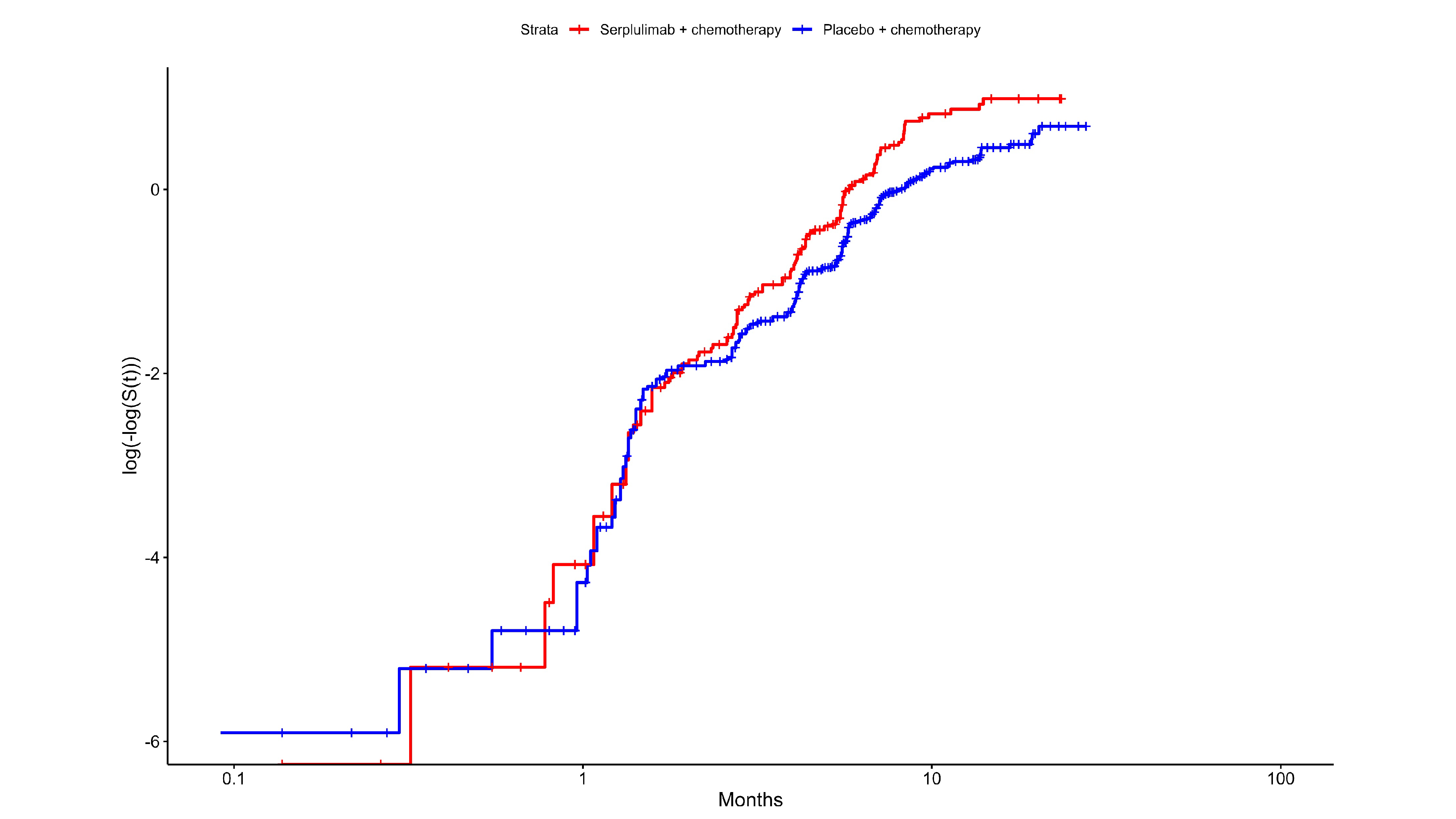

Supplement: Supplemental Material [file IANN_A_2482019_SM1981.zip › suppl_data/Figure S13. Log-cumulative plot of PFS in ASTRUM-007.tiff]

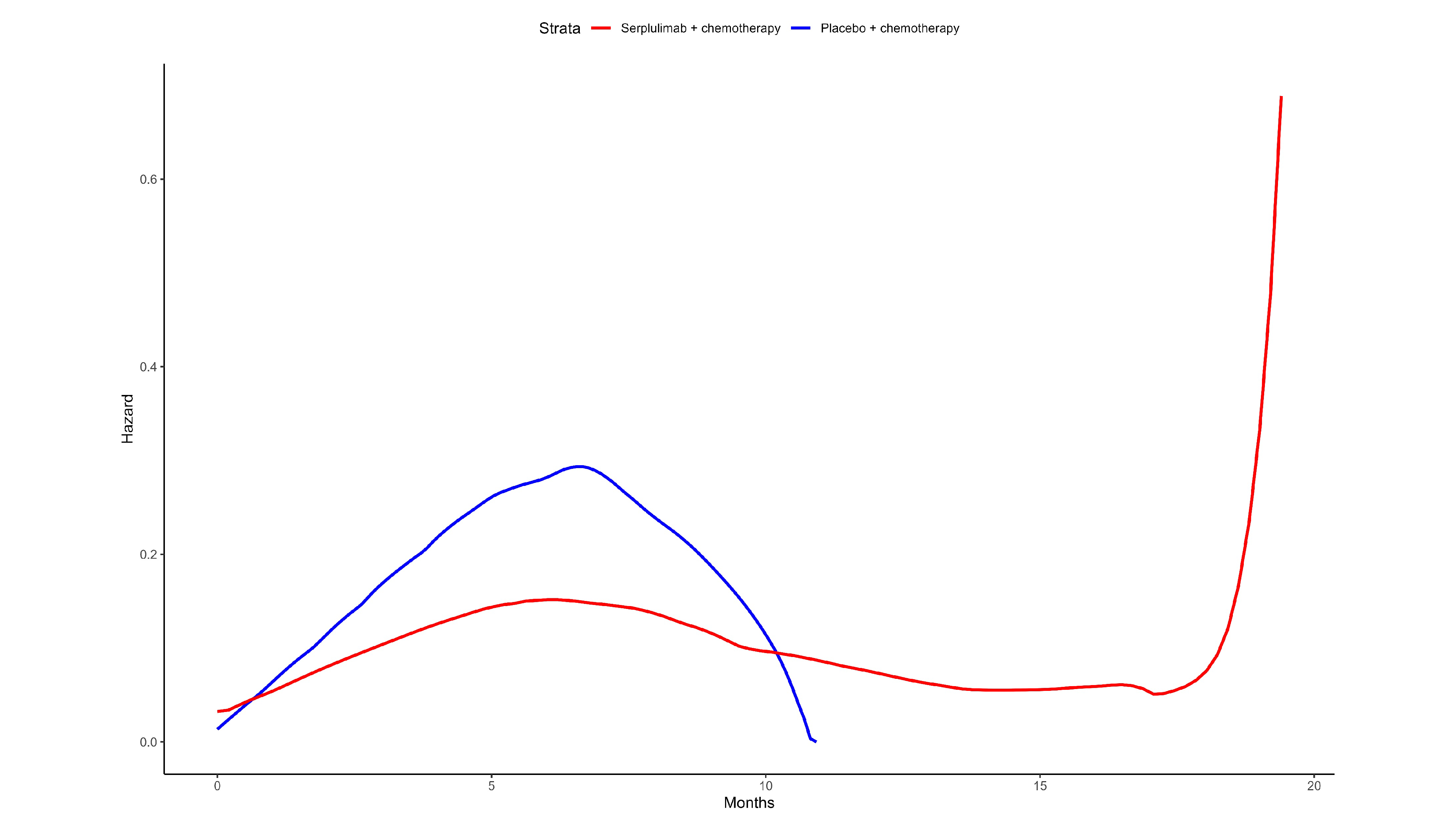

Supplement: Supplemental Material [file IANN_A_2482019_SM1981.zip › suppl_data/Figure S14. Smoothed hazard functions of PFS in ASTRUM-007.tiff]

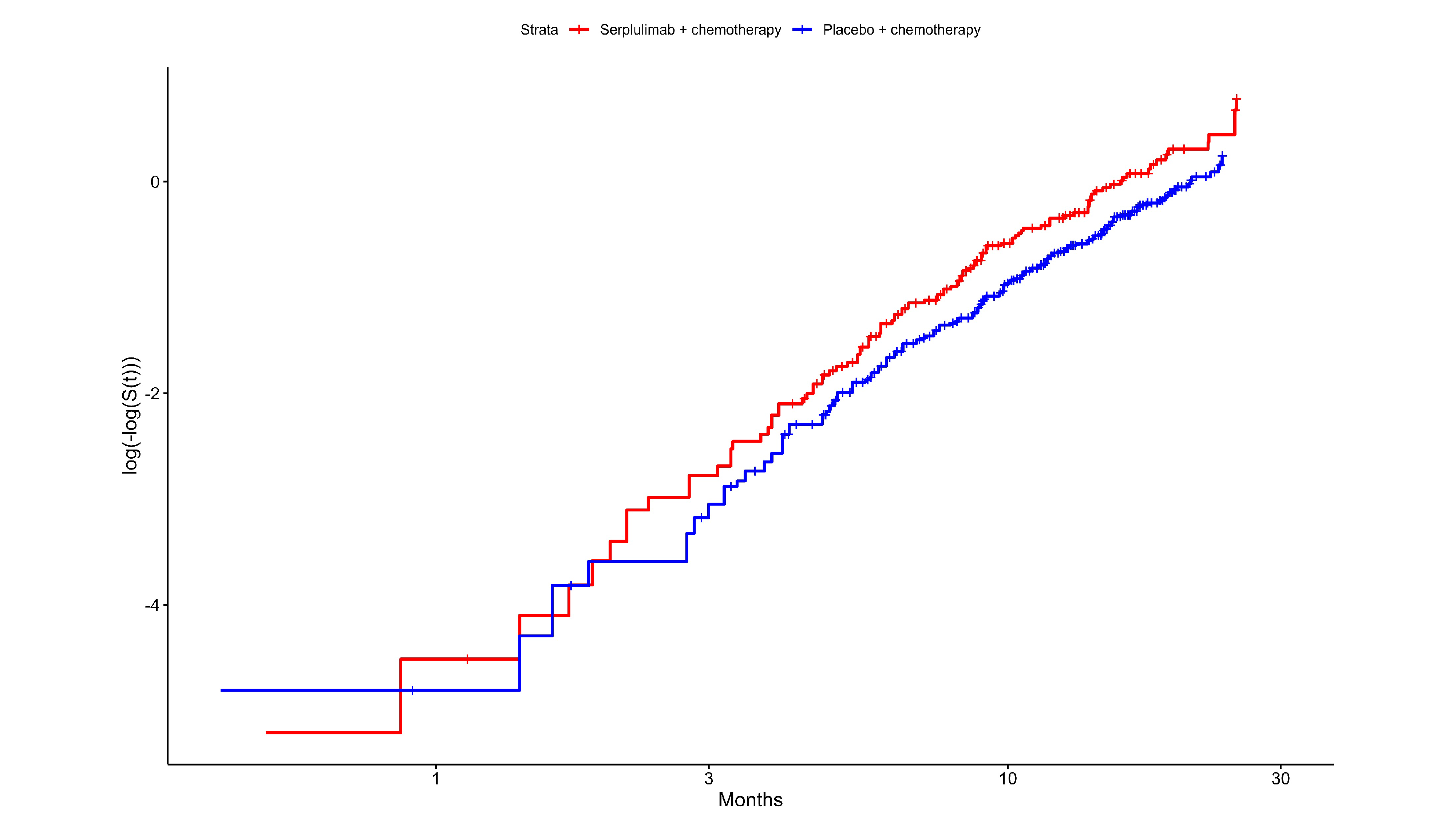

Supplement: Supplemental Material [file IANN_A_2482019_SM1981.zip › suppl_data/Figure S15. Log-cumulative plot of OS in ASTRUM-007.tiff]

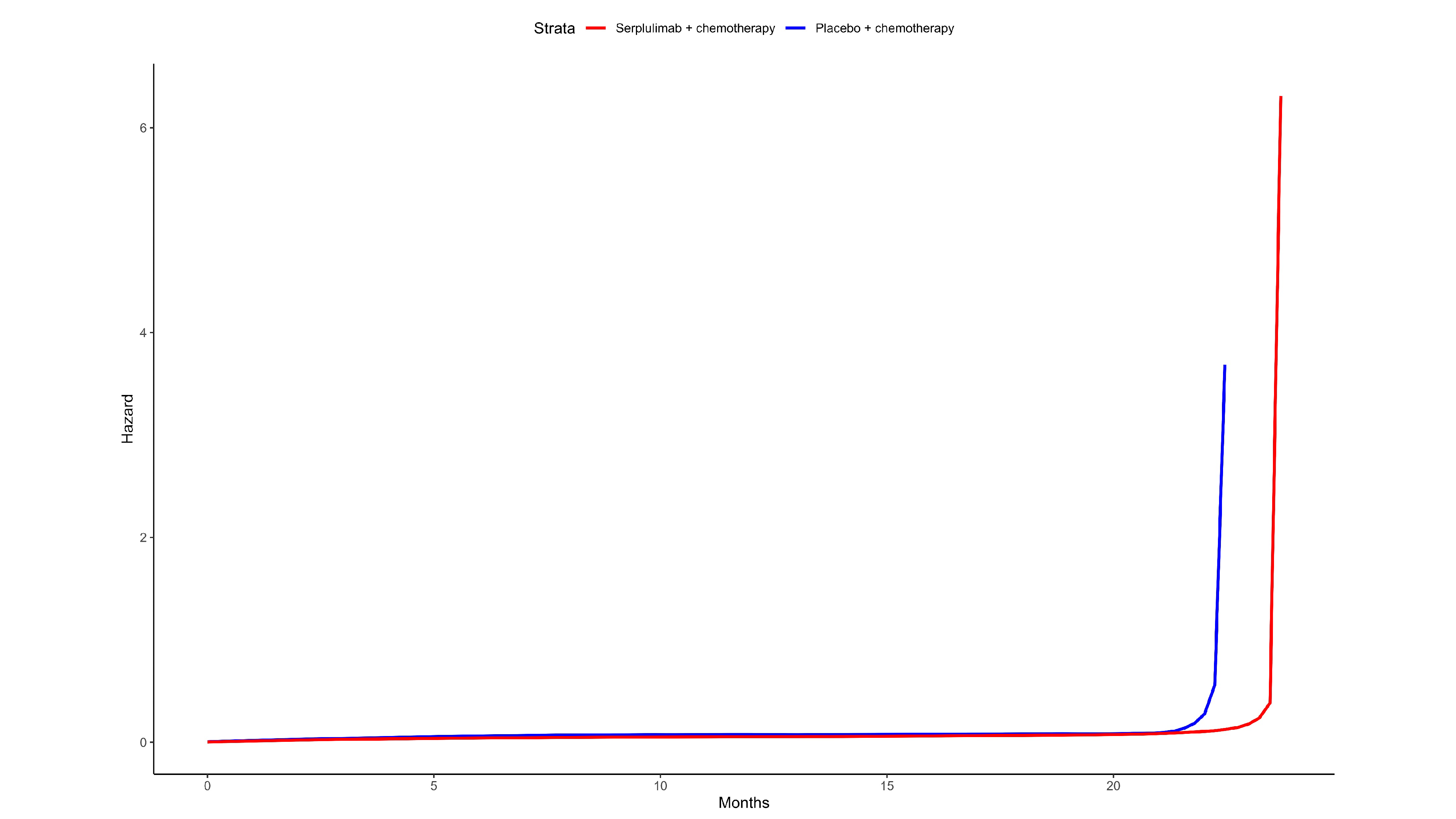

Supplement: Supplemental Material [file IANN_A_2482019_SM1981.zip › suppl_data/Figure S16. Smoothed hazard functions of OS in ASTRUM-007.tiff]

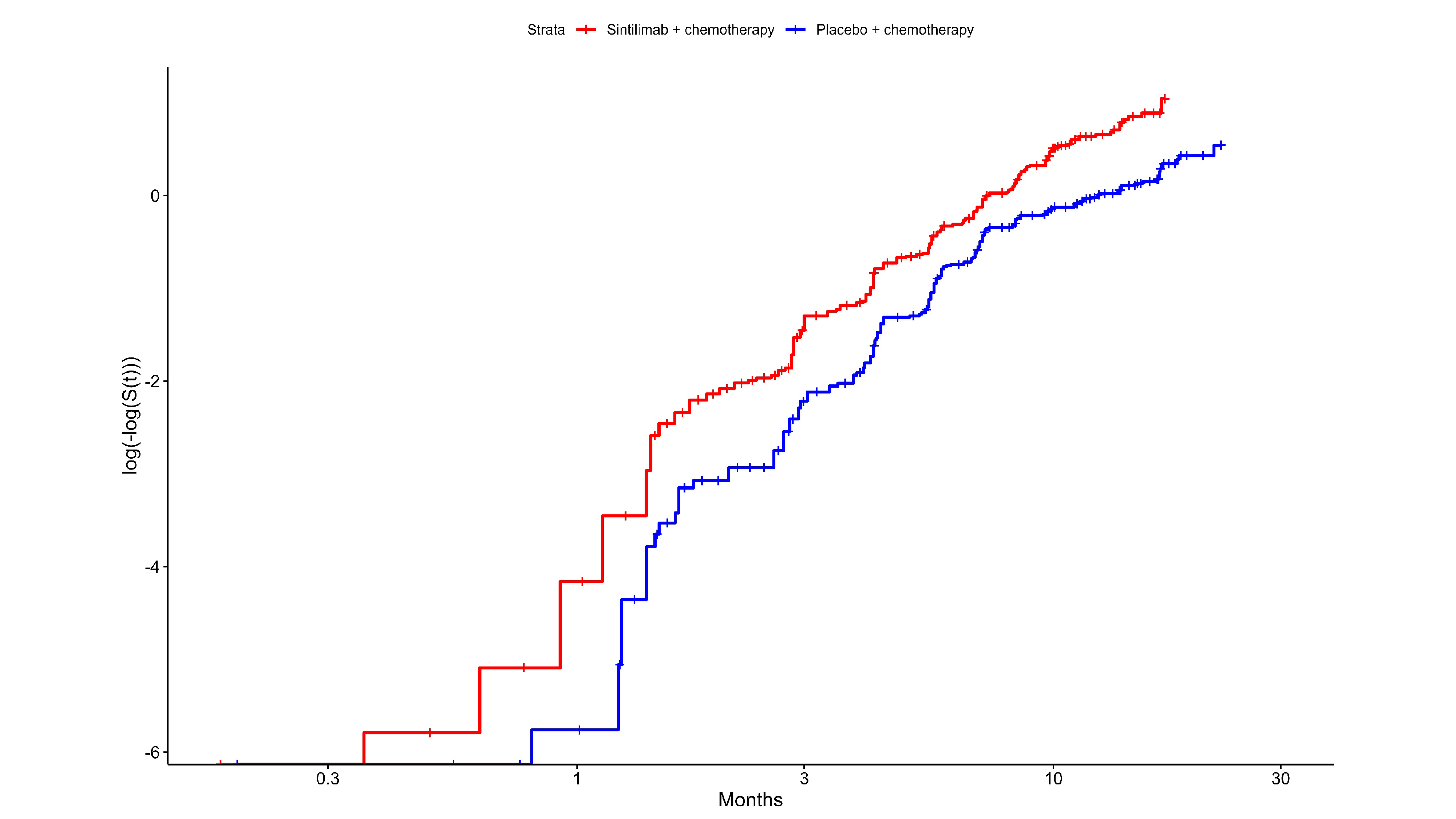

Supplement: Supplemental Material [file IANN_A_2482019_SM1981.zip › suppl_data/Figure S17. Log-cumulative plot of PFS in ORIENT-15.tiff]

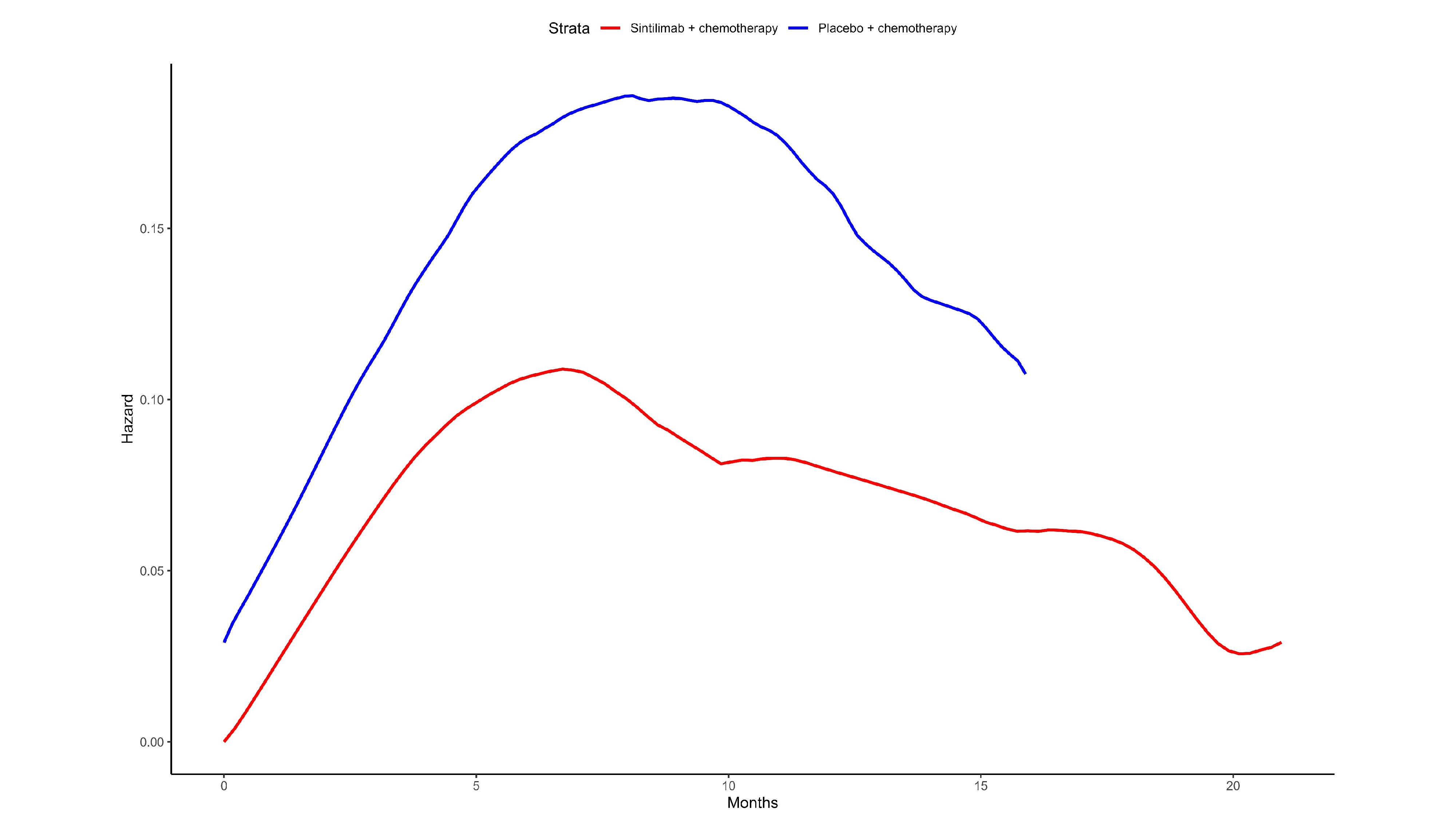

Supplement: Supplemental Material [file IANN_A_2482019_SM1981.zip › suppl_data/Figure S18. Smoothed hazard functions plot of PFS in ORIENT-15.tiff]

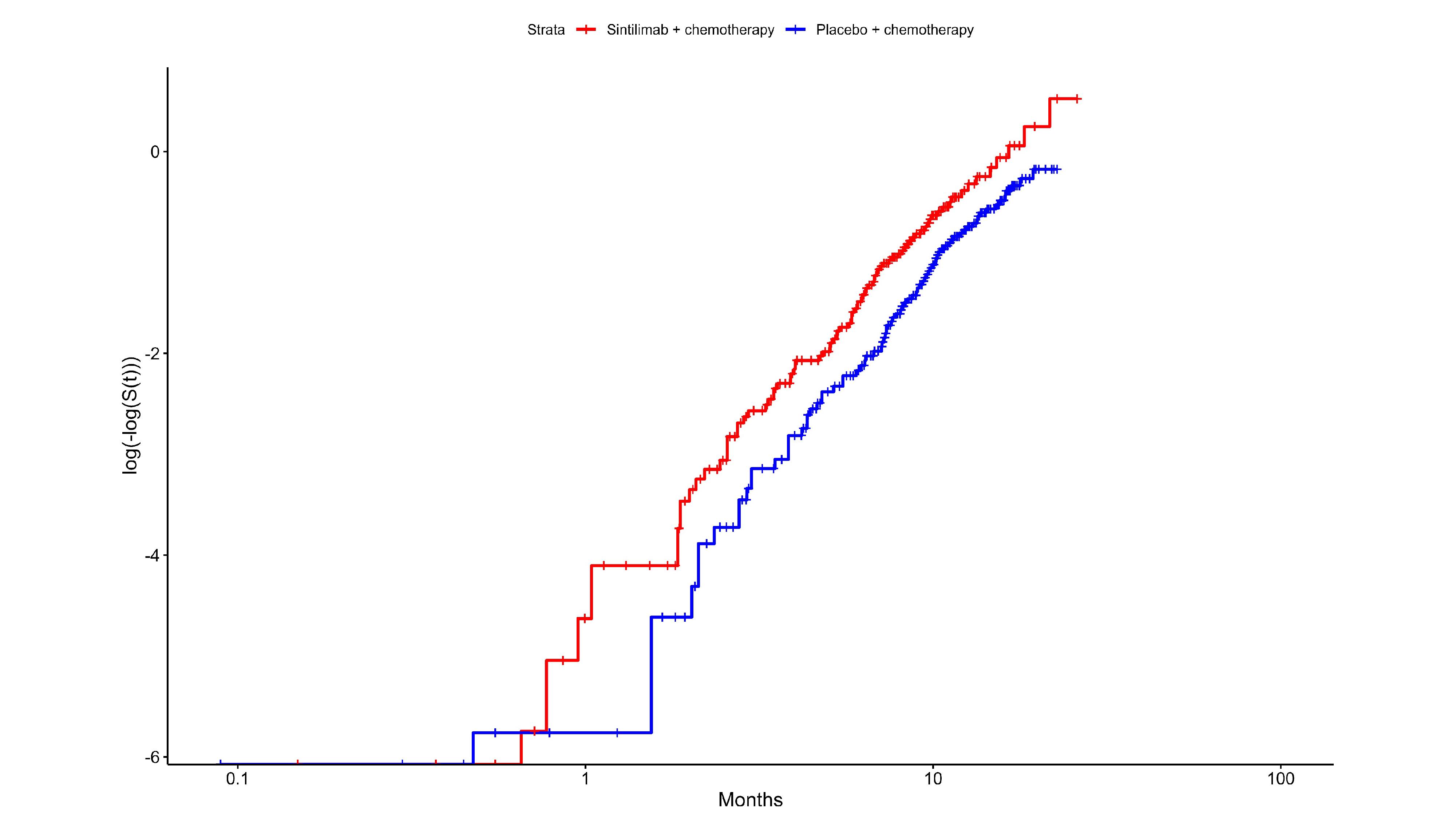

Supplement: Supplemental Material [file IANN_A_2482019_SM1981.zip › suppl_data/Figure S19. Log-cumulative plot of OS in ORIENT-15.tiff]

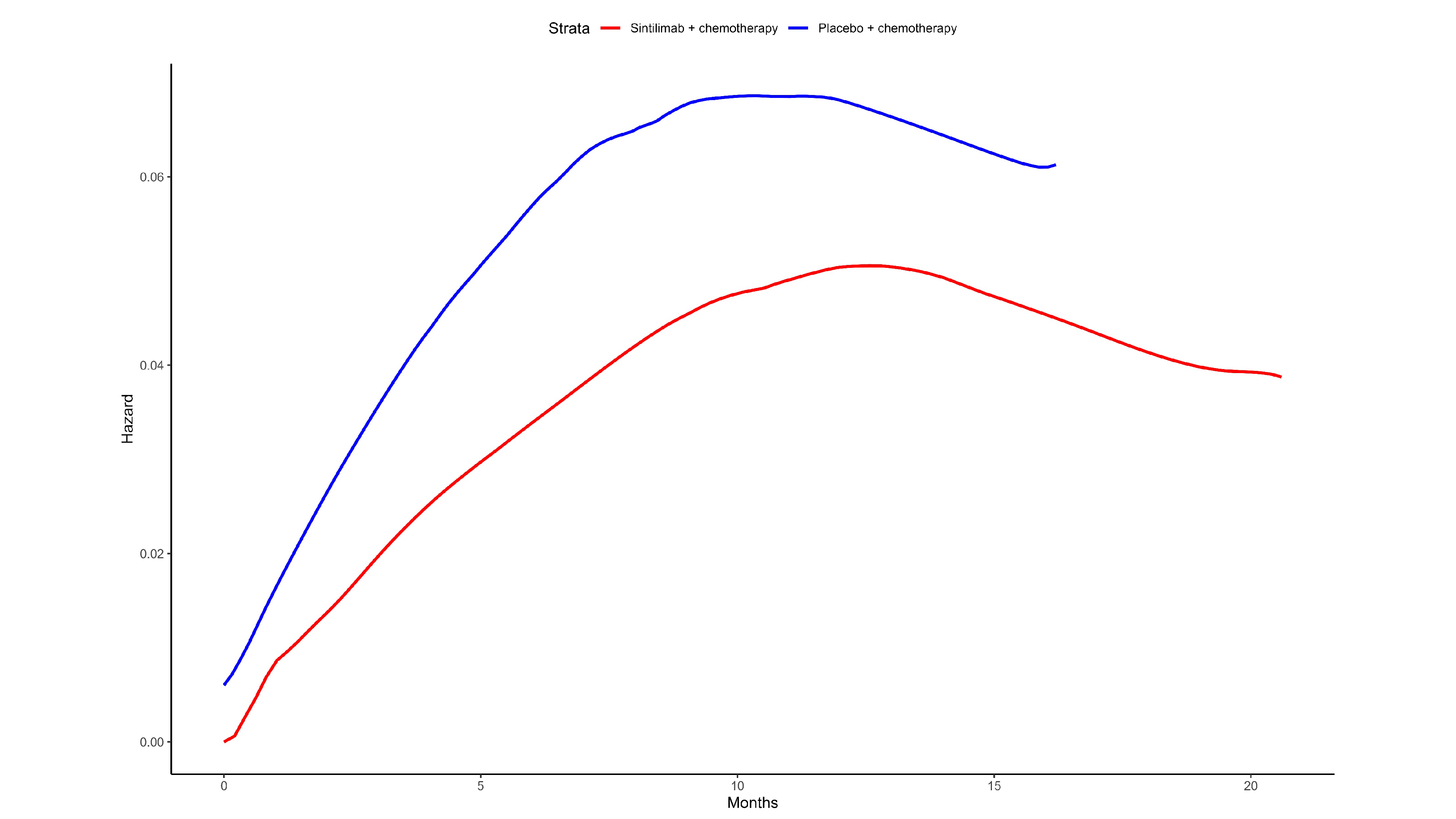

Supplement: Supplemental Material [file IANN_A_2482019_SM1981.zip › suppl_data/Figure S20. Smoothed hazard functions of OS in ORIENT-15.tiff]

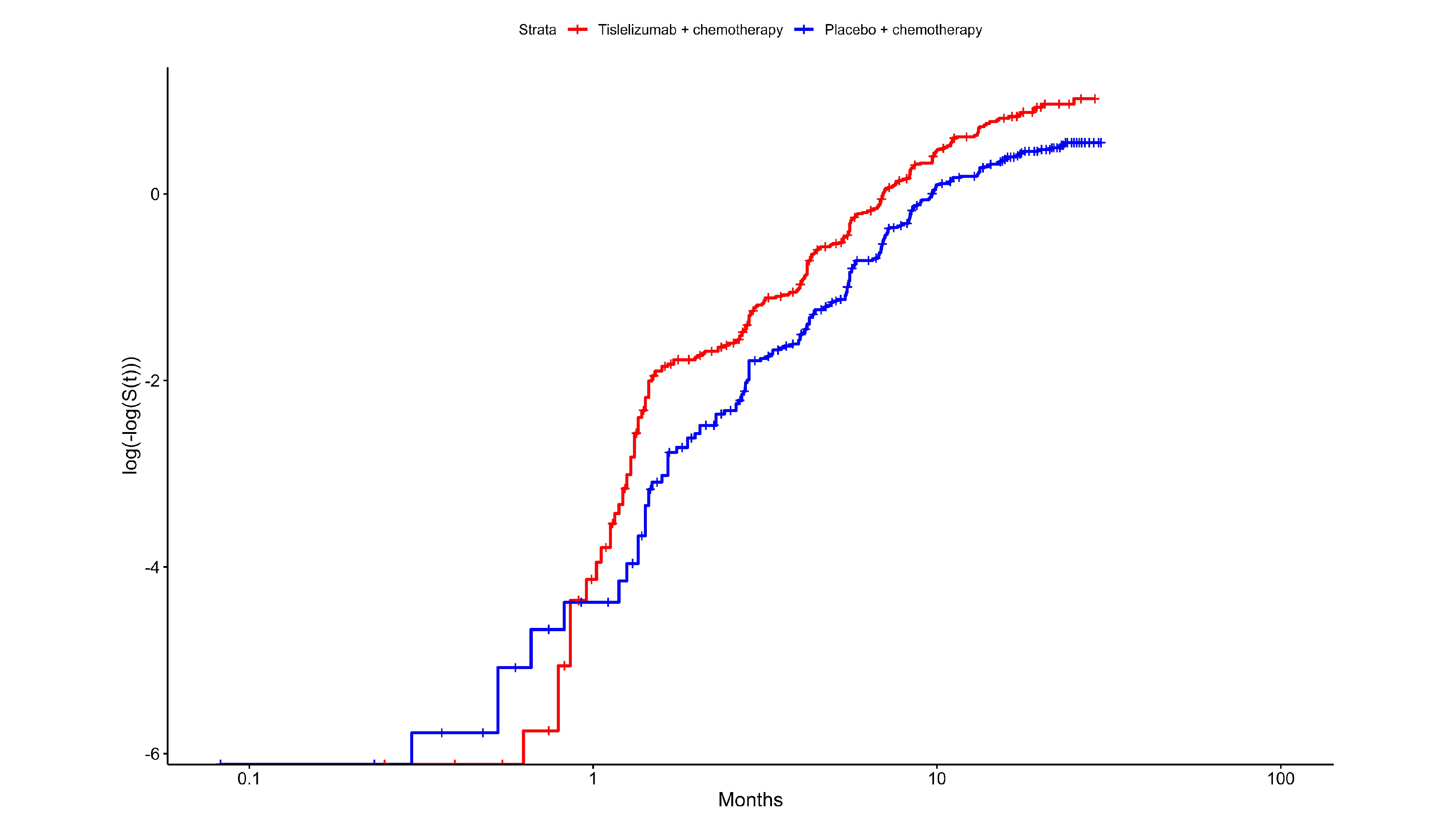

Supplement: Supplemental Material [file IANN_A_2482019_SM1981.zip › suppl_data/Figure S21. Log-cumulative plot of PFS in RATIONALE-306.tiff]

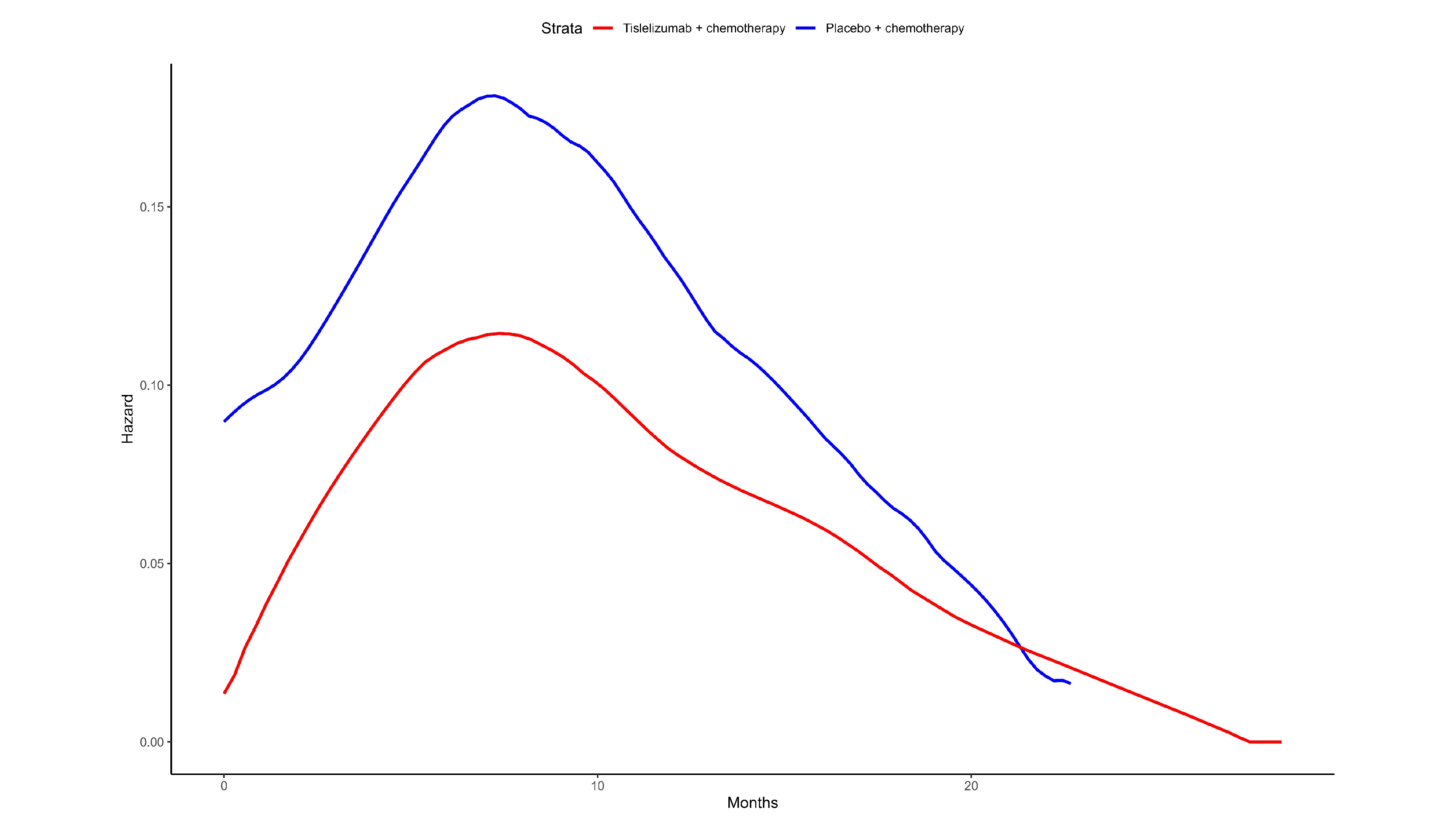

Supplement: Supplemental Material [file IANN_A_2482019_SM1981.zip › suppl_data/Figure S22. Smoothed hazard functions of PFS in RATIONALE-306.tiff]

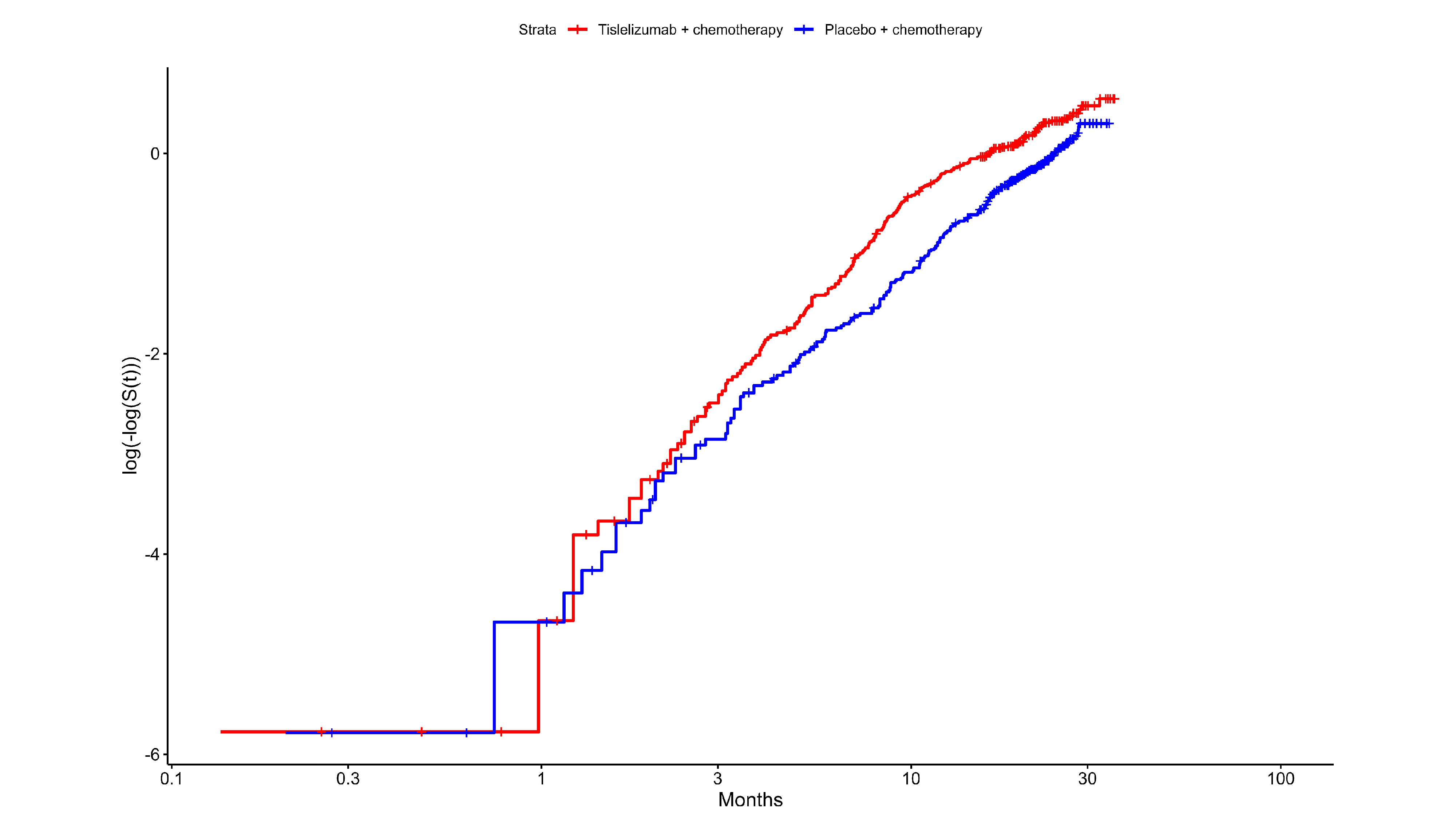

Supplement: Supplemental Material [file IANN_A_2482019_SM1981.zip › suppl_data/Figure S23. Log-cumulative plot of OS in RATIONALE-306.tiff]

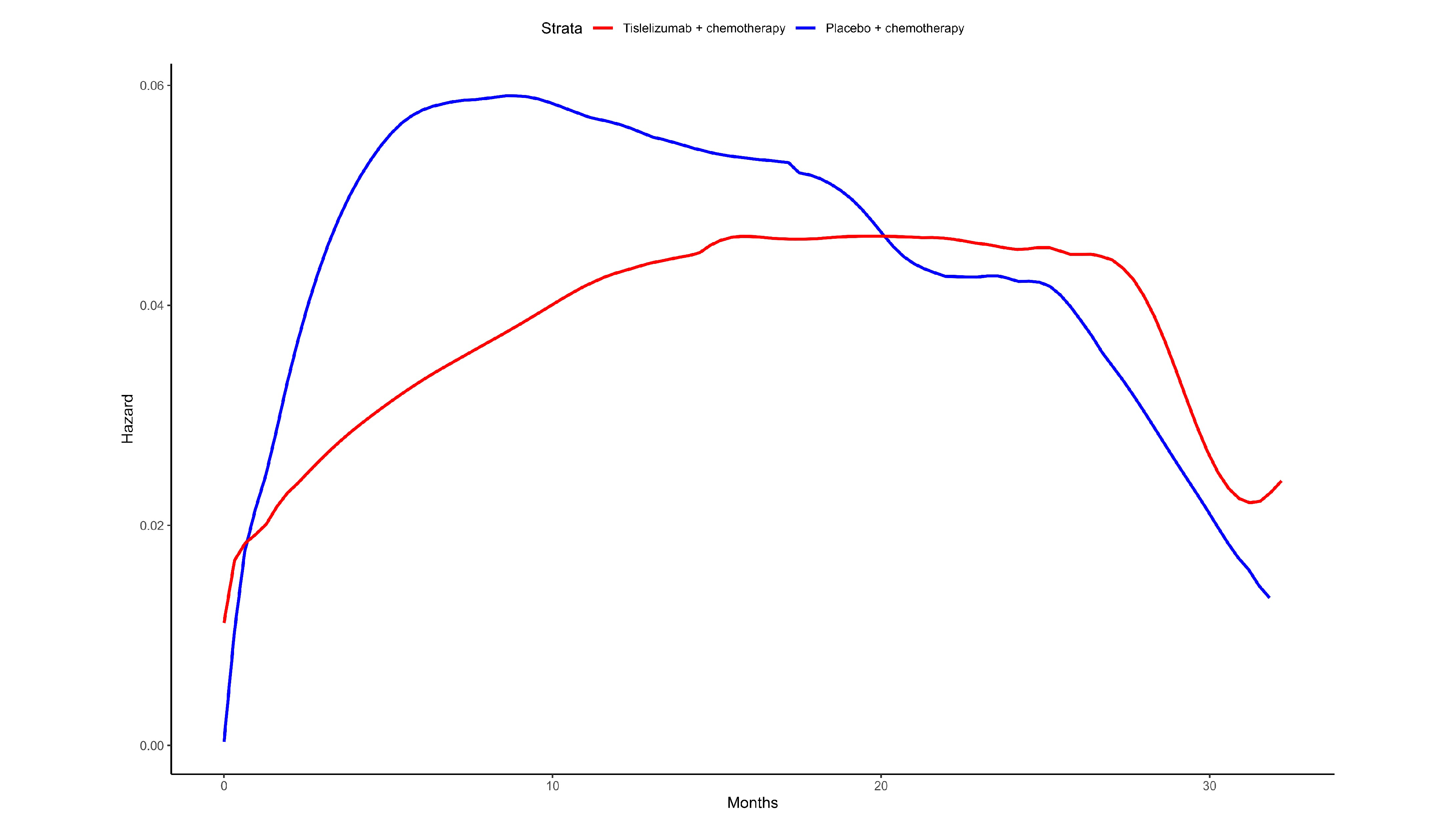

Supplement: Supplemental Material [file IANN_A_2482019_SM1981.zip › suppl_data/Figure S24. Smoothed hazard functions of OS in RATIONALE-306.tiff]

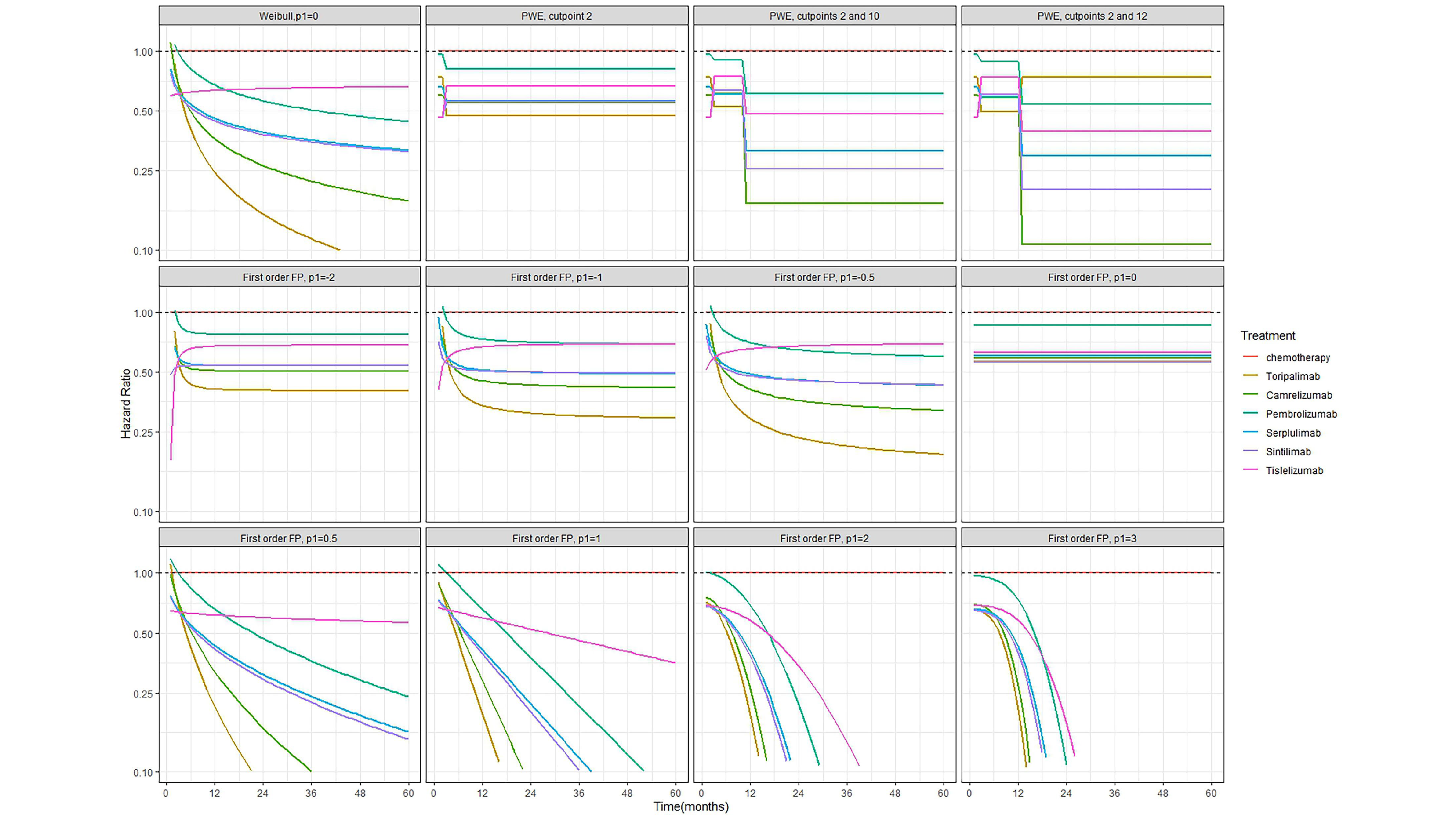

Supplement: Supplemental Material [file IANN_A_2482019_SM1981.zip › suppl_data/Figure S25. Hazard ratio of PFS (1-12).tiff]

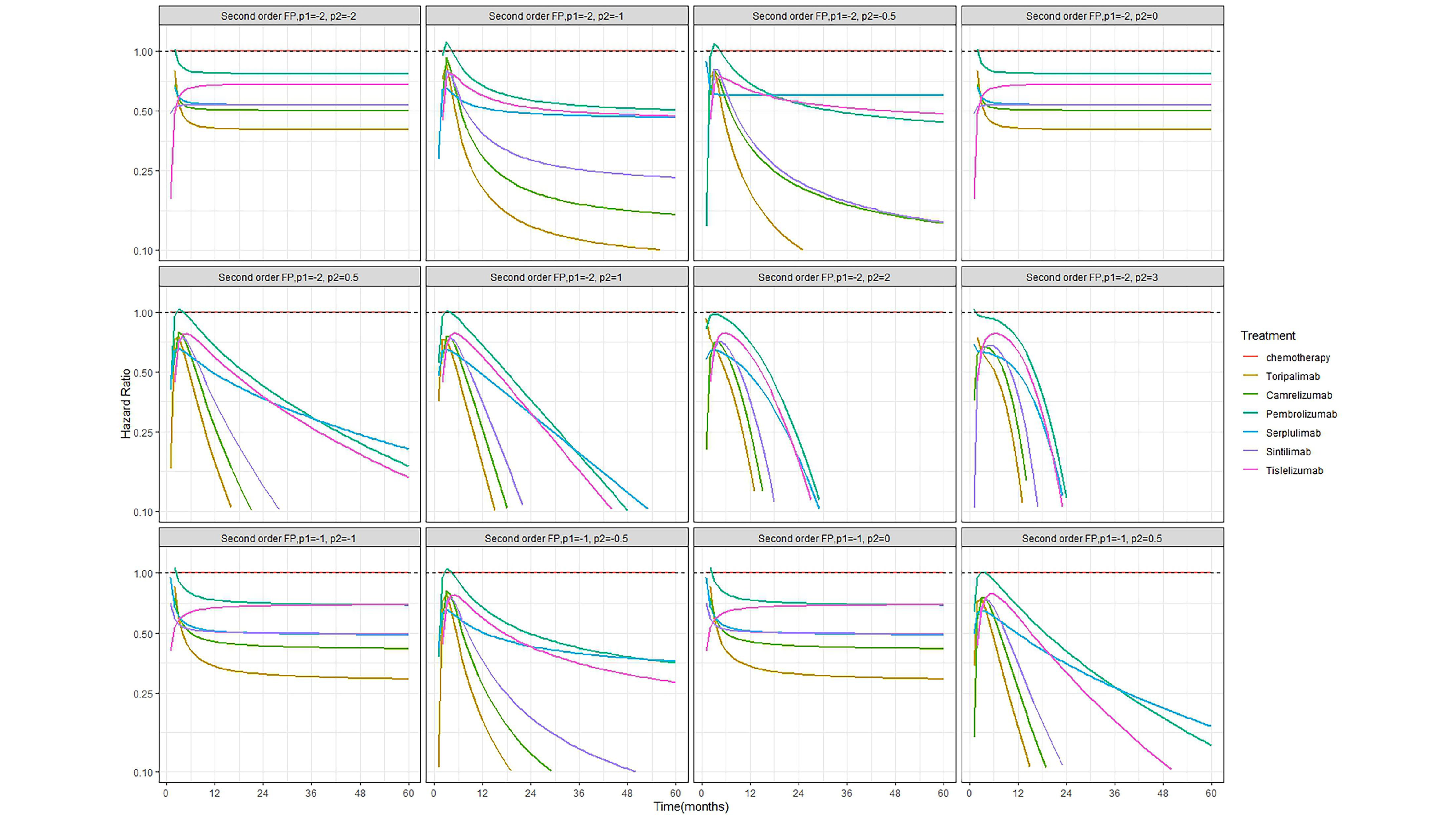

Supplement: Supplemental Material [file IANN_A_2482019_SM1981.zip › suppl_data/Figure S26. Hazard ratio of PFS (13-24).tiff]

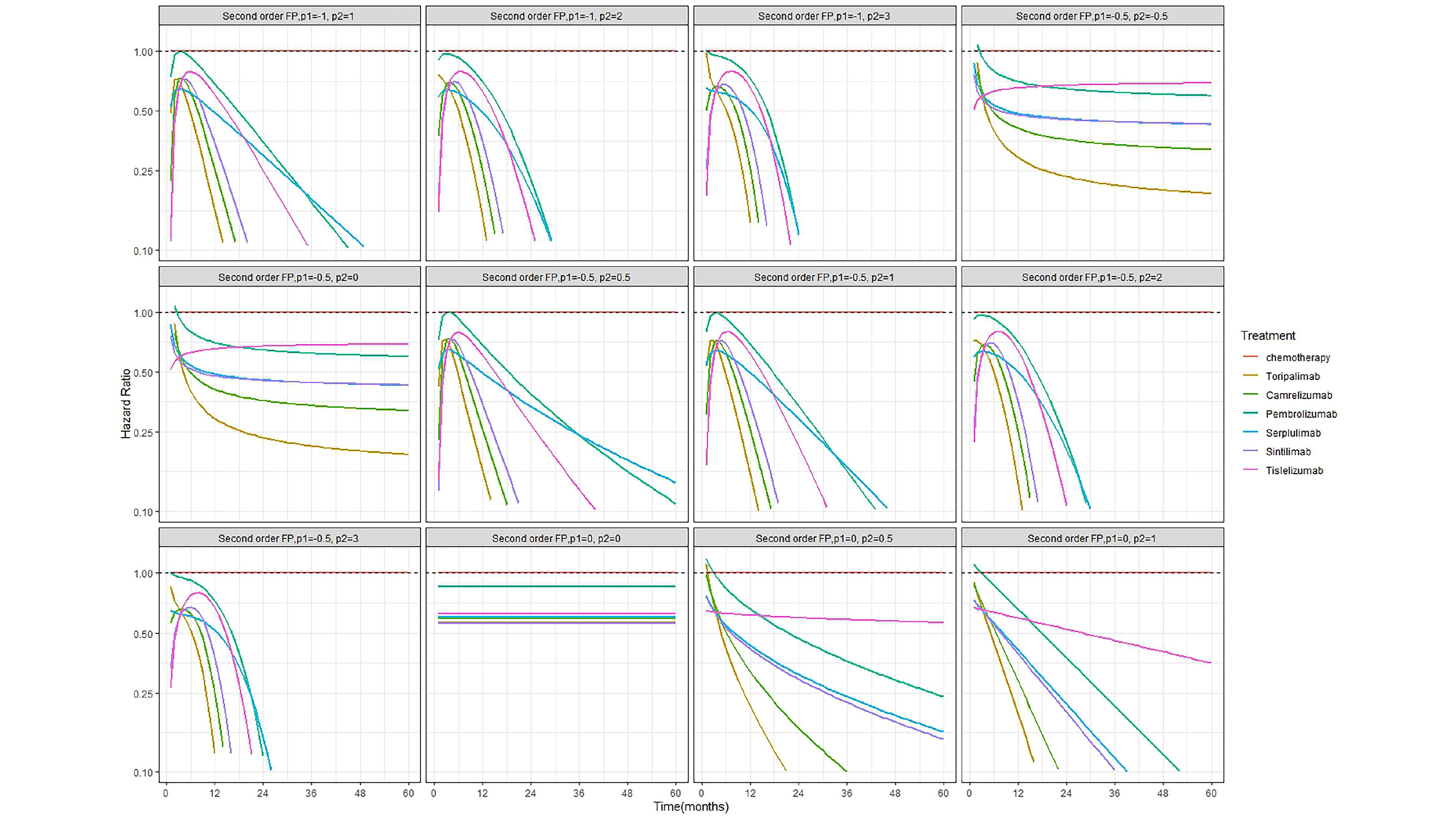

Supplement: Supplemental Material [file IANN_A_2482019_SM1981.zip › suppl_data/Figure S27. Hazard ratio of PFS (25-36).tiff]

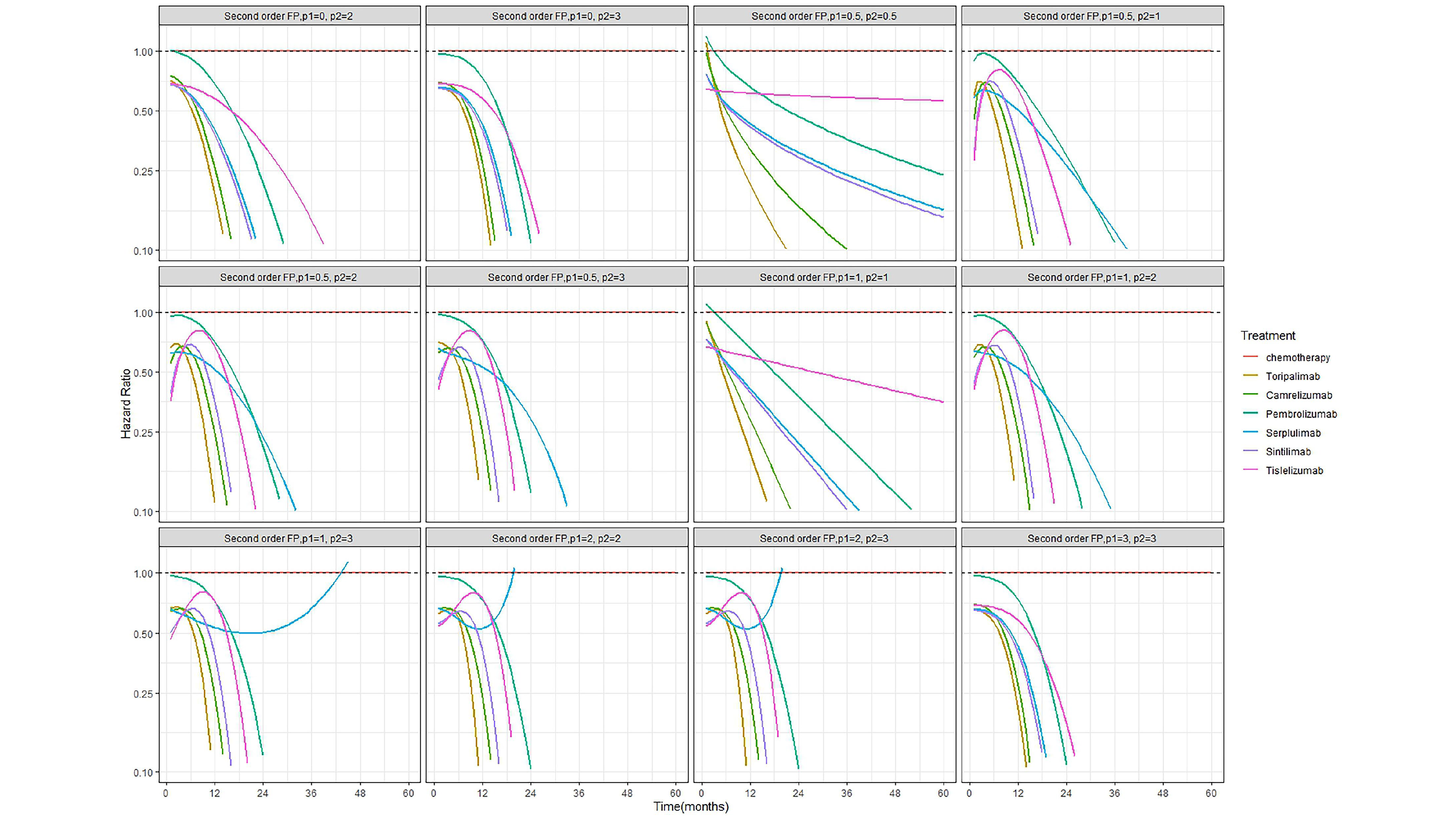

Supplement: Supplemental Material [file IANN_A_2482019_SM1981.zip › suppl_data/Figure S28. Hazard ratio of PFS (37-48).tiff]

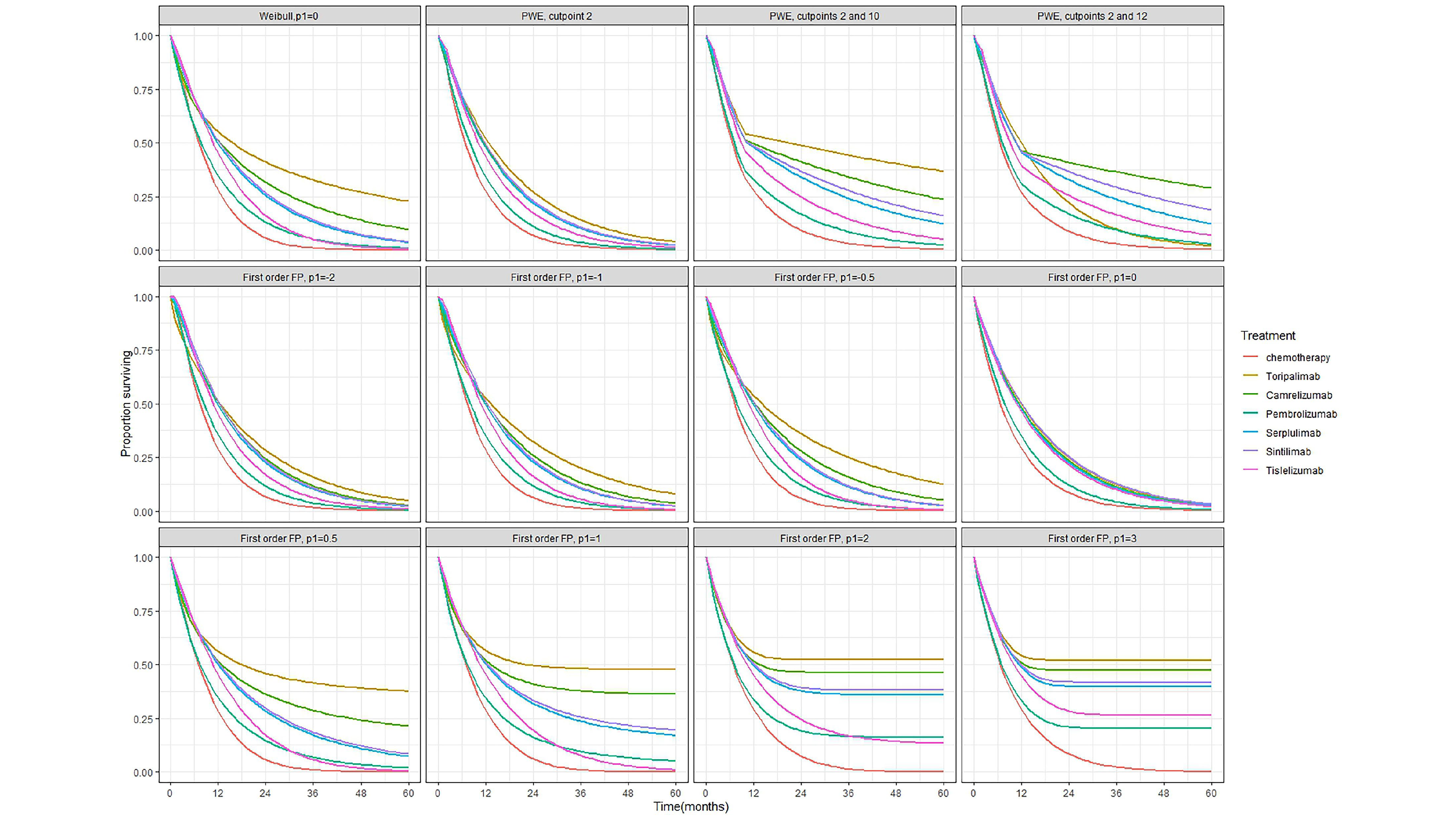

Supplement: Supplemental Material [file IANN_A_2482019_SM1981.zip › suppl_data/Figure S29. Survival curve of PFS (1-12).tiff]

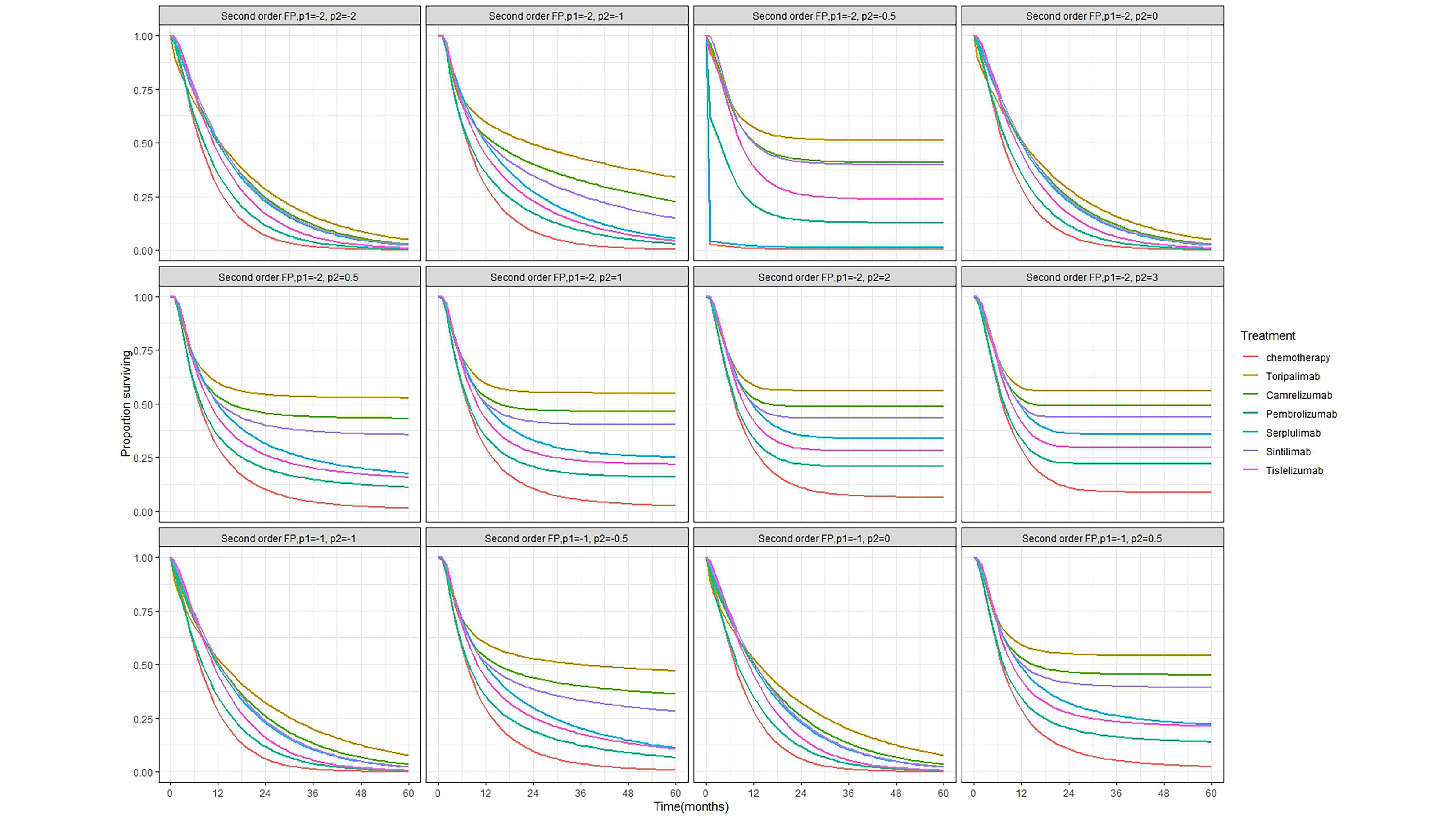

Supplement: Supplemental Material [file IANN_A_2482019_SM1981.zip › suppl_data/Figure S30. Survival curve of PFS (13-24).tiff]

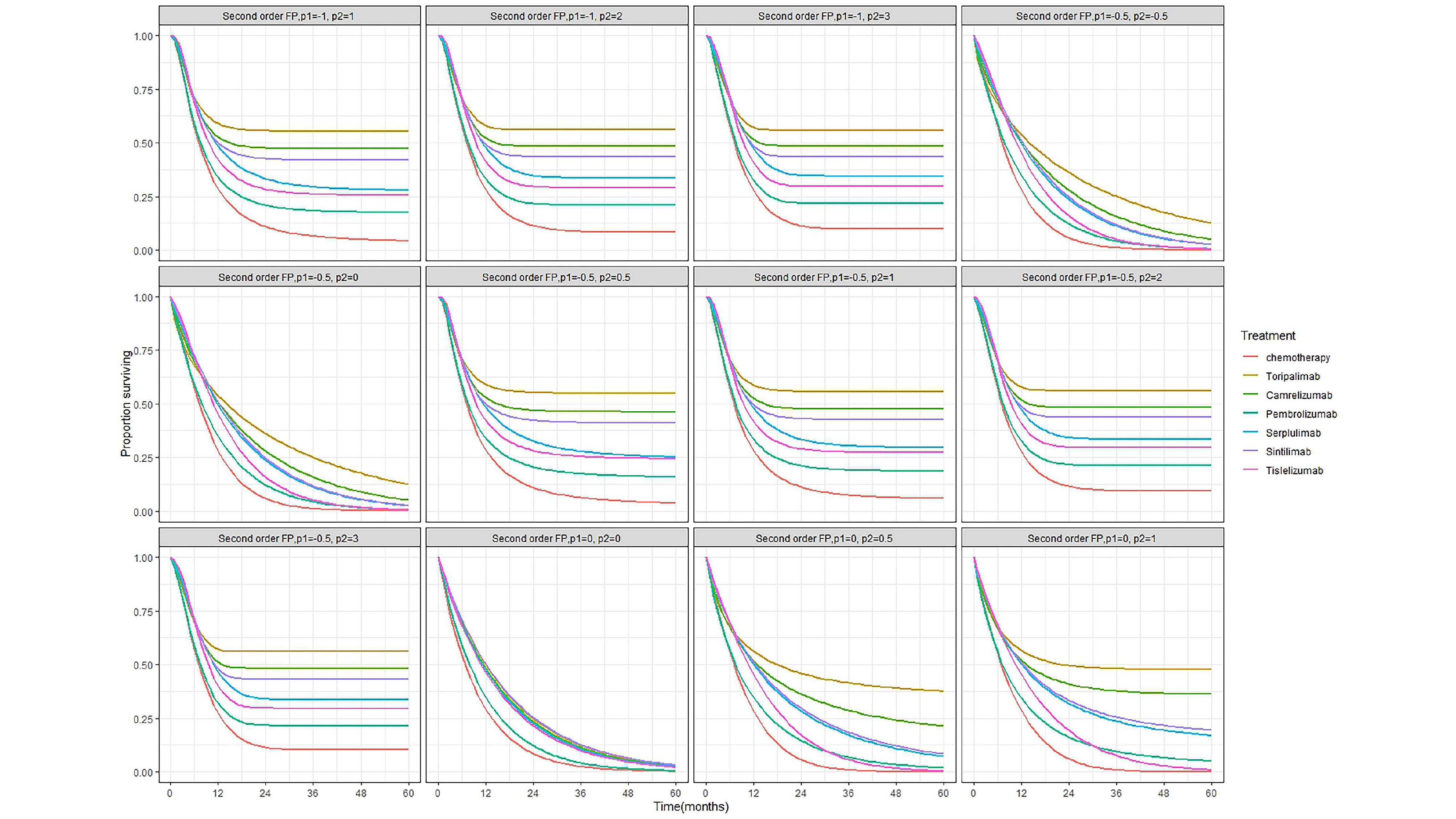

Supplement: Supplemental Material [file IANN_A_2482019_SM1981.zip › suppl_data/Figure S31. Survival curve of PFS (25-36).tiff]

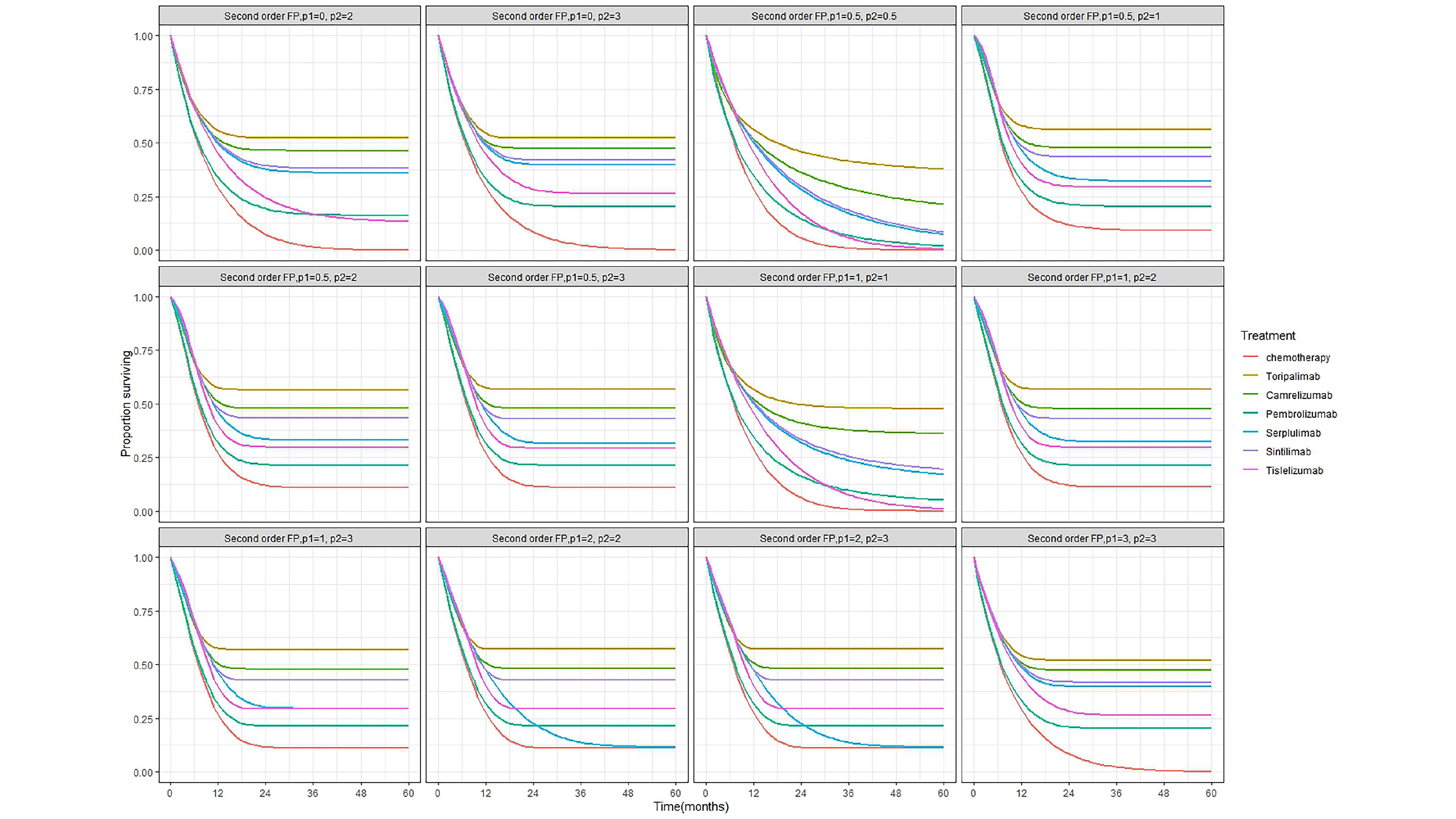

Supplement: Supplemental Material [file IANN_A_2482019_SM1981.zip › suppl_data/Figure S32. Survival curve of PFS (37-48).tiff]

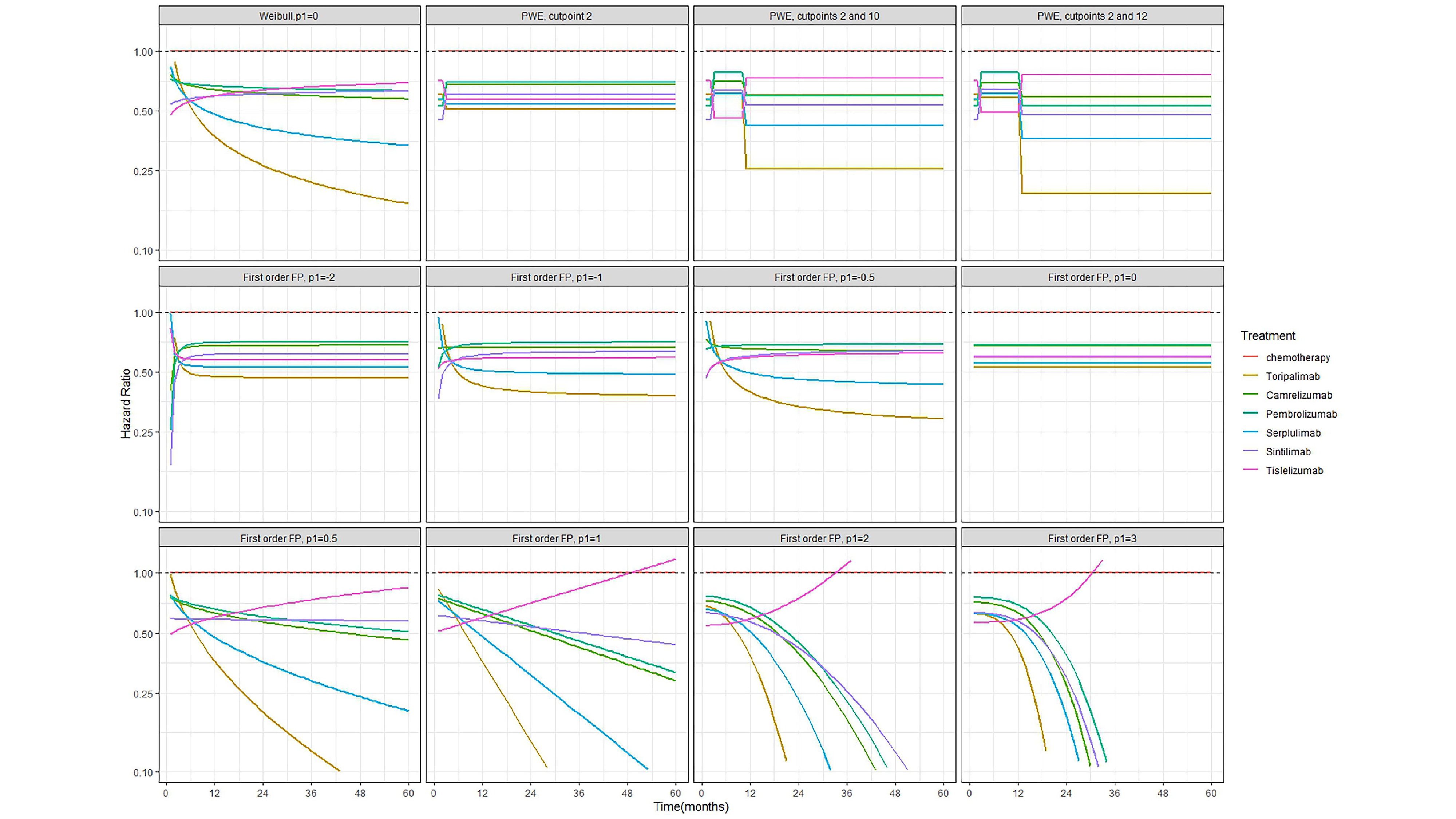

Supplement: Supplemental Material [file IANN_A_2482019_SM1981.zip › suppl_data/Figure S33. Hazard ratio of OS (1-12).tiff]

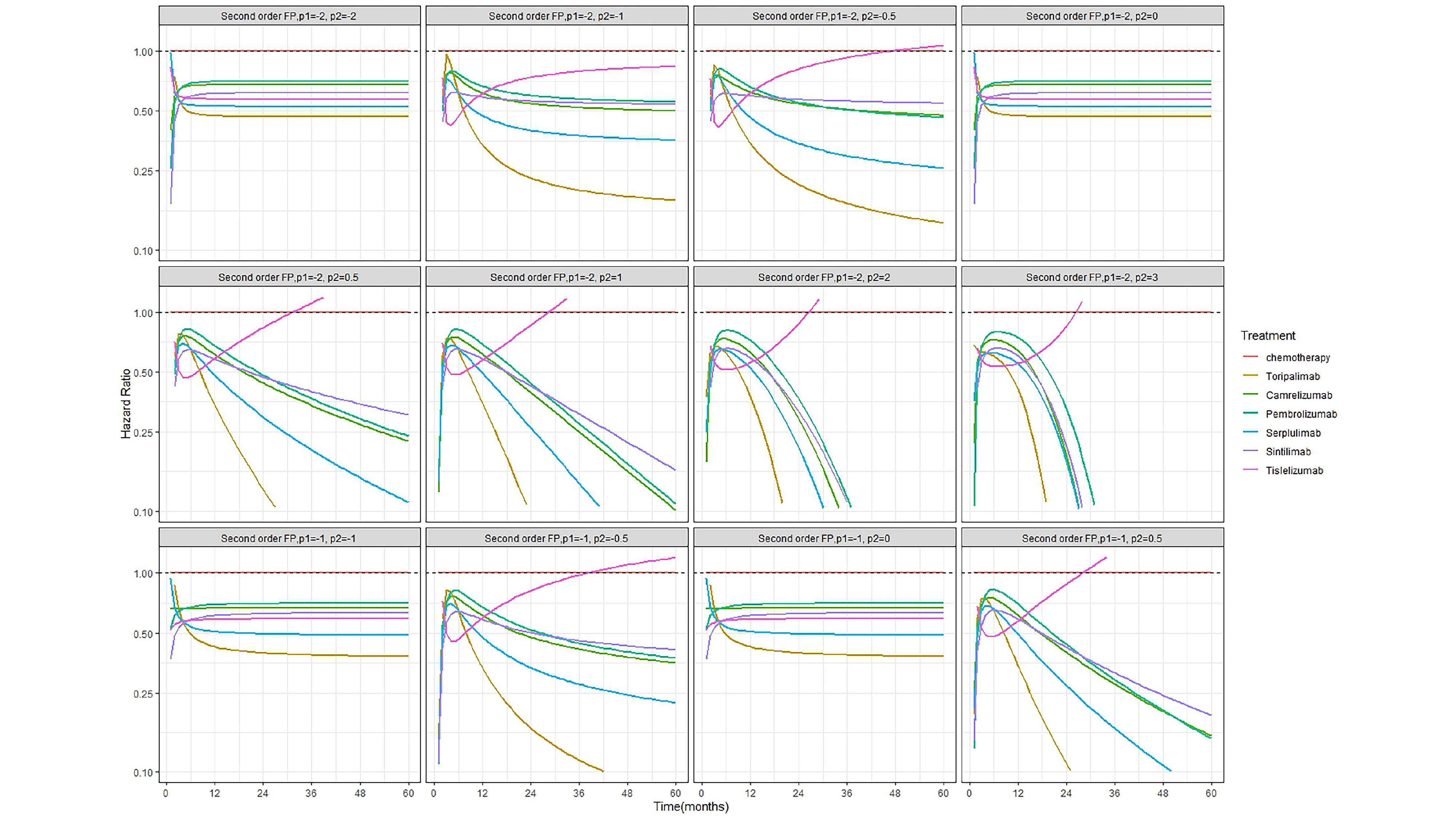

Supplement: Supplemental Material [file IANN_A_2482019_SM1981.zip › suppl_data/Figure S34. Hazard ratio of OS (13-24).tiff]

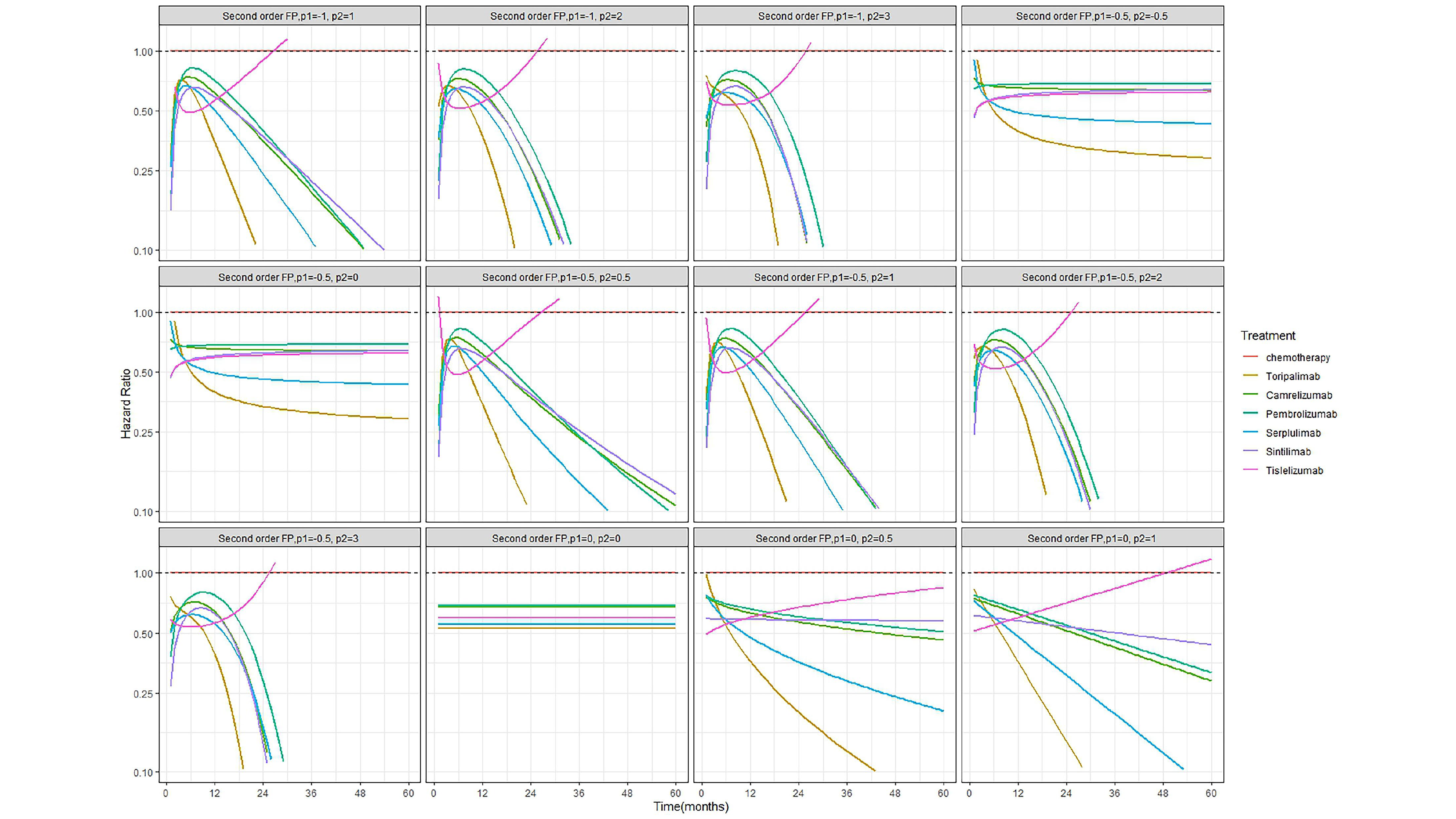

Supplement: Supplemental Material [file IANN_A_2482019_SM1981.zip › suppl_data/Figure S35. Hazard ratio of OS (25-36).tiff]

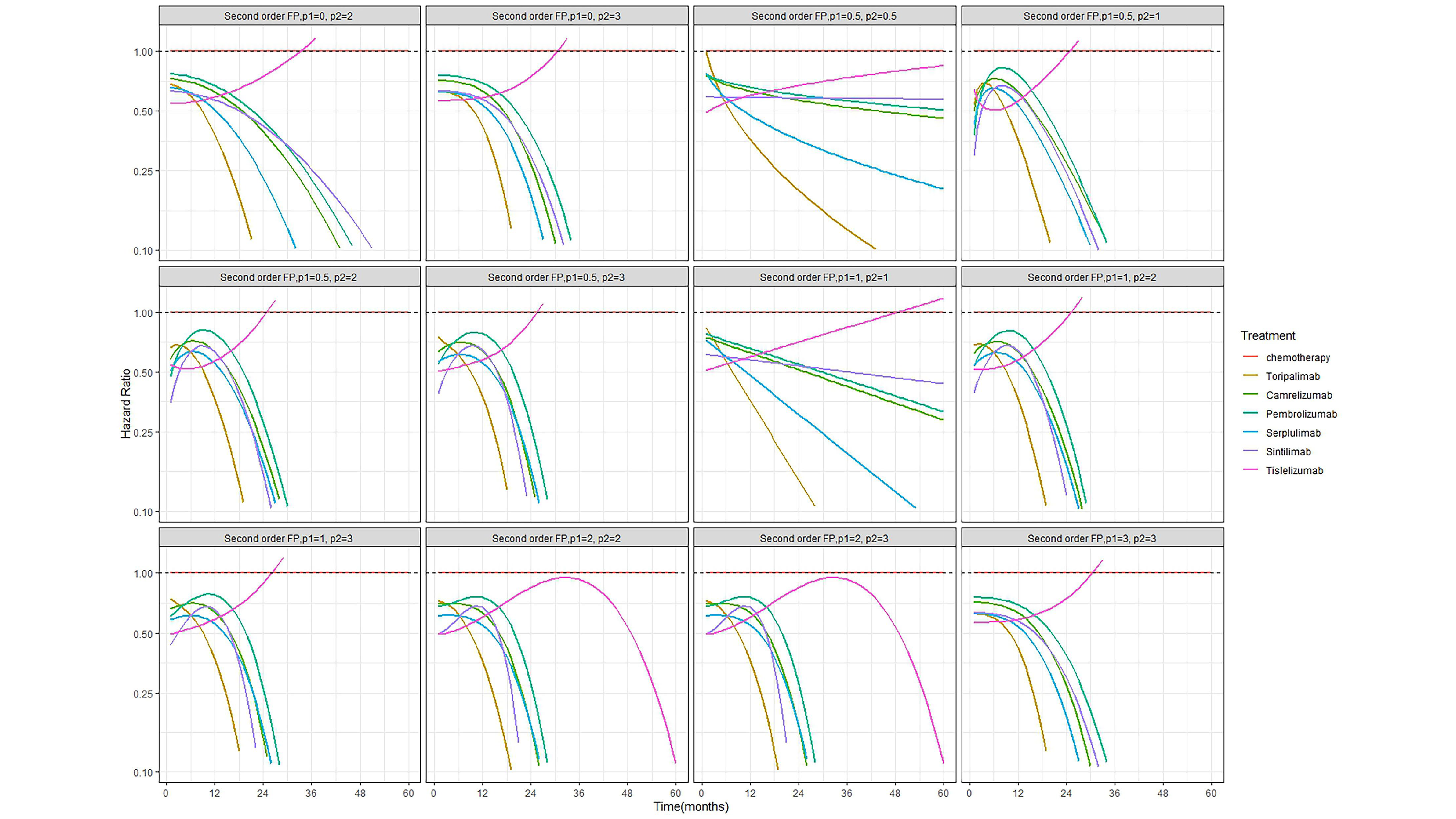

Supplement: Supplemental Material [file IANN_A_2482019_SM1981.zip › suppl_data/Figure S36. Hazard ratio of OS (37-48).tiff]

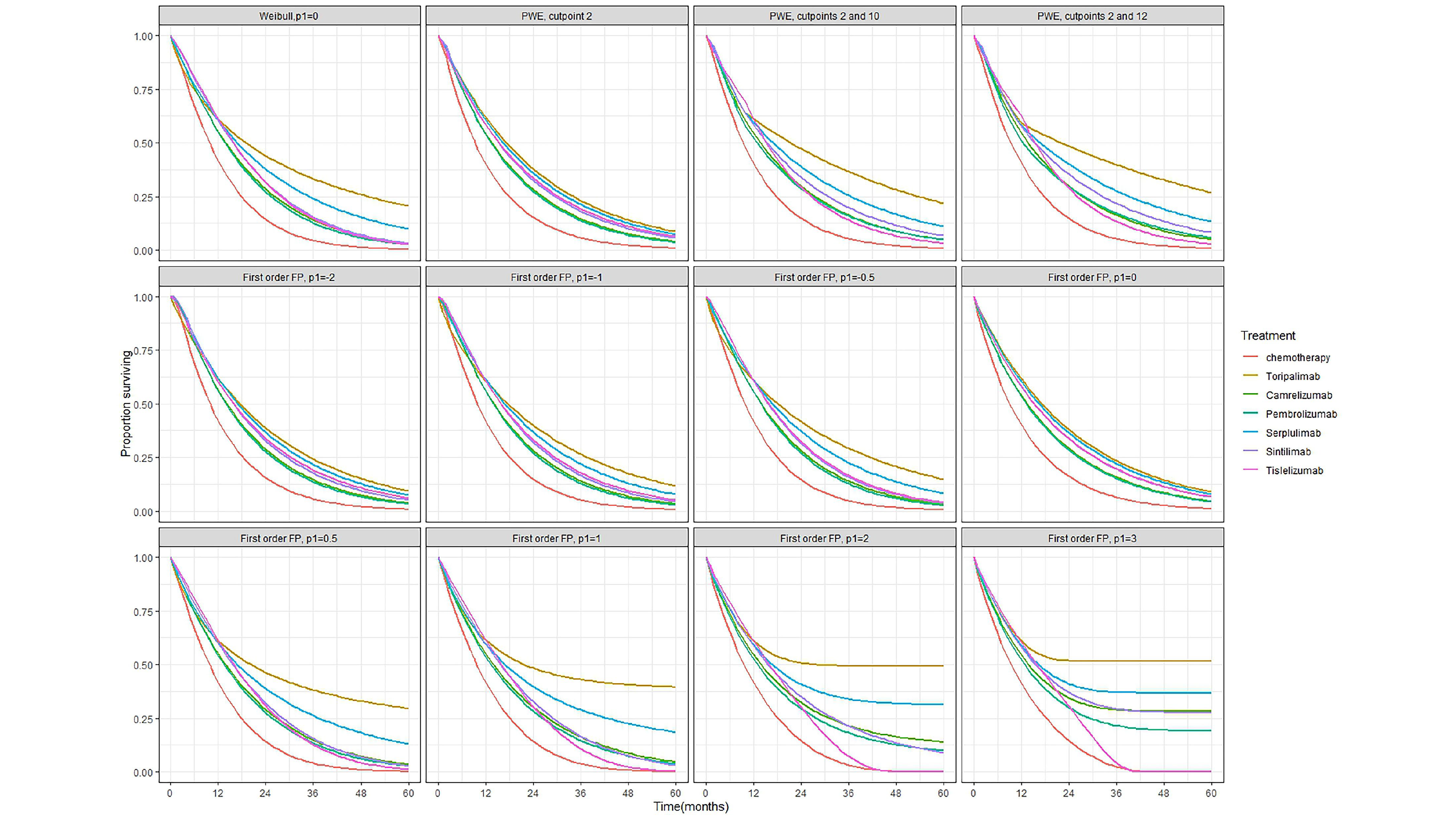

Supplement: Supplemental Material [file IANN_A_2482019_SM1981.zip › suppl_data/Figure S37. Survival curve of OS (1-12).tiff]

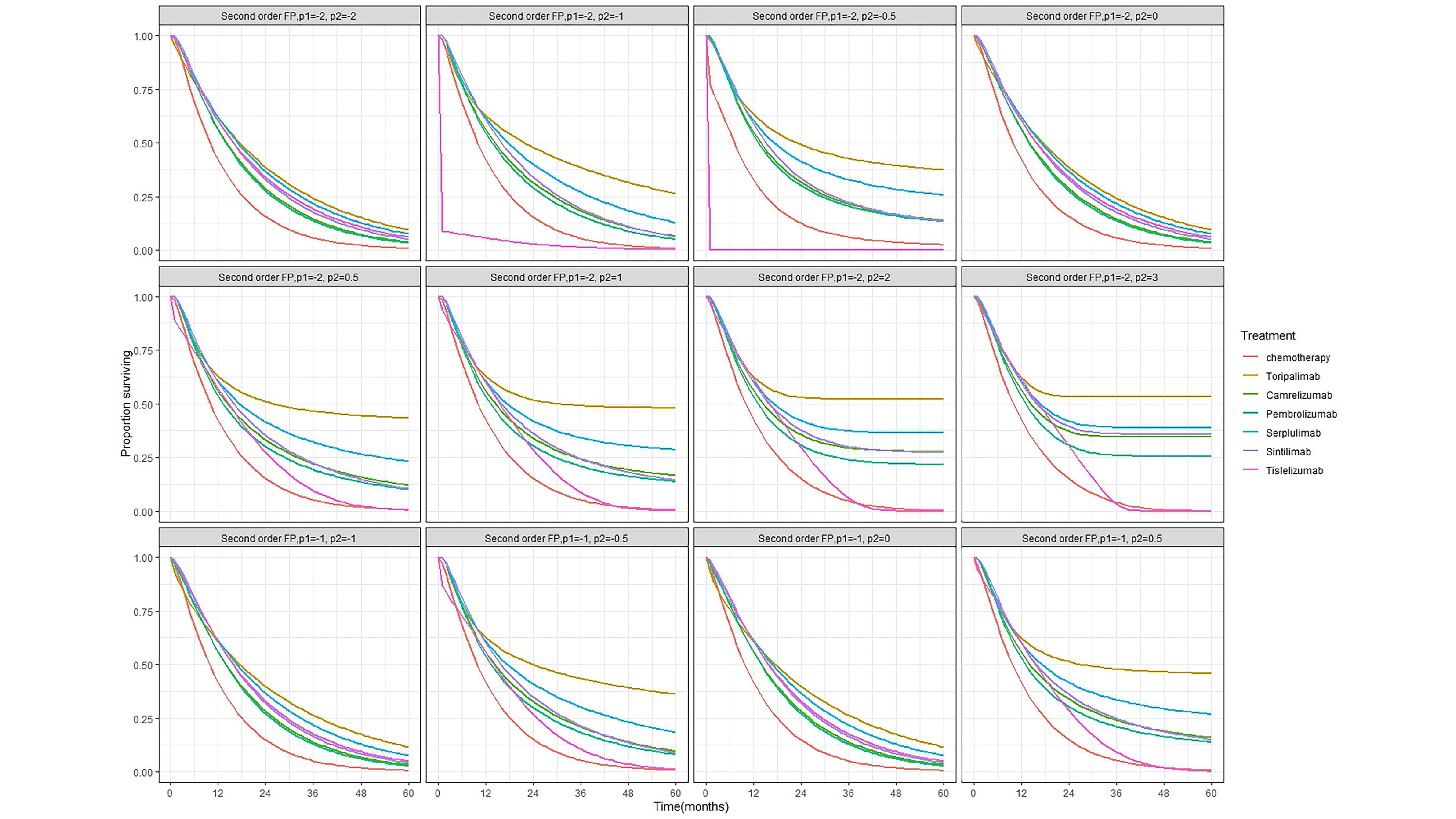

Supplement: Supplemental Material [file IANN_A_2482019_SM1981.zip › suppl_data/Figure S38. Survival curve of OS (13-24).tiff]

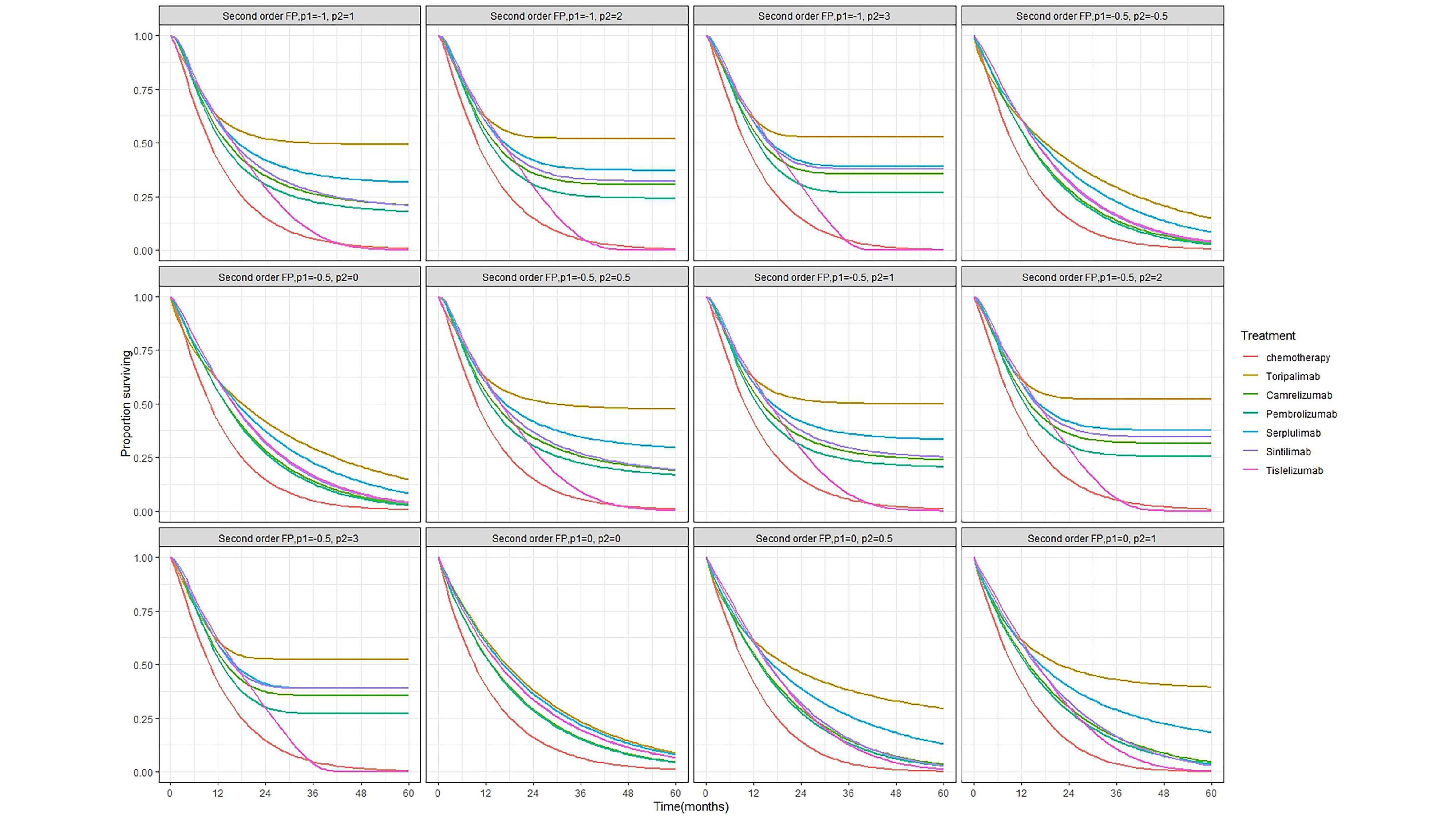

Supplement: Supplemental Material [file IANN_A_2482019_SM1981.zip › suppl_data/Figure S39. Survival curve of OS (25-36).tiff]

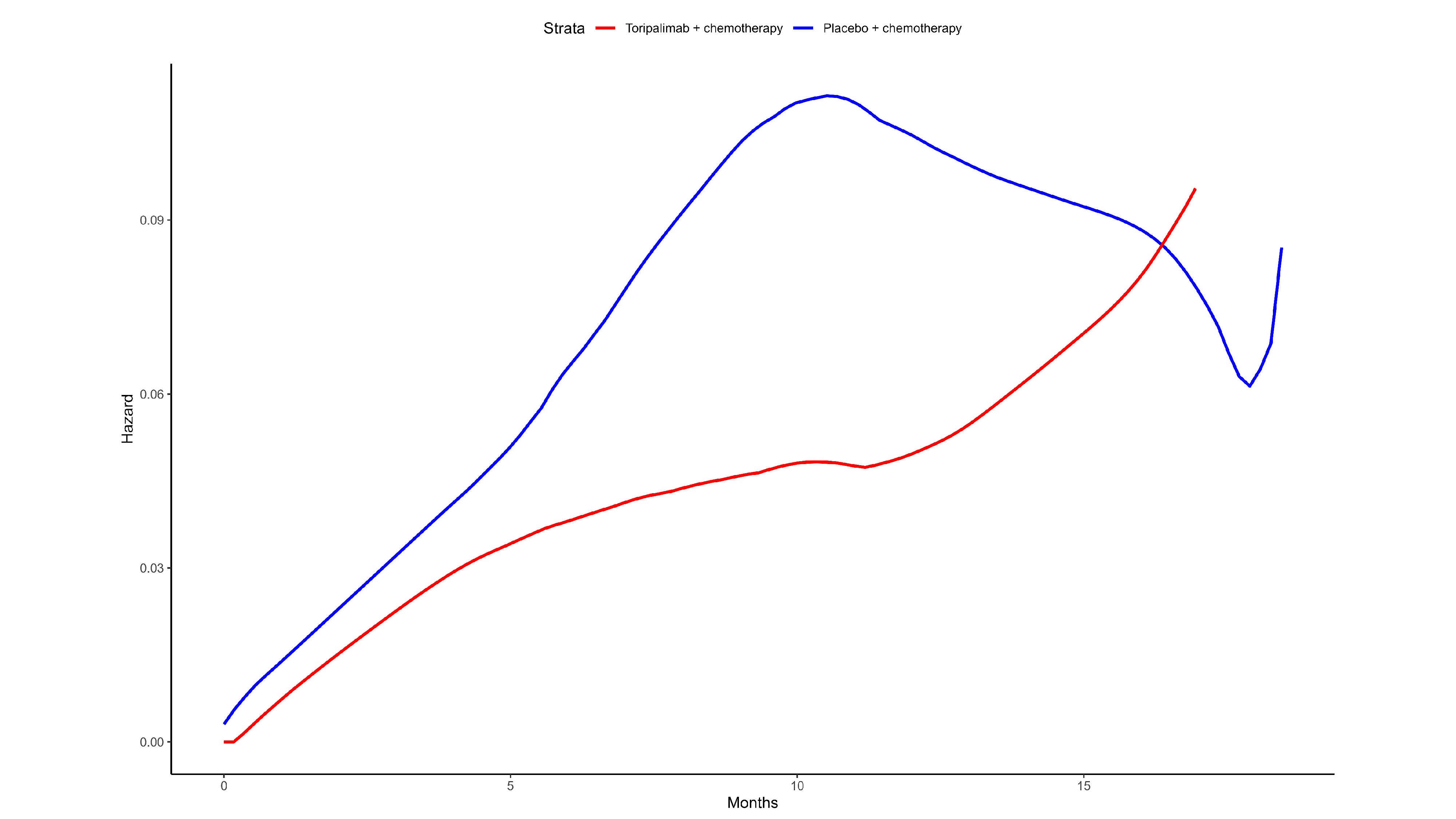

Supplement: Supplemental Material [file IANN_A_2482019_SM1981.zip › suppl_data/Figure S4. Smoothed hazard functions of OS in JUPITER-06.tiff]

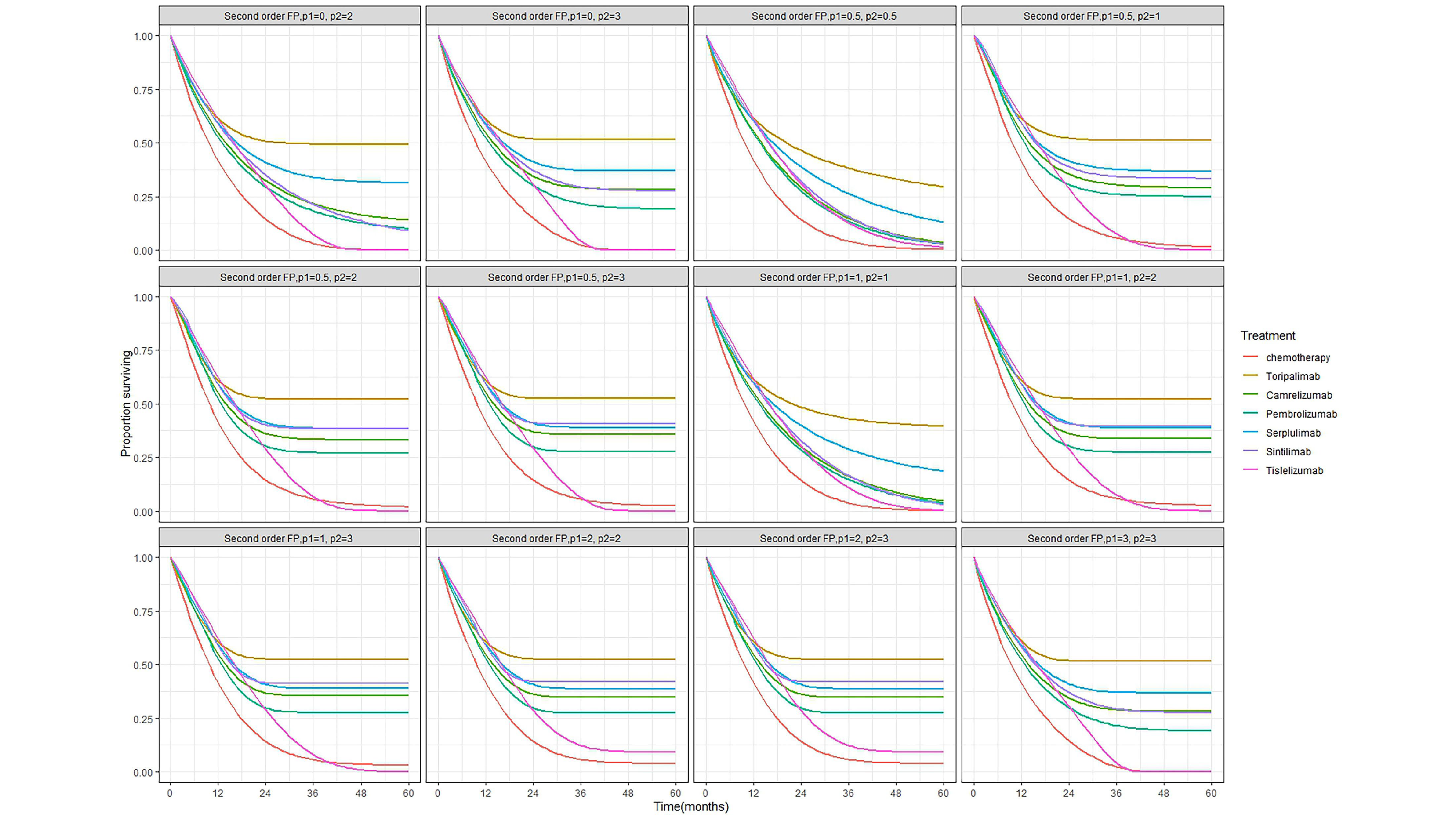

Supplement: Supplemental Material [file IANN_A_2482019_SM1981.zip › suppl_data/Figure S40. Survival curve of OS (37-48).tiff]

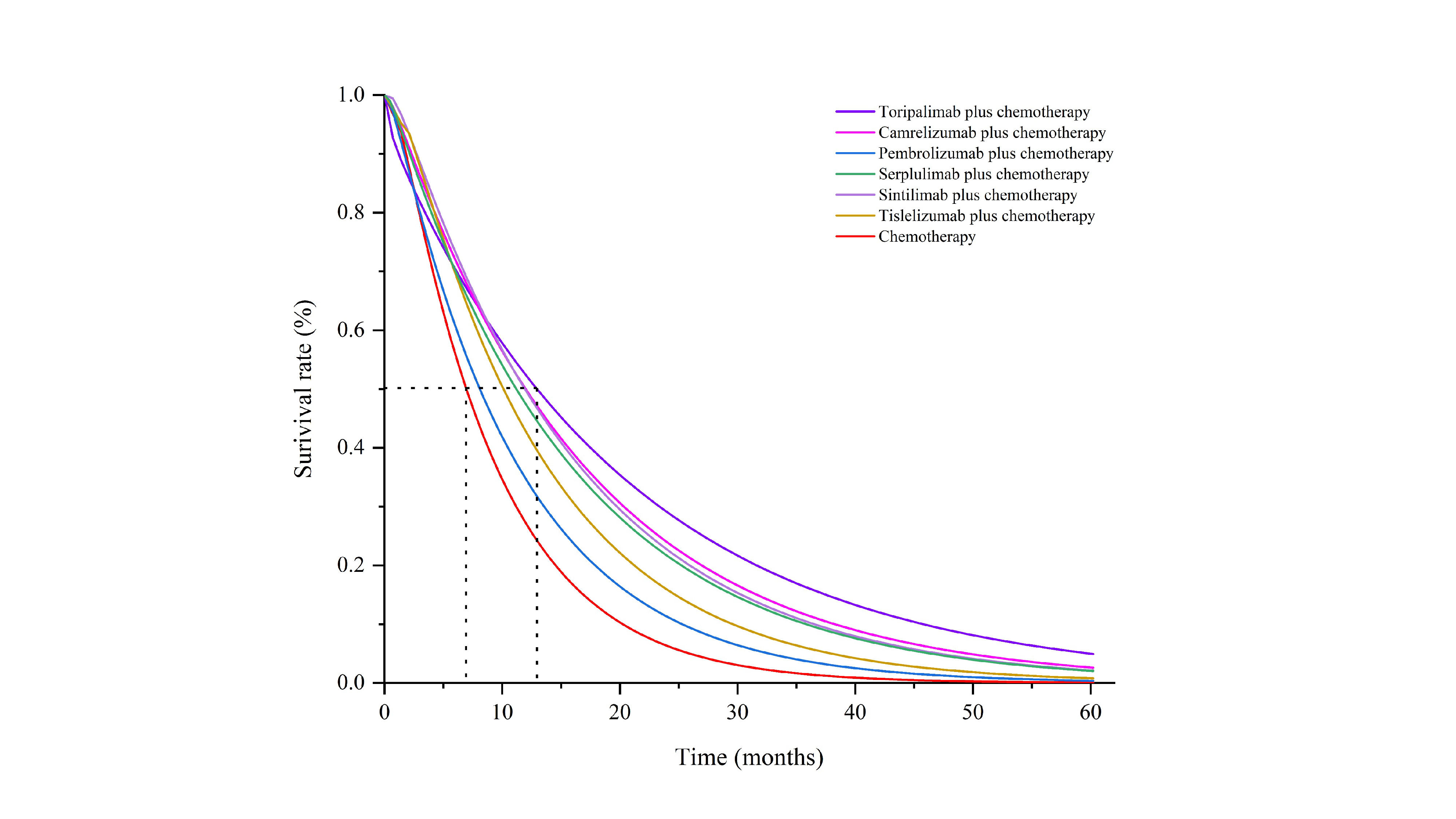

Supplement: Supplemental Material [file IANN_A_2482019_SM1981.zip › suppl_data/Figure S41. Progression-free survival curve with first order fractional polynomials (P=2).tiff]

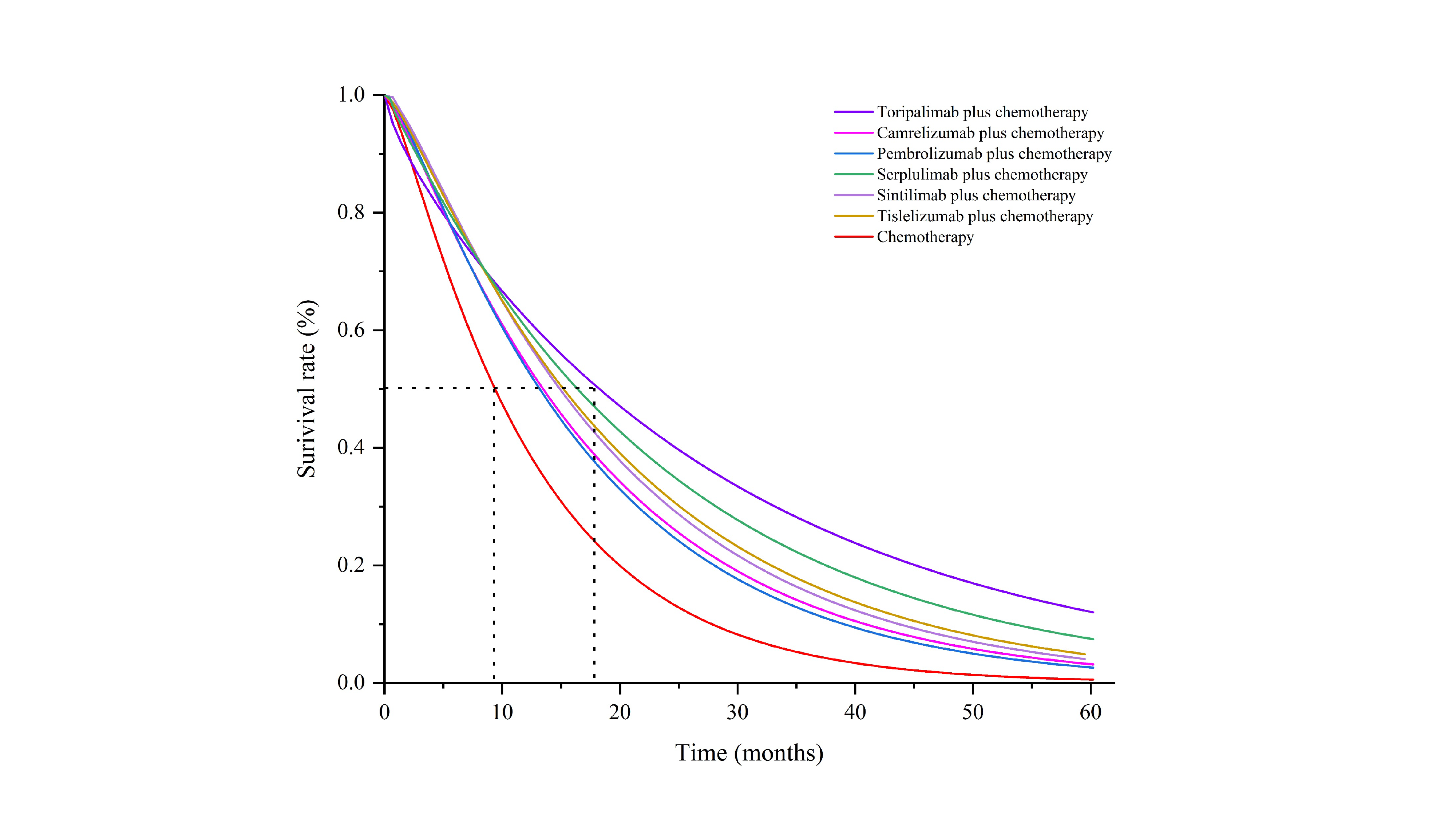

Supplement: Supplemental Material [file IANN_A_2482019_SM1981.zip › suppl_data/Figure S42. Overall survival curve with first order fractional polynomials (P=-1).tiff]

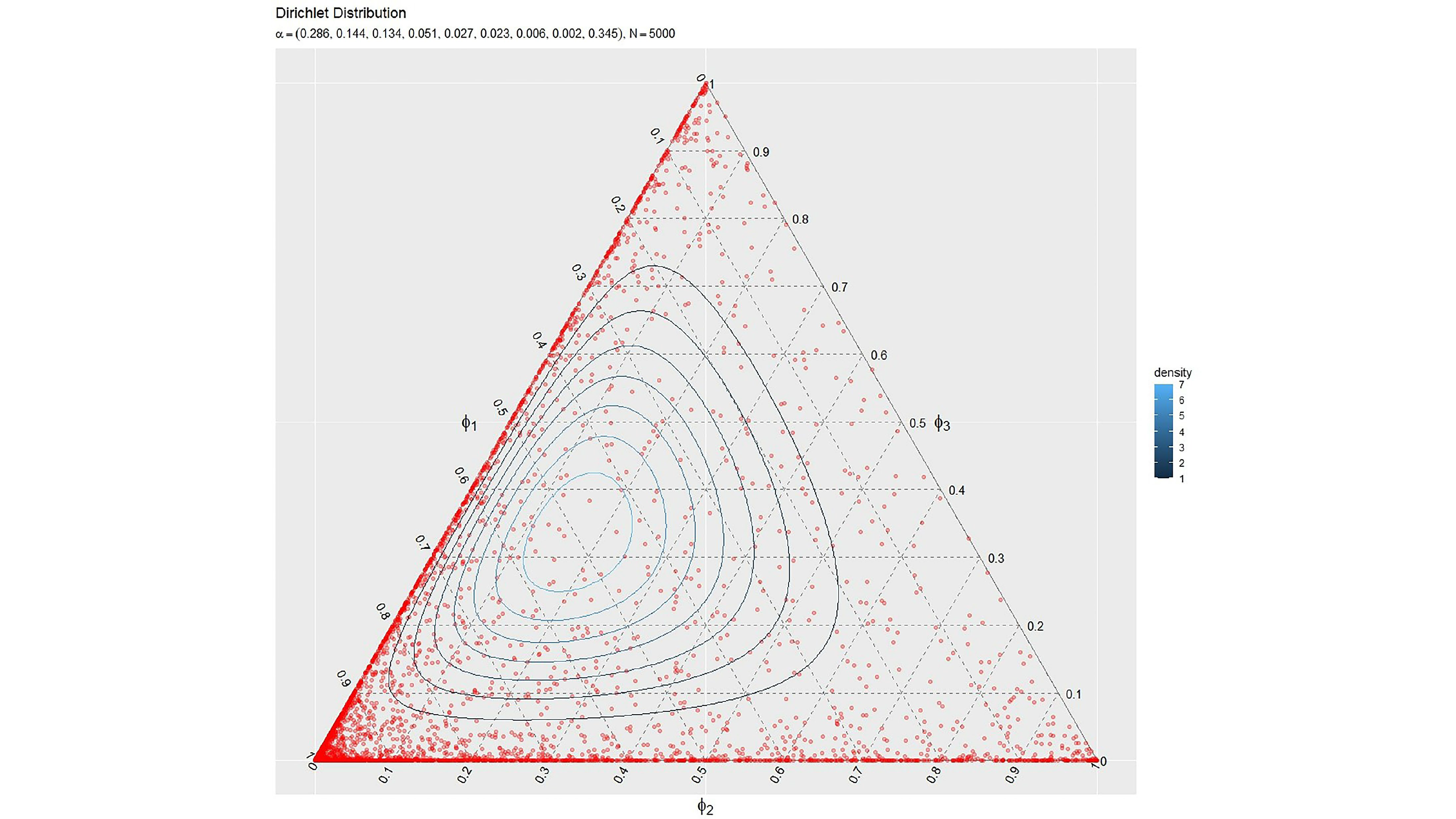

Supplement: Supplemental Material [file IANN_A_2482019_SM1981.zip › suppl_data/Figure S43. Proportion of subsequent anti-cancer therapy with Dirichlet distribu.tiff]

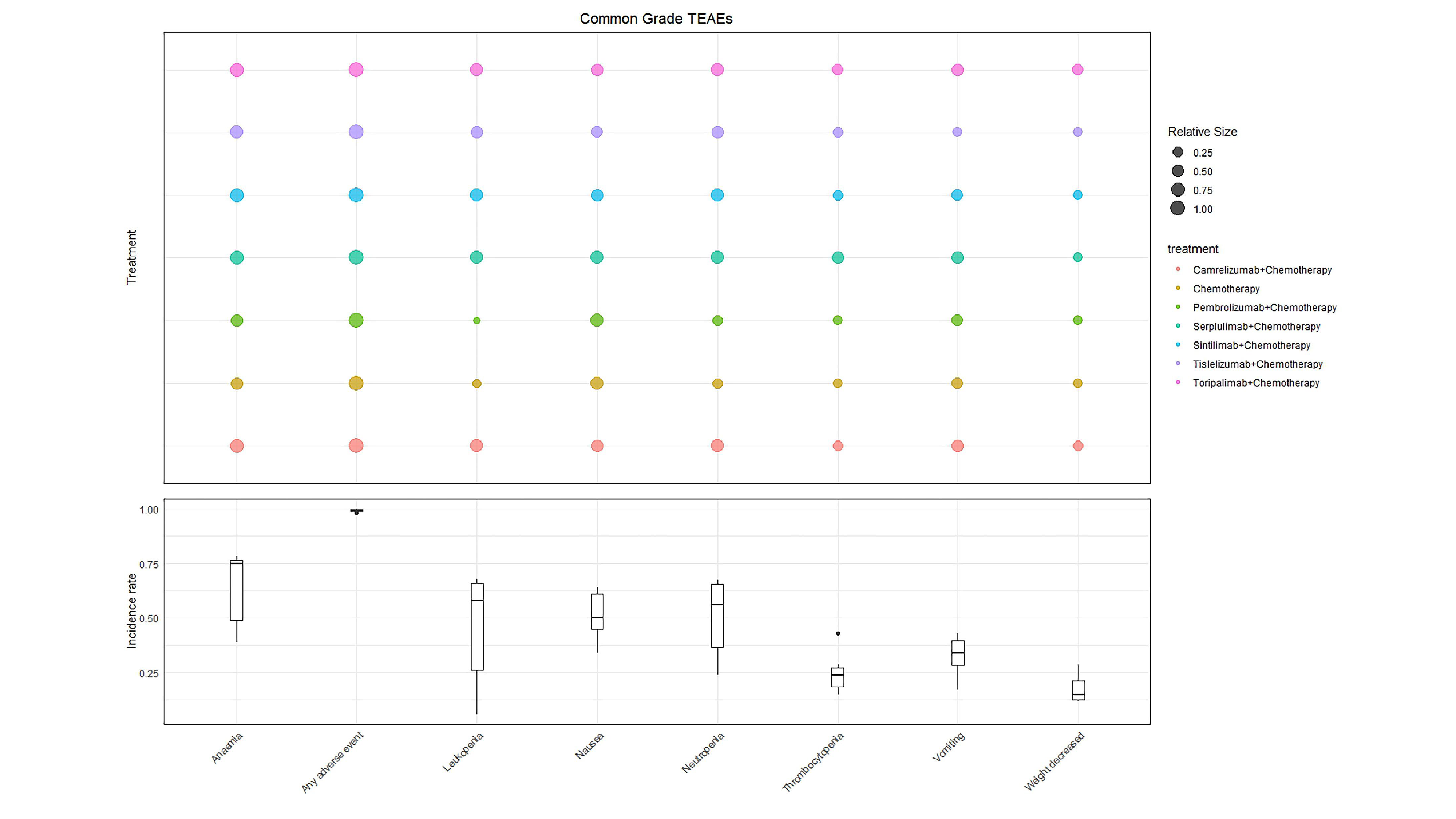

Supplement: Supplemental Material [file IANN_A_2482019_SM1981.zip › suppl_data/Figure S44. Common adverse events in 6 trials.tiff]

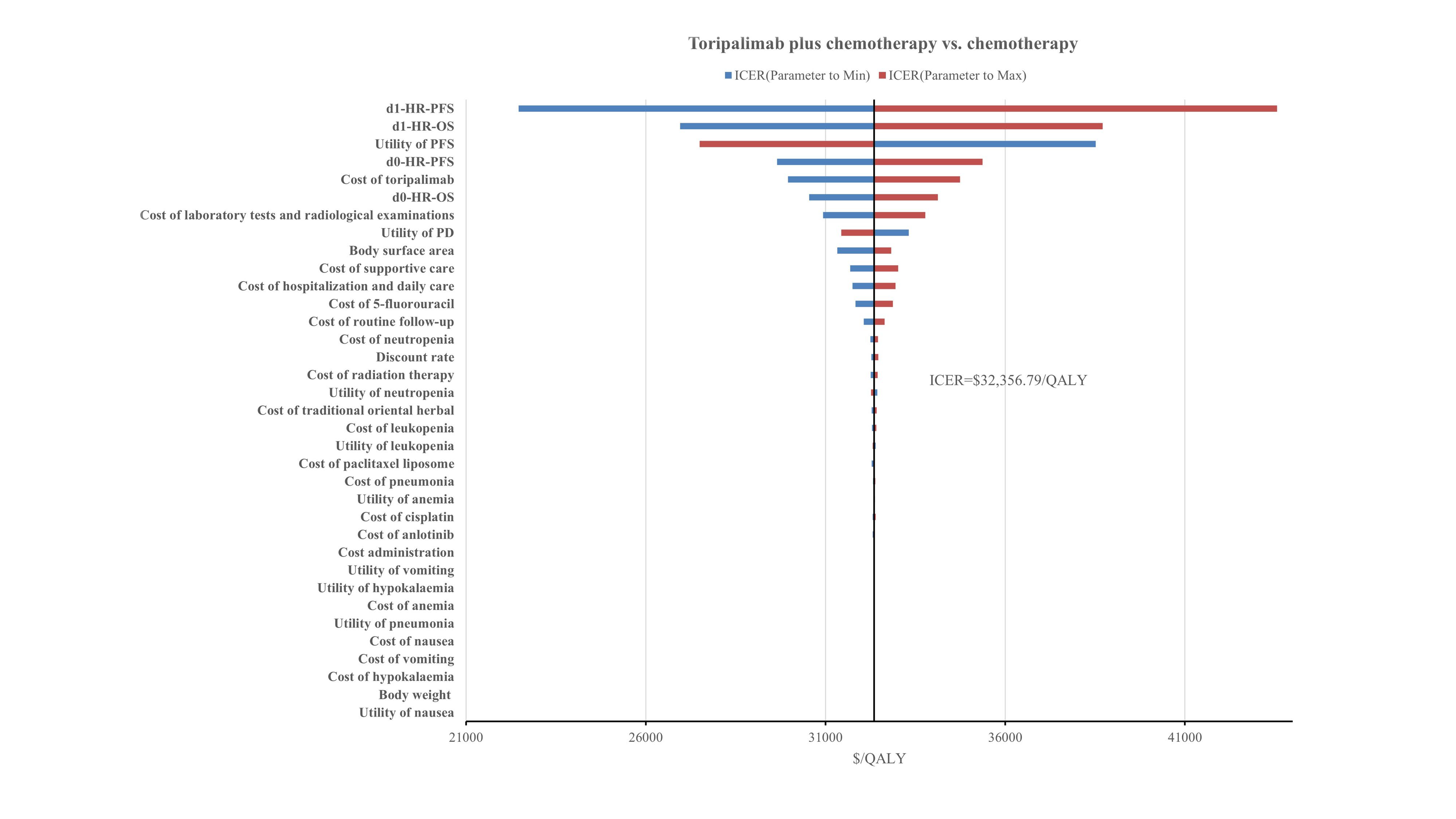

Supplement: Supplemental Material [file IANN_A_2482019_SM1981.zip › suppl_data/Figure S45. Tornado diagram of toripalimab plus chemotherapy vs. chemotherapy.tiff]

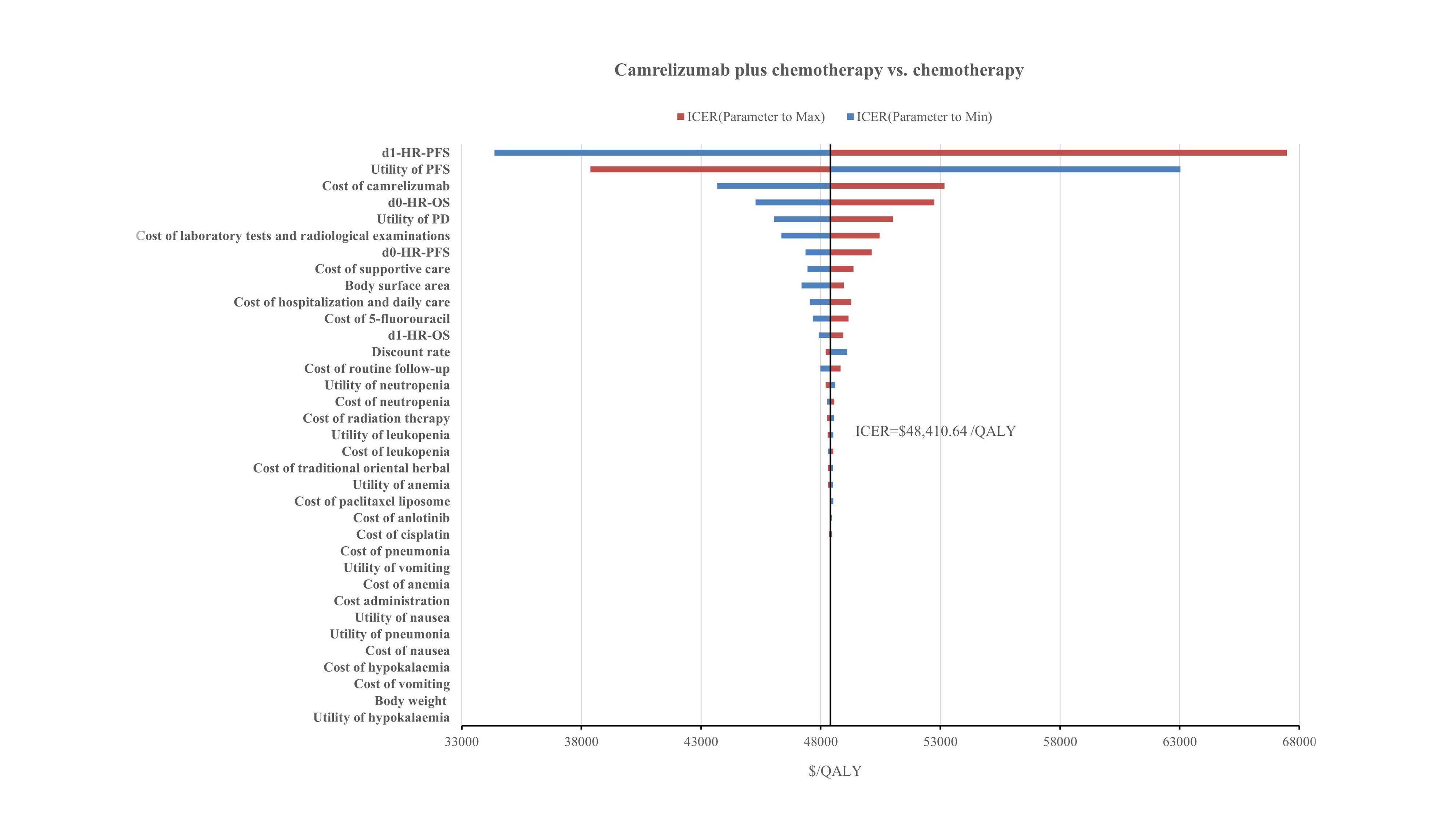

Supplement: Supplemental Material [file IANN_A_2482019_SM1981.zip › suppl_data/Figure S46. Tornado diagram camrelizumab plus chemotherapy vs. chemotherapy.tiff]

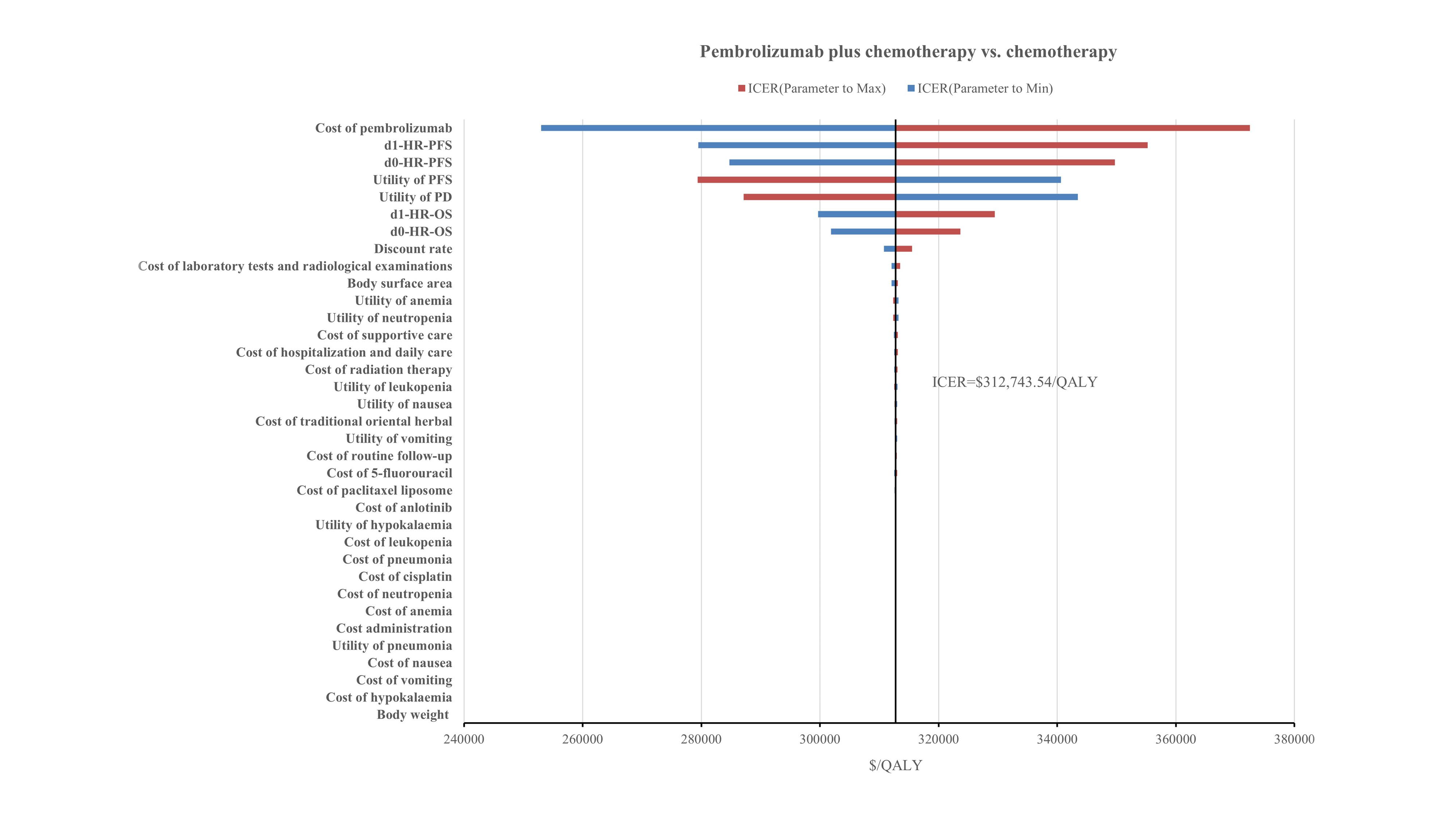

Supplement: Supplemental Material [file IANN_A_2482019_SM1981.zip › suppl_data/Figure S47. Tornado diagram pembrolizumab plus chemotherapy vs. chemotherapy.tiff]

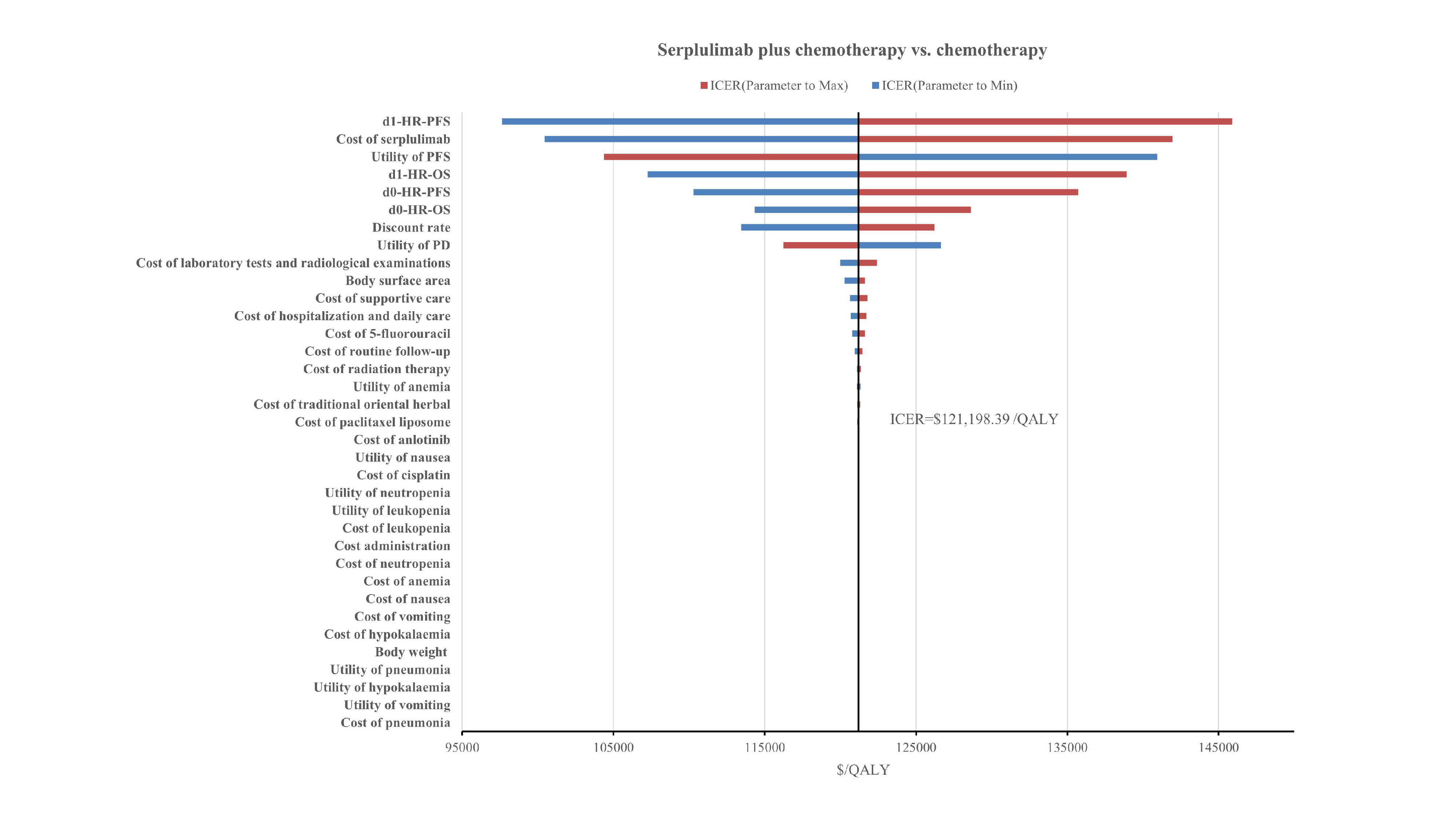

Supplement: Supplemental Material [file IANN_A_2482019_SM1981.zip › suppl_data/Figure S48. Tornado diagram of serplulimab plus chemotherapy vs. chemotherapy.tiff]

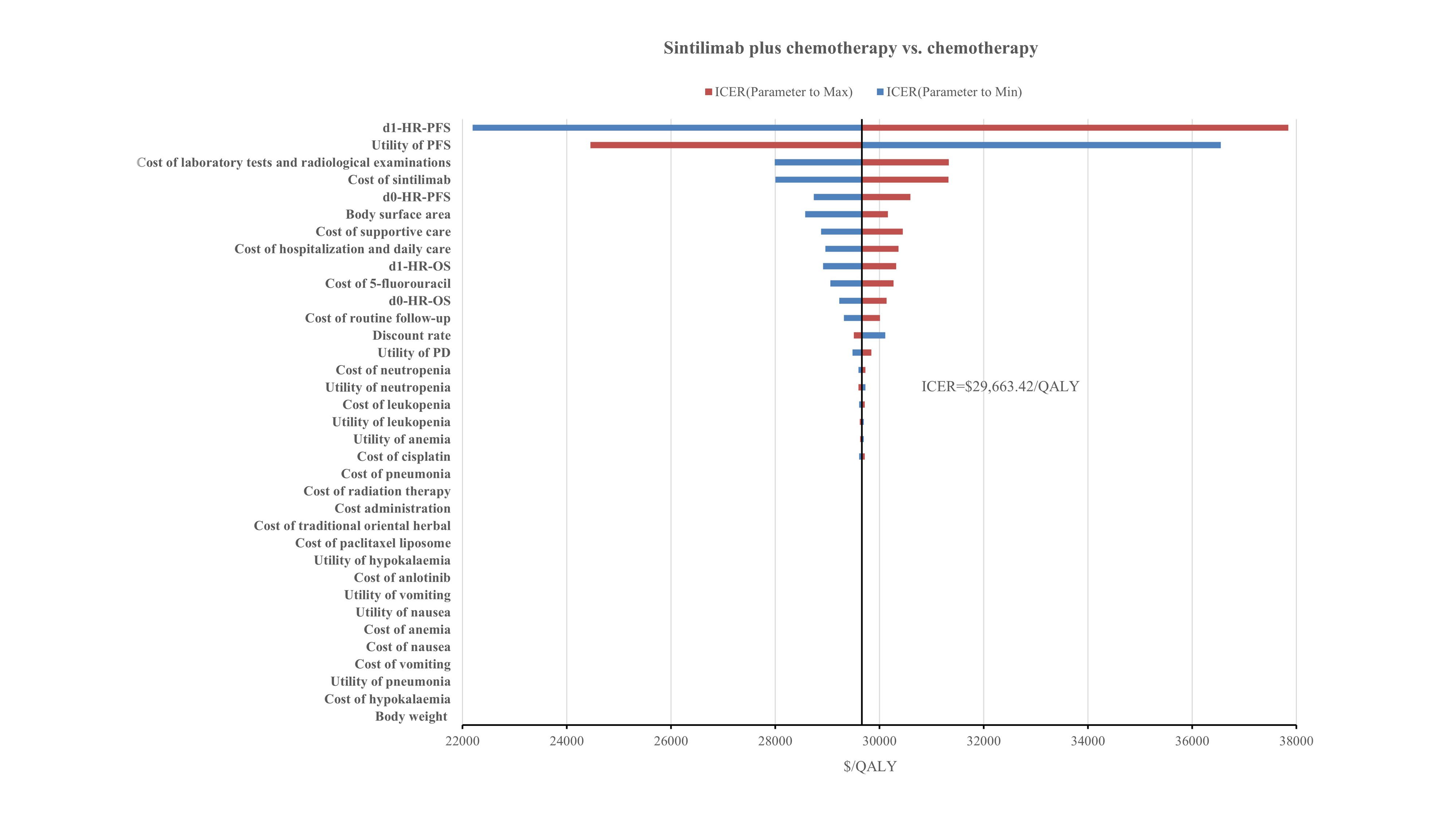

Supplement: Supplemental Material [file IANN_A_2482019_SM1981.zip › suppl_data/Figure S49. Tornado diagram of sintilimab plus chemotherapy vs. chemotherapy.tiff]

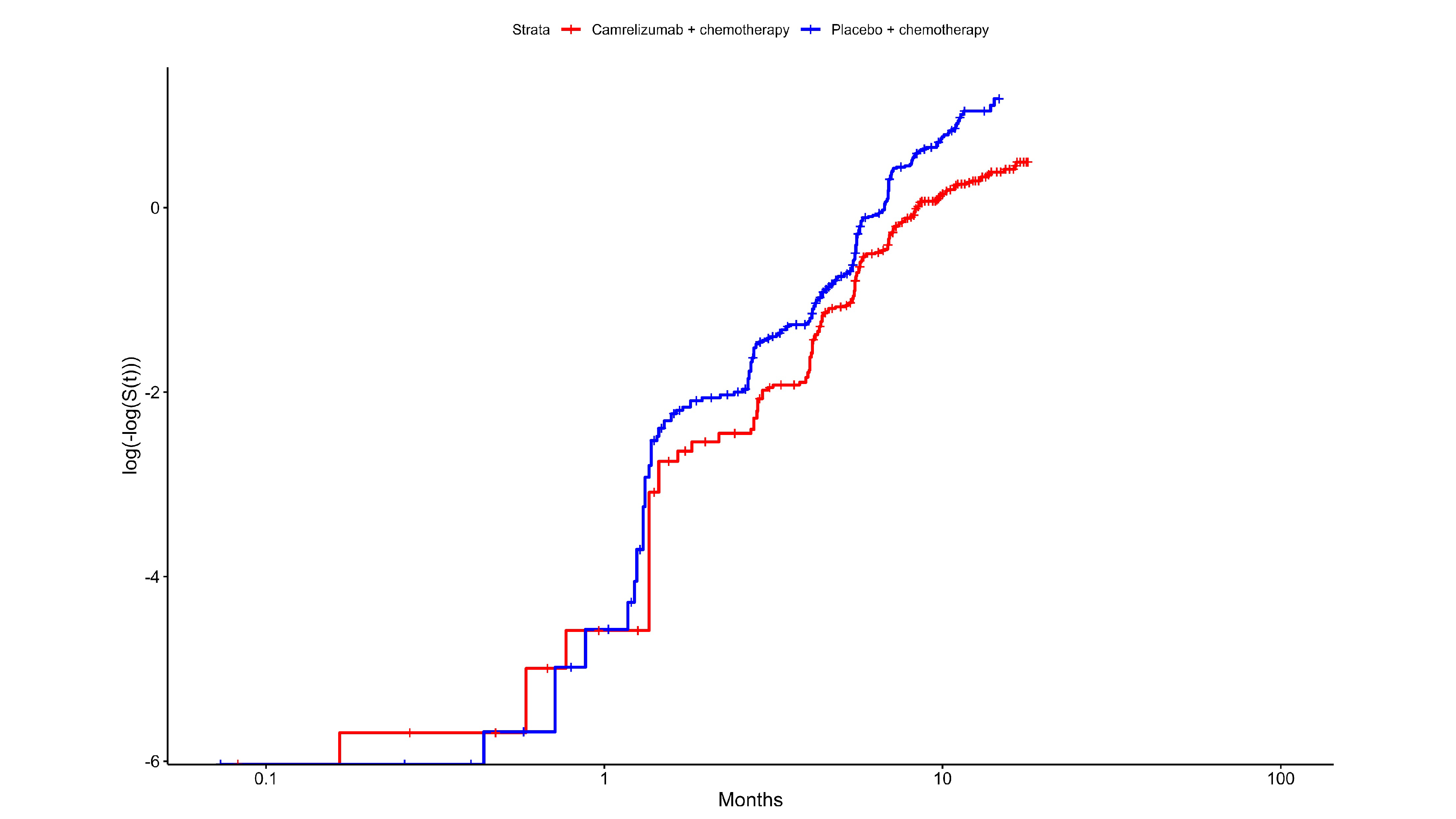

Supplement: Supplemental Material [file IANN_A_2482019_SM1981.zip › suppl_data/Figure S5. Log-cumulative plot of PFS in ESCORT-1st.tiff]

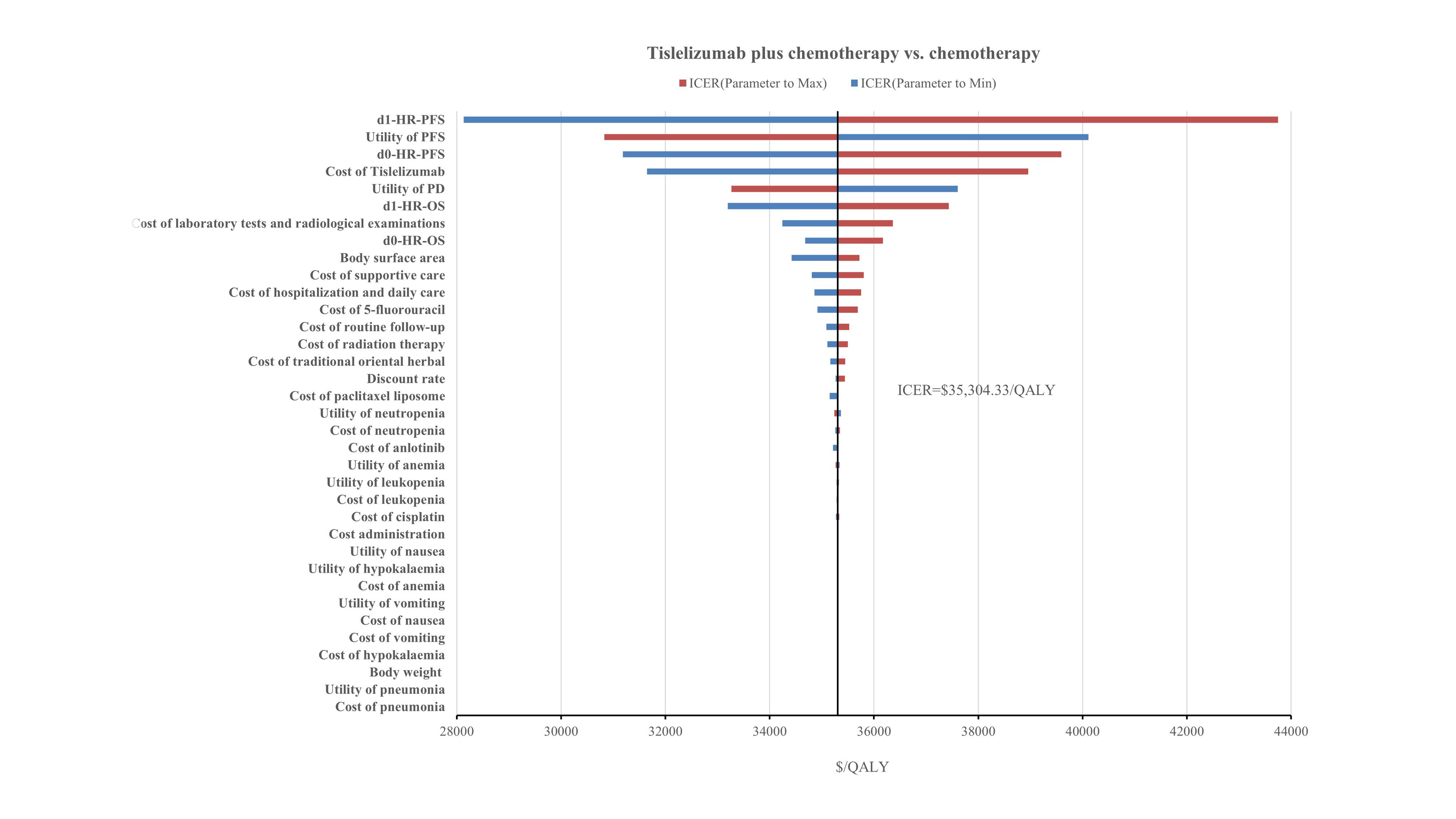

Supplement: Supplemental Material [file IANN_A_2482019_SM1981.zip › suppl_data/Figure S50. Tornado diagram of tislelizumab plus chemotherapy vs. chemotherapy.tiff]

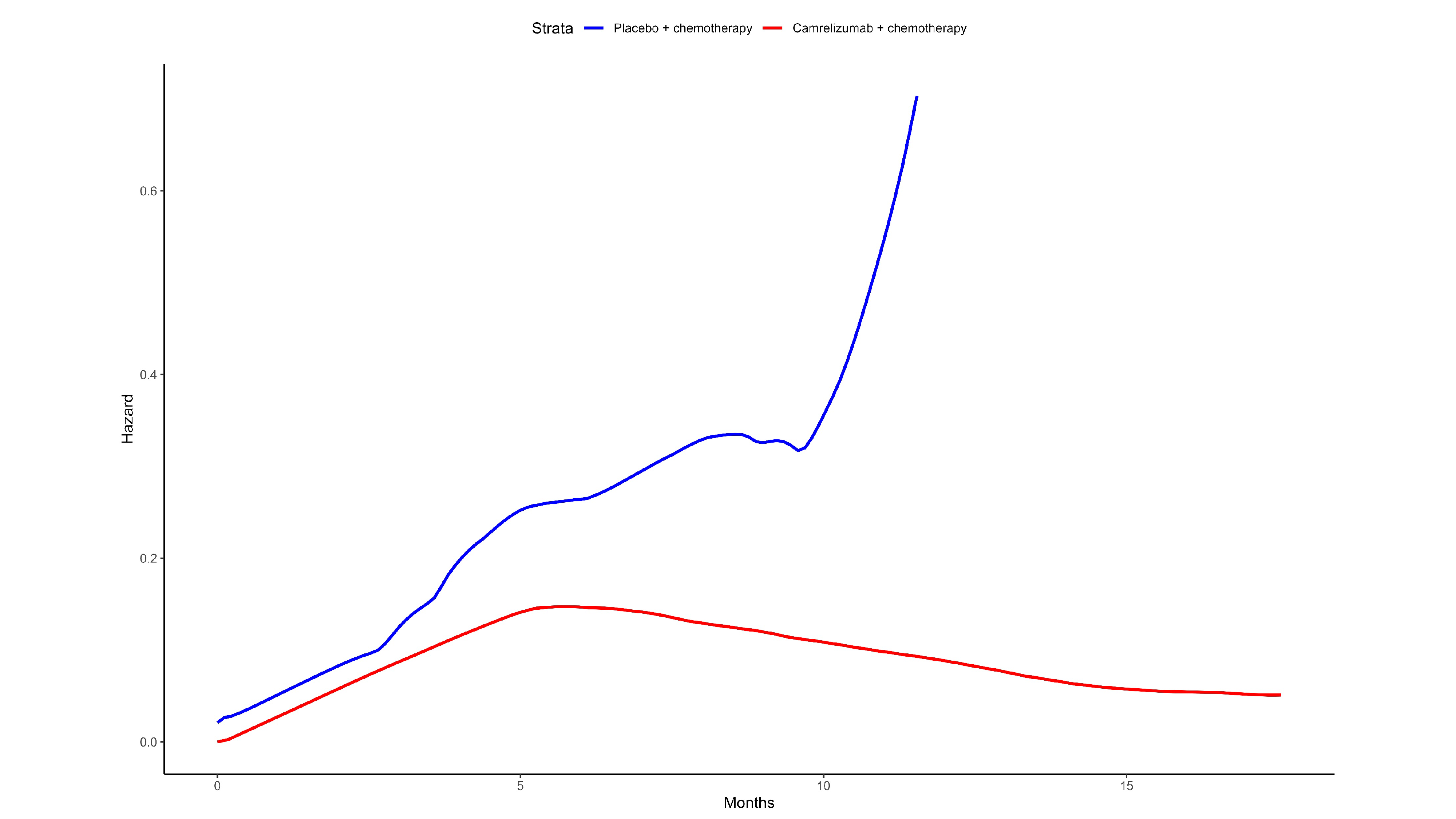

Supplement: Supplemental Material [file IANN_A_2482019_SM1981.zip › suppl_data/Figure S6 Smoothed hazard functions of PFS in ESCORT-1st.tiff]

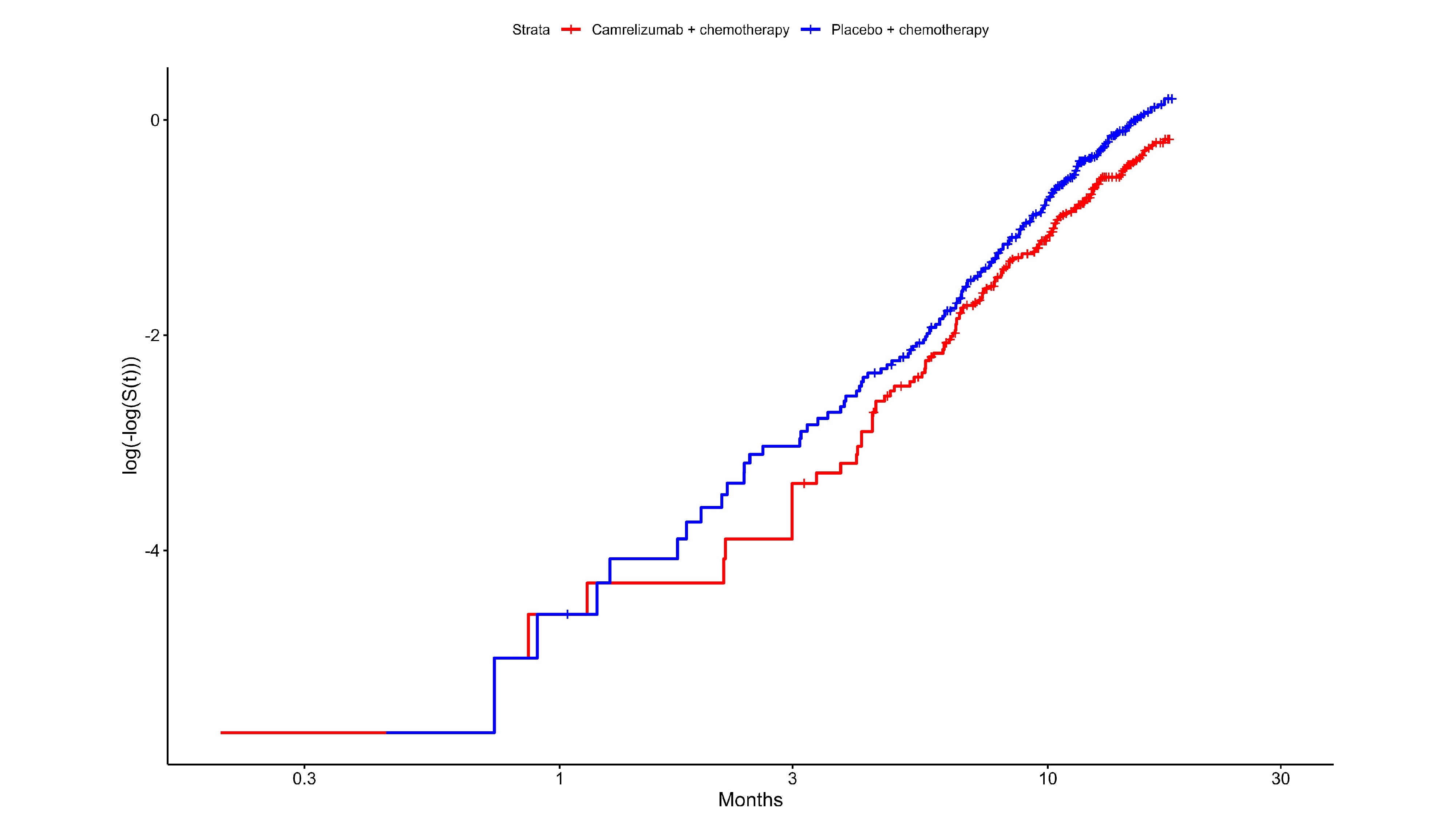

Supplement: Supplemental Material [file IANN_A_2482019_SM1981.zip › suppl_data/Figure S7. Log-cumulative plot of OS in ESCORT-1st.tiff]

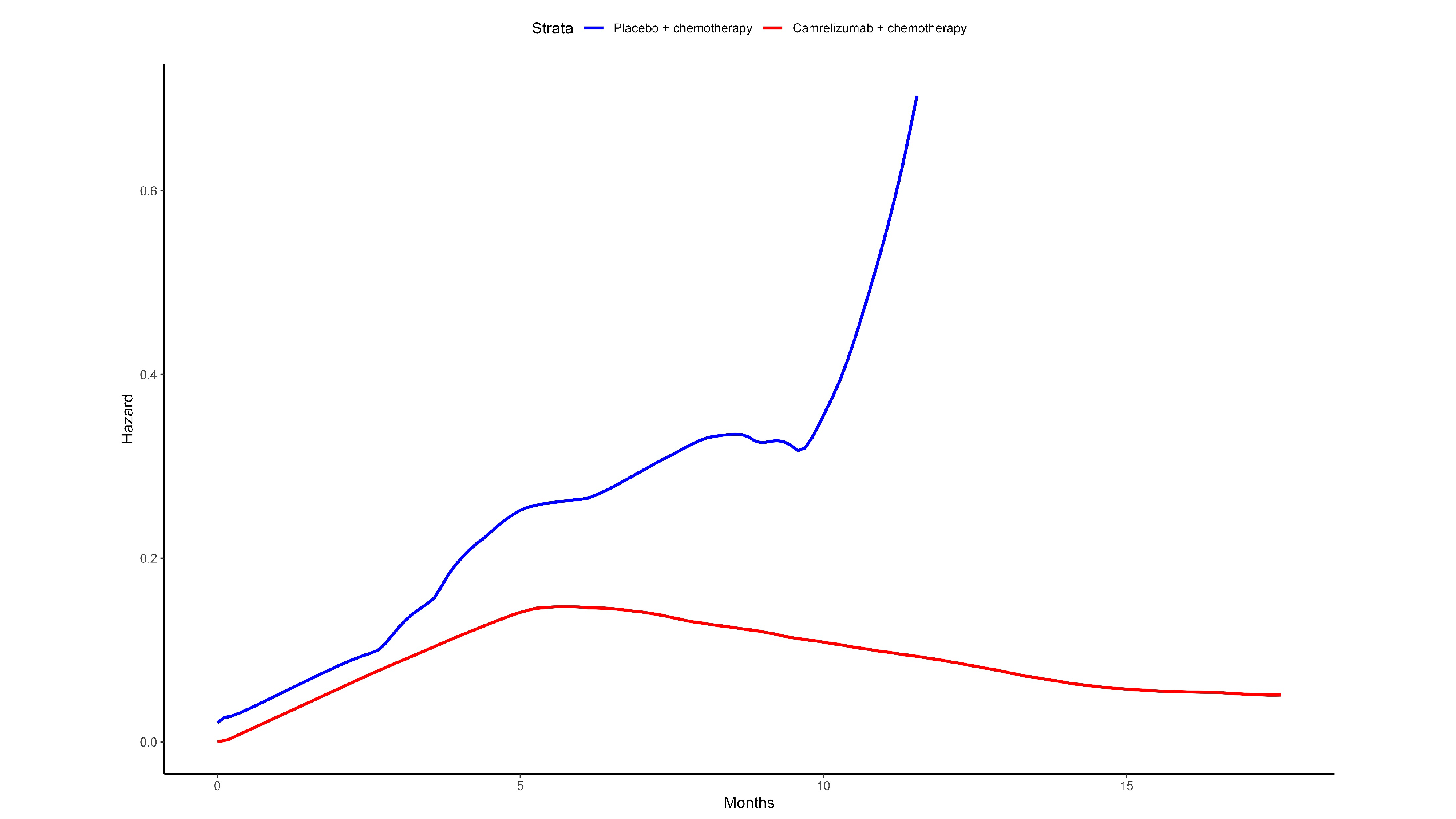

Supplement: Supplemental Material [file IANN_A_2482019_SM1981.zip › suppl_data/Figure S8. Smoothed hazard functions of OS in ESCORT-1st.tiff]

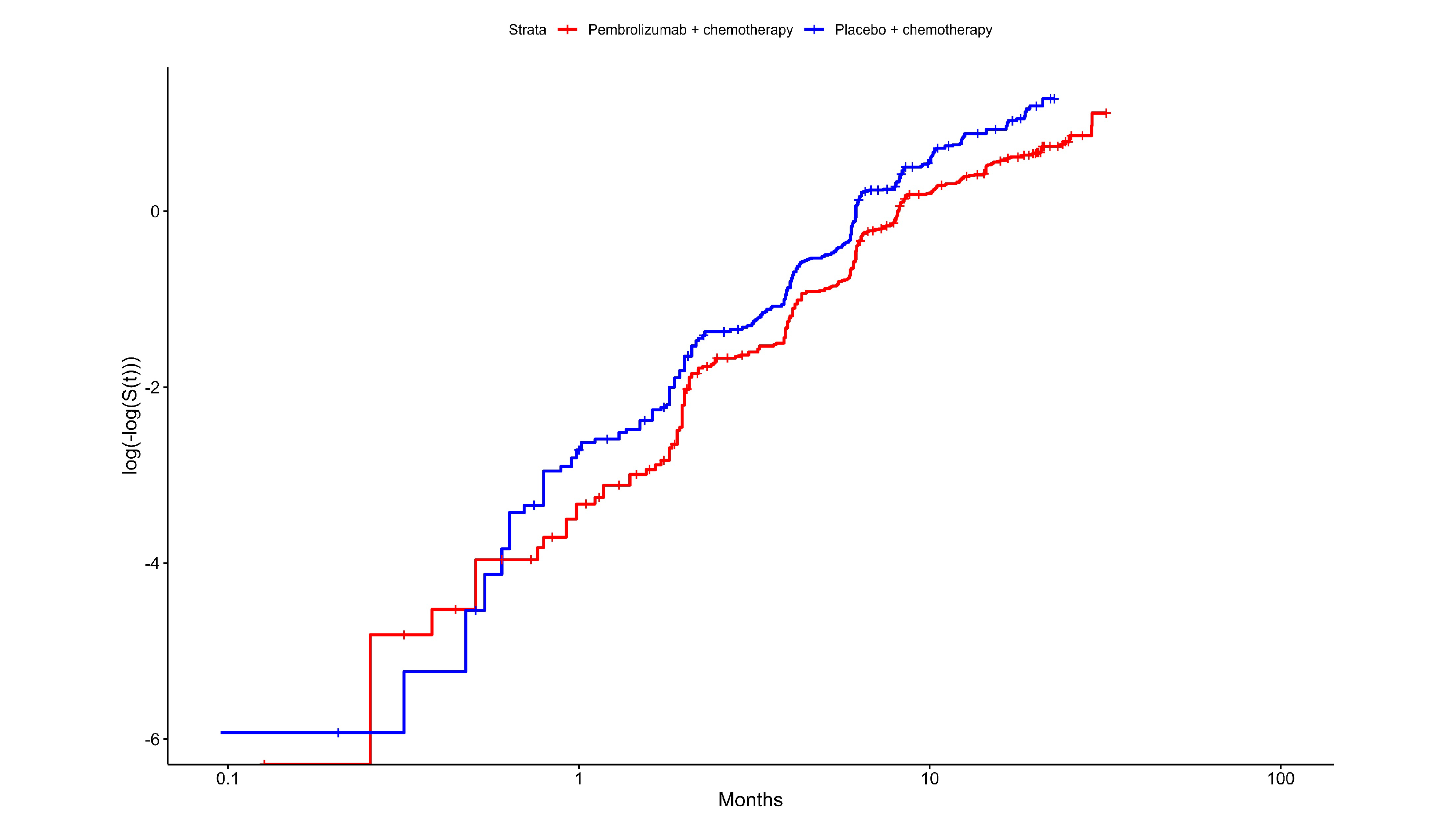

Supplement: Supplemental Material [file IANN_A_2482019_SM1981.zip › suppl_data/Figure S9. Log-cumulative plot of PFS in KEYNOTE-590.tiff]
